# Supplementary material for: An armed oncolytic virus enhances the efficacy of tumor-infiltrating lymphocyte therapy by converting tumors to artificial antigen-presenting cells in situ
Source: Mol Ther. 2022 Jun 17;30(12):3658–76. doi: 10.1016/j.ymthe.2022.06.010 (PMC9734027; doi:10.1016/j.ymthe.2022.06.010)
Supplement: Document S1. Figures S1–S15, Tables S1 and S2 [file mmc1.pdf]

YMTHE, Volume *30*

Supplemental Information

An armed oncolytic virus enhances the efficacy of tumor-infiltrating lymphocyte therapy by converting tumors to artificial antigen-presenting cells *in situ*

Kai Ye, Fan Li, Ruikun Wang, Tianyi Cen, Shiyu Liu, Zhuoqian Zhao, Ruonan Li, Lili Xu, Guanmeng Zhang, Zhaoyuan Xu, Li Deng, Lili Li, Wei Wang, Alexey Stepanov, Yajuan Wan, Yu Guo, Yuanke Li, Yuan Wang, Yujie Tian, Alexander G. Gabibov, Yingbin Yan, and Hongkai Zhang

Supplementary Figure 1

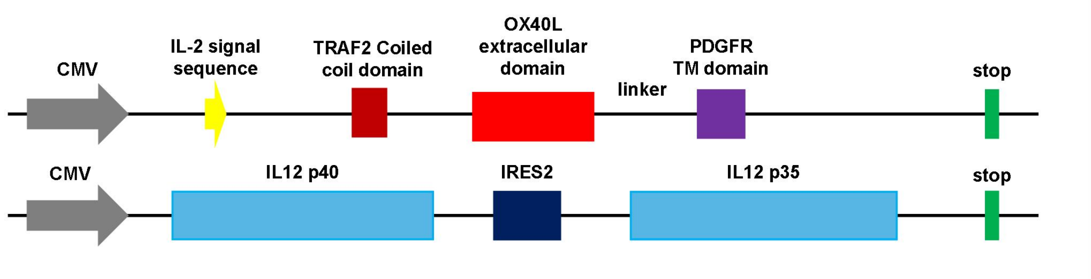

Figure S1. Schematic representation of the trimerized OX40L and IL12 design.

The OX40L extracellular domain is fused to the coiled coil trimerization domain of the TRAF2 protein and the PDGFR transmembrane[1] (TM) domain. The IL12 domain consists of IL12 p40 and IL12 p35, which are linked by internal ribosome entry site 2 (IRES2).

Supplementary Figure 2

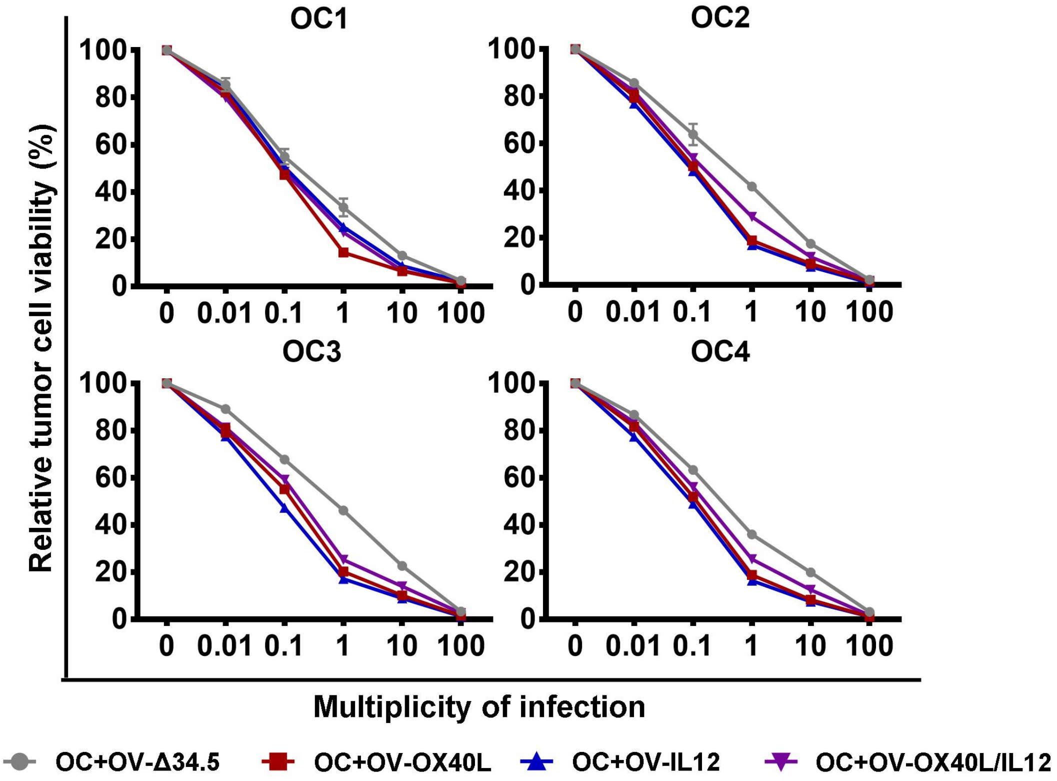

Figure S2. Susceptibility of primary cancer cells to different modified OVs.

Primary oral cancer cells from 4 patients were infected with the indicated OVs at different MOIs. Cell viability was determined at 48 h after infection by using the MTT assay (n=3).

Supplementary Figure 3

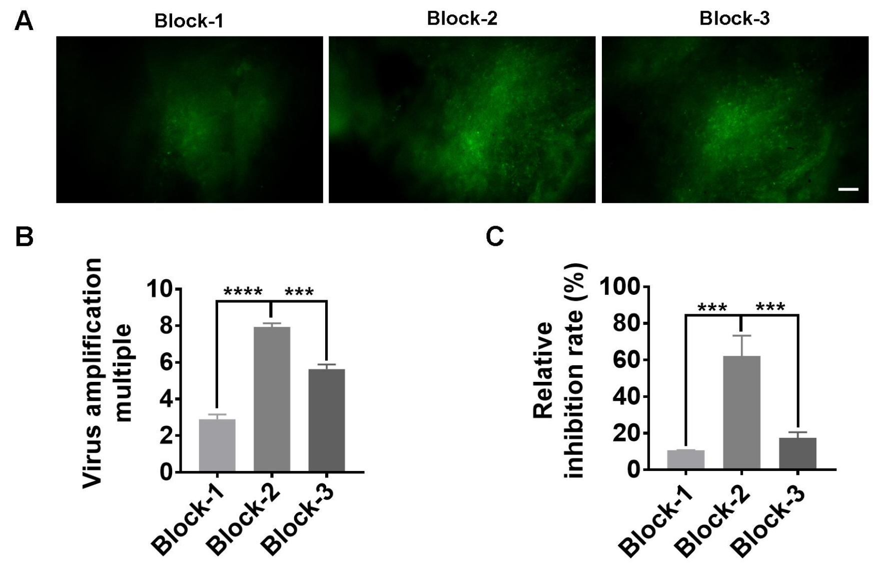

Figure S3. Susceptibility of primary oral cancer tissues to OV-GFP.

(A) Fluorescence images of 3 oral cancer tissue blocks that were infected with OV-GFP for 48 h (n=3). Scale bars, 200 μm. (B) The replication of viruses in these cancer tissues was determined by using the titration assay (n=3). (C) The viability of tumor tissues was determined with the alamar blue assay (n=3). The relative inhibition rate was calculated by dividing the alamar blue dye signal at 48 h after OV-GFP treatment by the dye signal before the addition of OV-GFP. The statistical analysis was determined by one-way ANOVA, followed by Tukey's multiple comparison test analysis. All values are presented as the mean ± SEM. ***$p < 0.001$, ****$p < 0.0001$.

70 **Supplementary Figure 4**

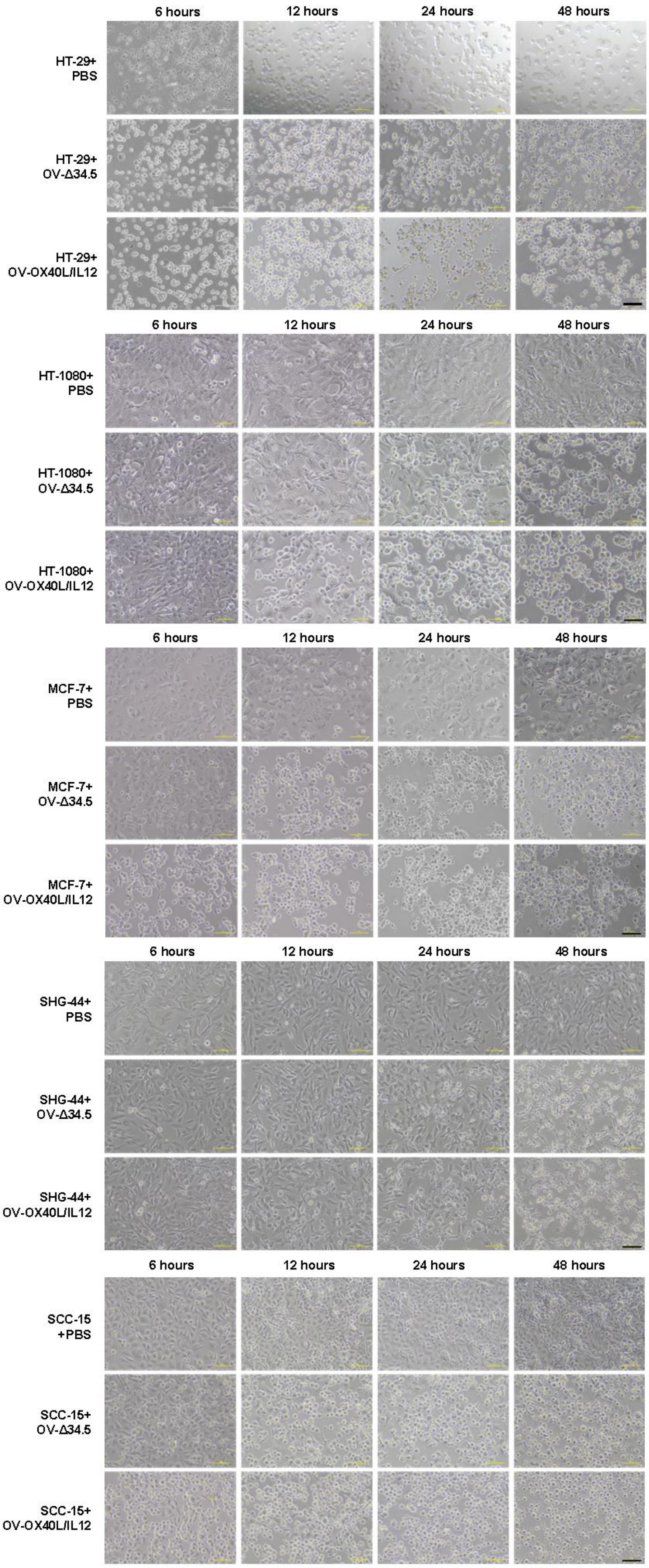

71

Figure S4. Susceptibility of primary oral cancer tissues to OV-Δ34.5 and OV-OX40L/IL12.

SHG-44, SCC-15, MCF-7, HT-1080 and HT-29 cells were cultured with OV-Δ34.5 or OV-OX40L/IL12 (MOI=0.01) for 6, 12, 24 and 48 h (n=3). Representative images showing CPEs are shown. Scale bars, 200 μm.

Supplementary Figure 5

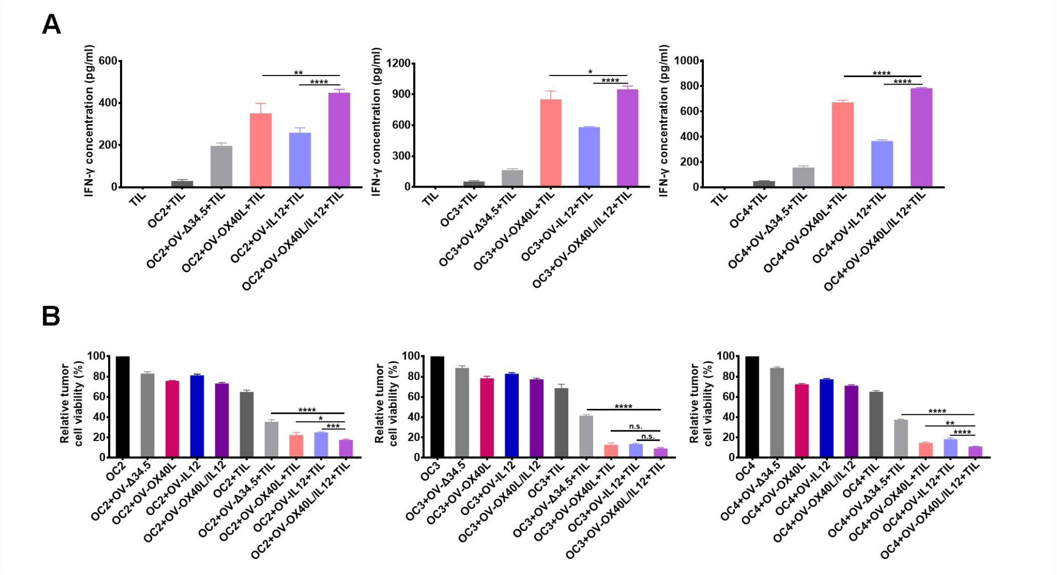

Figure S5. TILs were activated by OV-OX40L/IL12-infected tumor cells and exhibited enhanced tumoricidal activity.

Oral cancer cells from 3 patients numbered OC2, OC3 and OC4 were infected with the indicated OV and cocultured with TILs from the matched patients for 24 h. (A) IFN-γ levels in the culture supernatant were determined by using ELISA (n=3). (B) The viability of the tumor cells in coculture was assessed by using the MTT assay (n=3). Statistical significance was determined by using one-way ANOVA followed by multiple comparison test. All values are presented as the mean ± SEM. NS, not significant, *p < 0.05, **p < 0.01, ***p < 0.001 and ****p < 0.0001.

Supplementary Figure 6

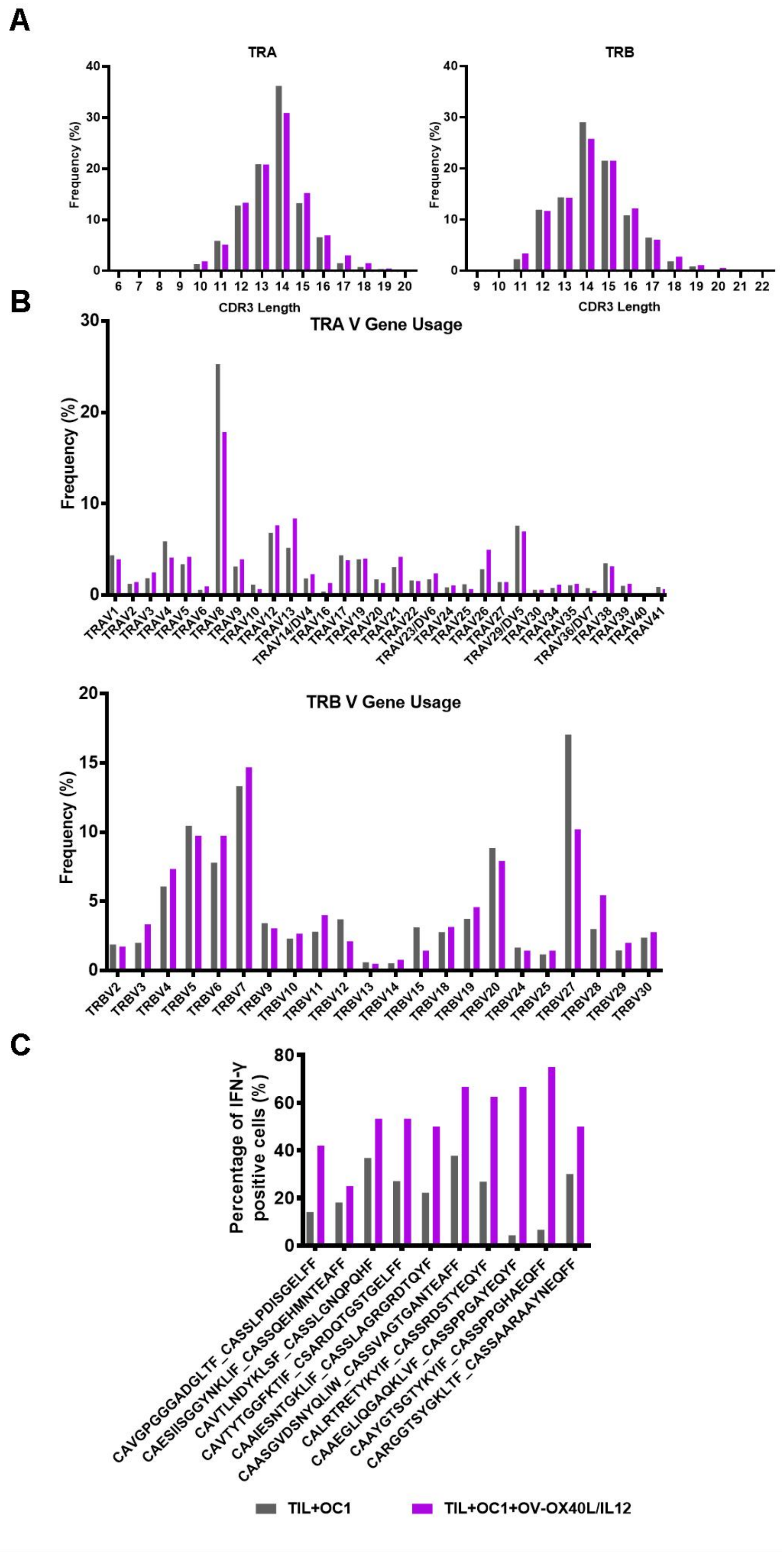

Figure S6. The length distribution of CDR3 or TRAV and TRBV germline usage for TCRs of TILs in coculture.

TILs were cocultured with mock-infected tumor cells or OV-OX40L/IL12-infected tumor cells. (A) The distribution of CDR3 length in both alpha and beta chains of TCRs. (B) TRAV and TRBV gene segment germline usage. (C) scTCR-seq and scRNA-seq were performed on TILs cocultured with OV-OX40L/IL12- or mock-infected tumor cells. The frequency of IFN-γ-positive T cells is shown for the 10 most prevalent TCR clonotypes of TILs (n=3).

Supplementary Figure 7

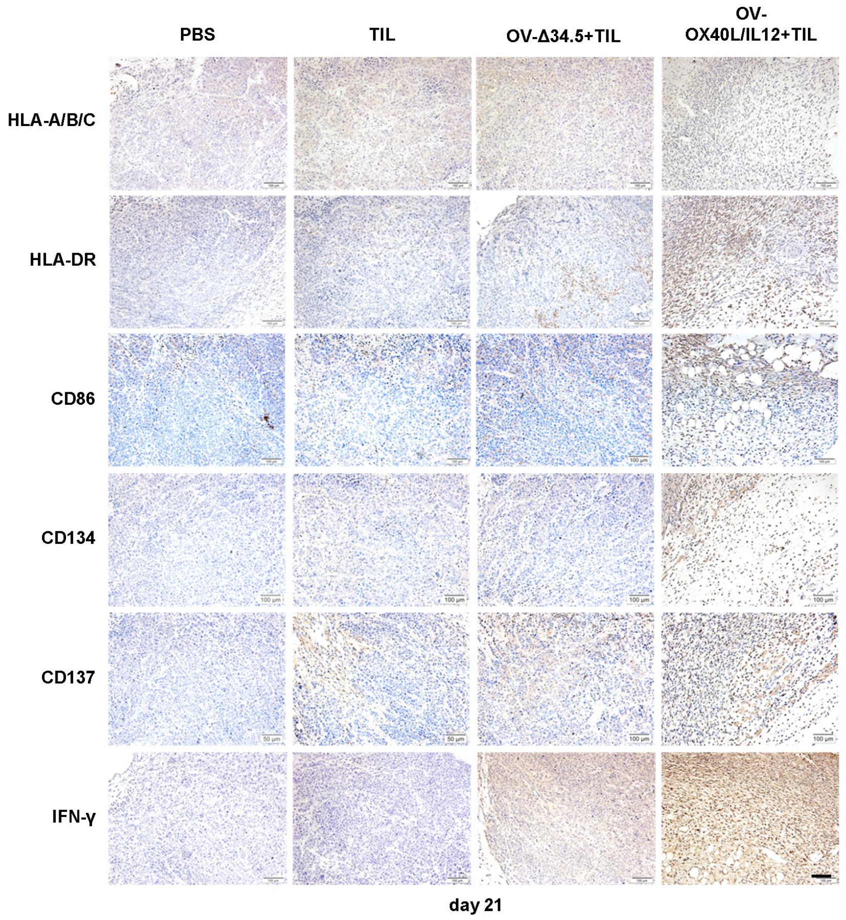

Figure S7. Representative images of IHC staining for HLA-ABC, HLA-DR, CD86, CD134, CD137 and IFN-γ in tumor tissue sections 21 days after treatment of the OC1 PDX model (n=3). Scale bars, 100 μm.

Supplementary Figure 8

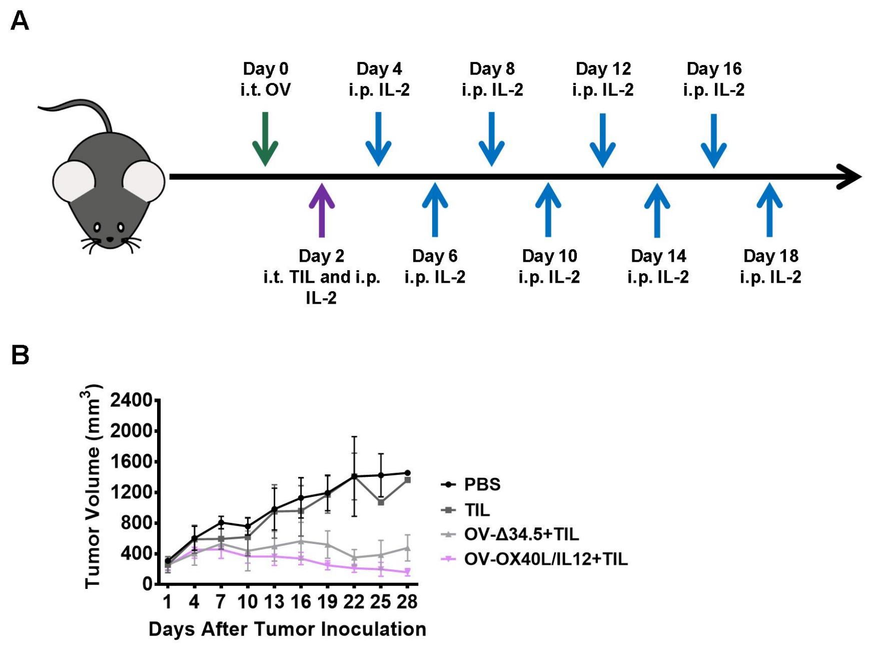

Figure S8. Antitumor efficacy of the combination therapy with OV-OX40L/IL12 and TILs in the 4th PDX tumor model (OC4 PDX model). (A) NSG mice were subcutaneously implanted with patient-derived tumor tissues, and the established tumors were intratumorally injected with the indicated OV followed by an intratumor injection of *ex vivo* expanded TILs from the same patient numbered OC4 (n=4). Tumor growth was measured every other day. The average tumor volumes (B) are shown.

Supplementary Figure 9

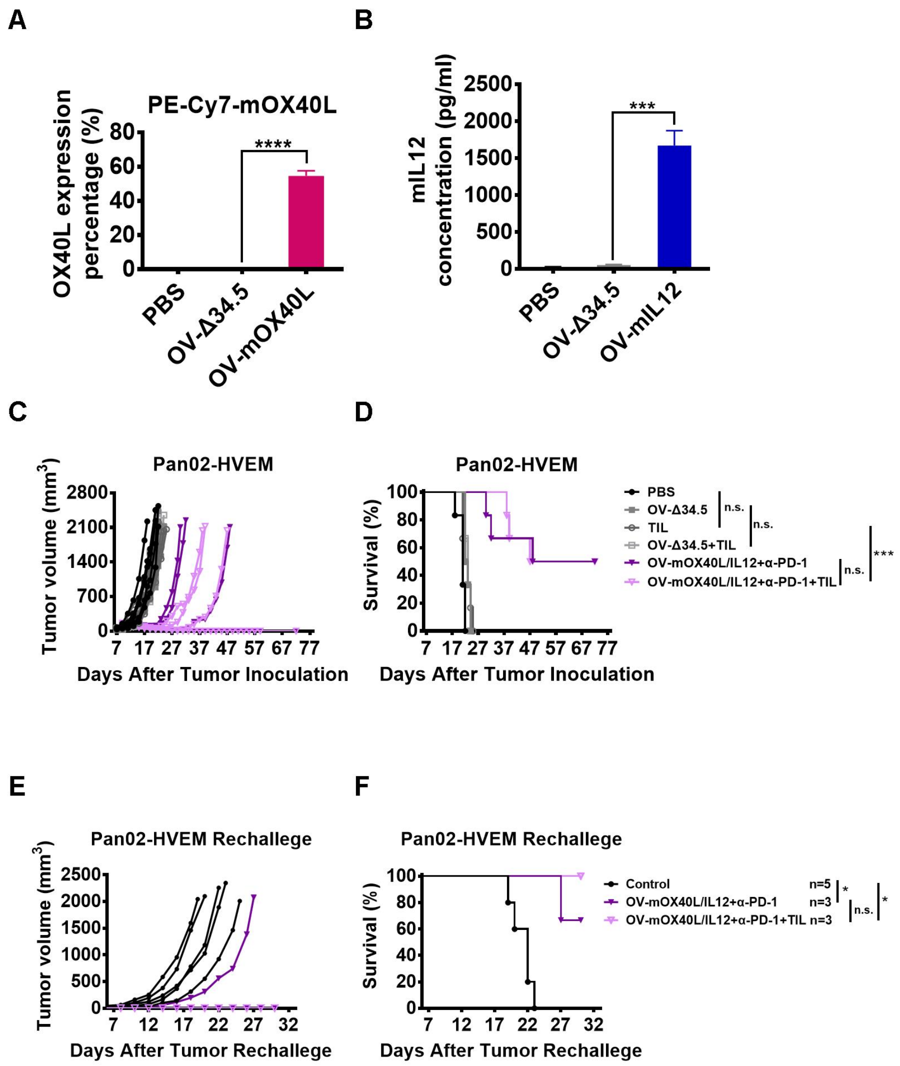

Figure S9. Combination therapy with OV-mOX40L/IL12 and TILs led to marked tumor regression in immunocompetent murine tumor models.

(A) The expression of functional mOX40L by the infected MC38 tumor cells was analyzed (n=3) by using flow cytometry. (B) Secretion of mIL12 from the infected MC38 cells was detected by using ELISA (n=3). The statistical analysis was determined by one-way ANOVA, followed by Tukey's multiple comparison test analysis. ***$p < 0.001$, ****$p < 0.0001$. (C) Tumor growth was monitored over time using electric calipers to measure tumor size in two dimensions (n=6). (D) The survival curve of Pan02-HVEM tumor-bearing mice was plotted using Kaplan-Meier analysis, and the log-rank test indicated a significant difference in the survival curves between the OV-mOX40L/IL12, PD-1 antagonist antibody and TIL combination therapy groups and the OV-Δ34.5+TIL group (n=6). Mice previously cured of Pan02-HVEM tumors by the combination therapy and age-matched treatment-naïve mice were subcutaneously inoculated with Pan02-HVEM cells. Tumor growth (E) in individual mice and the survival curve (F) are shown. All values are presented as the mean ± SEM. NS, not significant, *$p < 0.05$ and ***$p < 0.001$.

Supplementary Figure 10

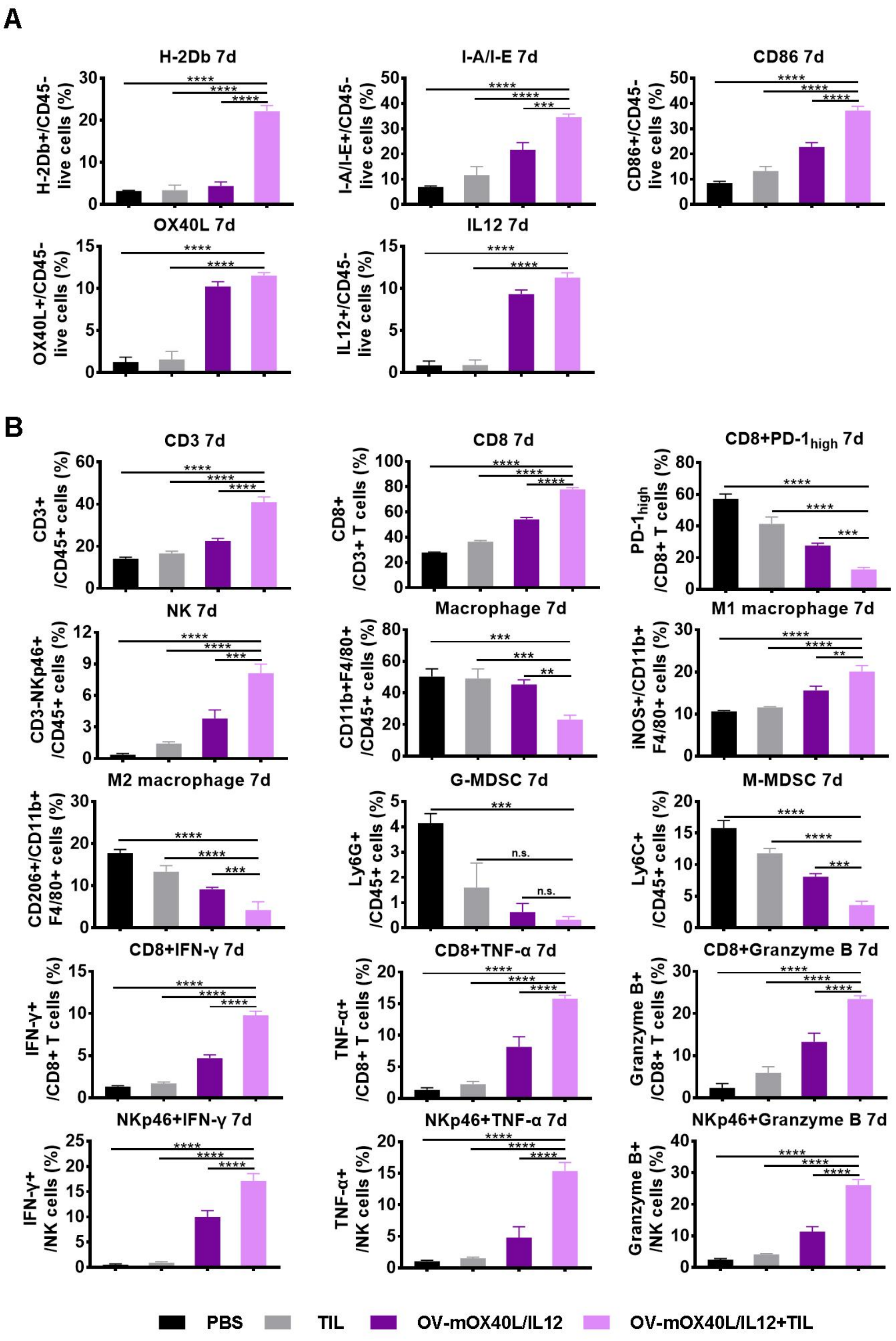

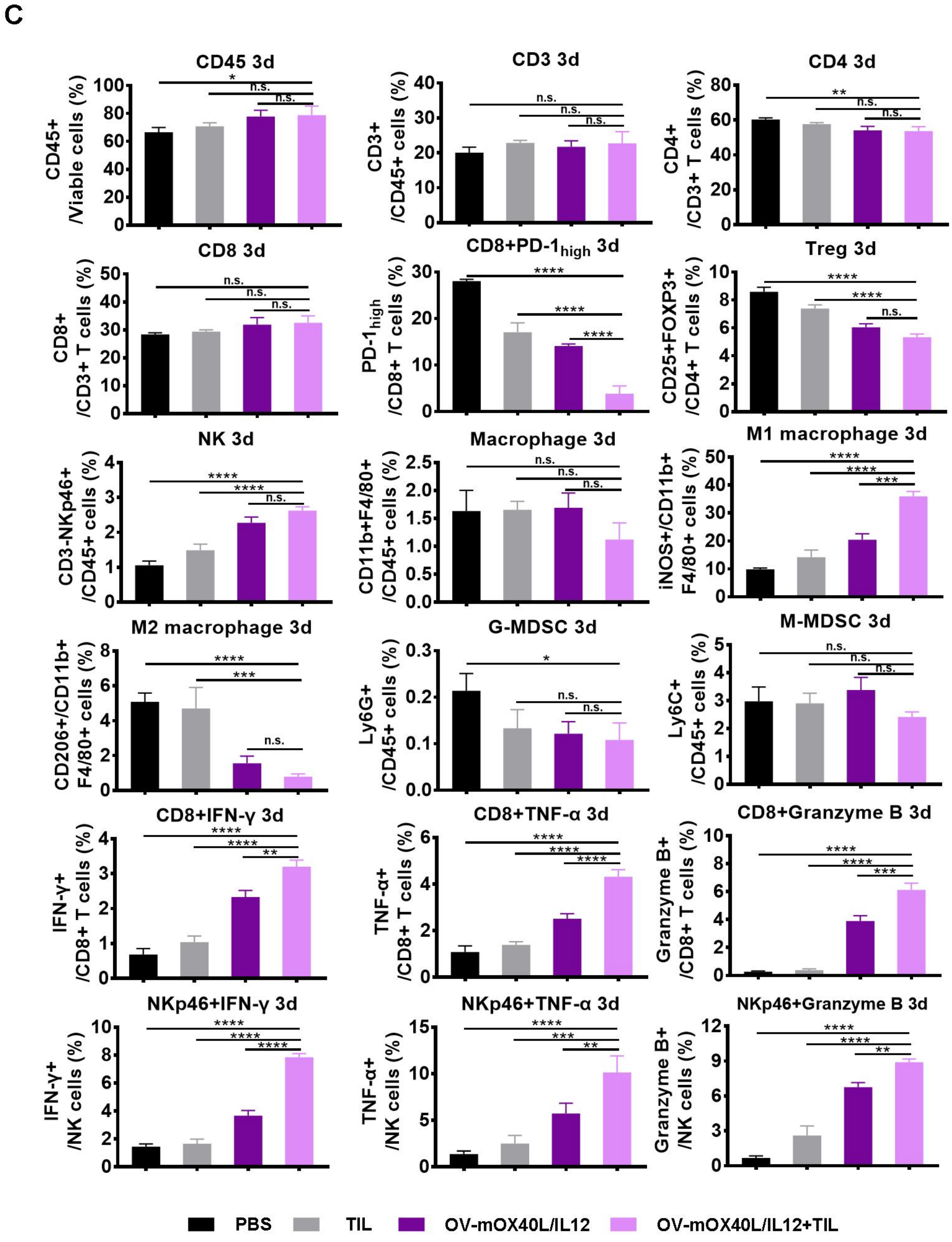
C
CD45 3d
CD45+ /Viable cells (%)
CD3 3d
CD3+ /CD45+ cells (%)
CD4 3d
CD4+ /CD3+ T cells (%)
CD8 3d
CD8+ /CD3+ T cells (%)
CD8+PD-1high 3d
PD-1high /CD8+ T cells (%)
Treg 3d
CD25+FOXP3+ /CD4+ T cells (%)
NK 3d
CD3-NKp46+ /CD45+ cells (%)
Macrophage 3d
CD11b+F4/80+ /CD45+ cells (%)
M1 macrophage 3d
iNOS+/CD11b+ F4/80+ cells (%)
M2 macrophage 3d
CD206+/CD11b+ F4/80+ cells (%)
G-MDSC 3d
Ly6G+ /CD45+ cells (%)
M-MDSC 3d
Ly6C+ /CD45+ cells (%)
CD8+IFN-γ 3d
IFN-γ+ /CD8+ T cells (%)
CD8+TNF-α 3d
TNF-α+ /CD8+ T cells (%)
CD8+Granzyme B 3d
Granzyme B+ /CD8+ T cells (%)
NKp46+IFN-γ 3d
IFN-γ+ /NK cells (%)
NKp46+TNF-α 3d
TNF-α+ /NK cells (%)
NKp46+Granzyme B 3d
Granzyme B+ /NK cells (%)
PBS
TIL
OV-mOX40L/IL12
OV-mOX40L/IL12+TIL

208

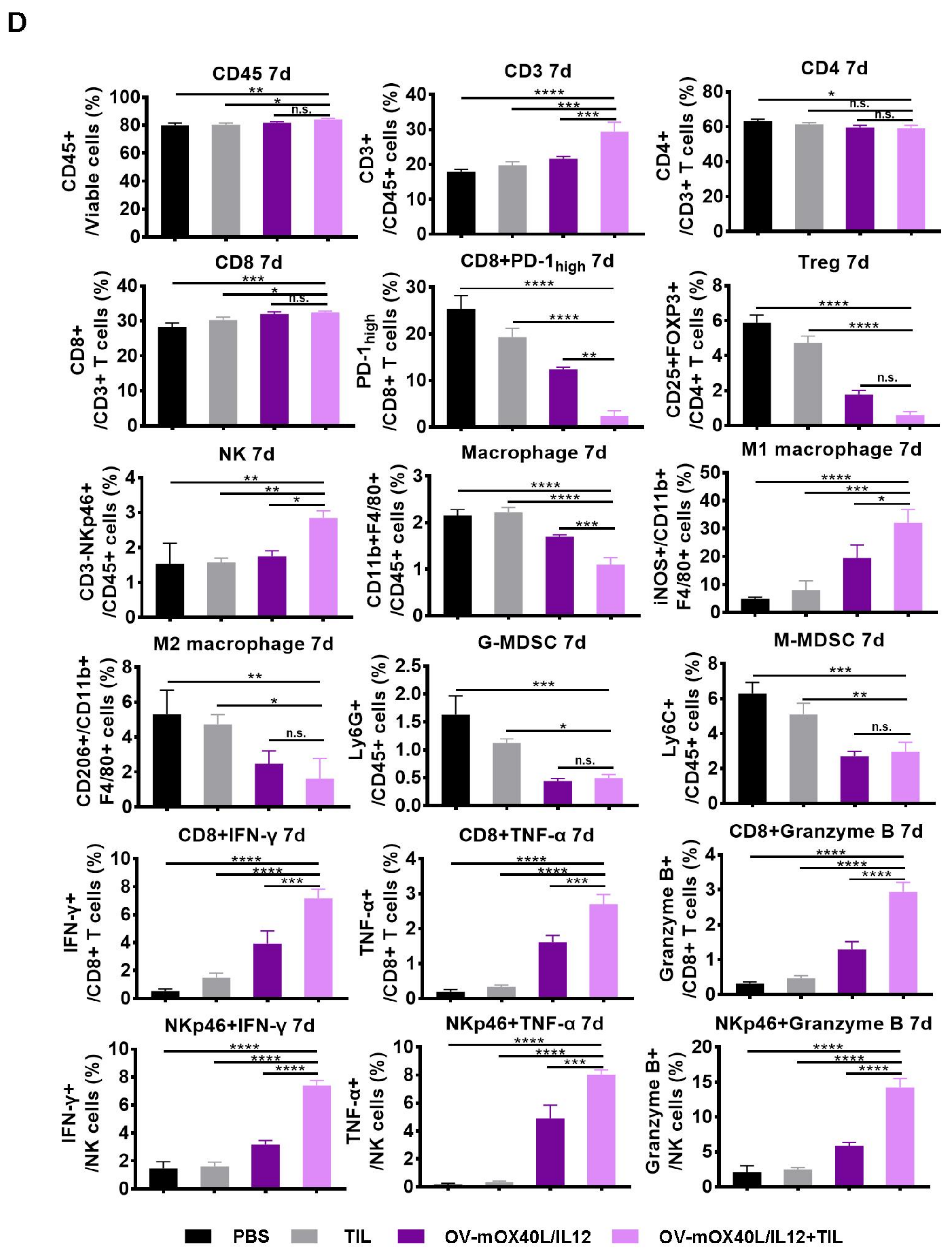
D
CD45 7d
CD45+ /Viable cells (%)
CD3 7d
CD3+ /CD45+ cells (%)
CD4 7d
CD4+ /CD3+ T cells (%)
CD8 7d
CD8+ /CD3+ T cells (%)
CD8+PD-1high 7d
PD-1high /CD8+ T cells (%)
Treg 7d
CD25+FOXP3+ /CD4+ T cells (%)
NK 7d
CD3-NKp46+ /CD45+ cells (%)
Macrophage 7d
CD11b+F4/80+ /CD45+ cells (%)
M1 macrophage 7d
iNOS+/CD11b+ F4/80+ cells (%)
M2 macrophage 7d
CD206+/CD11b+ F4/80+ cells (%)
G-MDSC 7d
Ly6G+ /CD45+ cells (%)
M-MDSC 7d
Ly6C+ /CD45+ cells (%)
CD8+IFN-γ 7d
IFN-γ+ /CD8+ T cells (%)
CD8+TNF-α 7d
TNF-α+ /CD8+ T cells (%)
CD8+Granzyme B 7d
Granzyme B+ /CD8+ T cells (%)
NKp46+IFN-γ 7d
IFN-γ+ /NK cells (%)
NKp46+TNF-α 7d
TNF-α+ /NK cells (%)
NKp46+Granzyme B 7d
Granzyme B+ /NK cells (%)
PBS
TIL
OV-mOX40L/IL12
OV-mOX40L/IL12+TIL

E

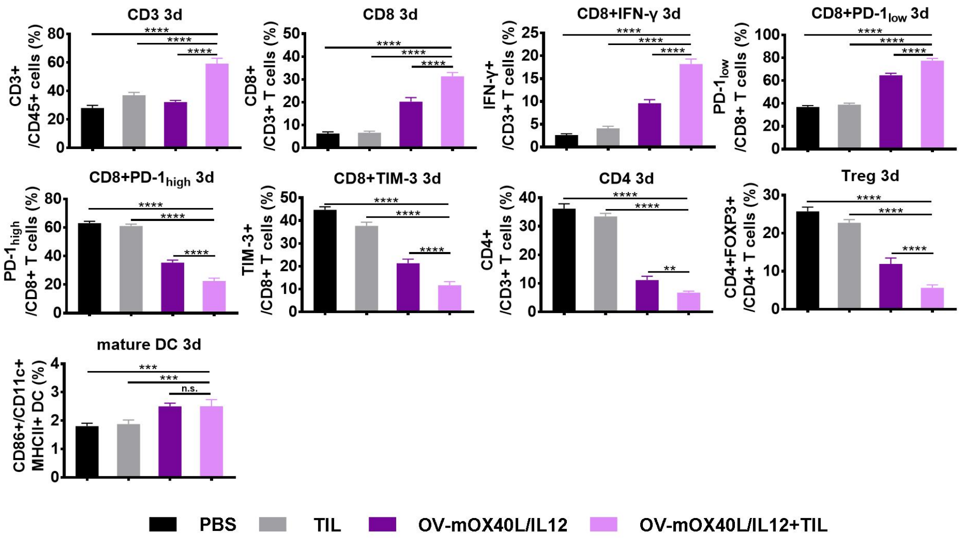
CD3 3d
CD3+ /CD45+ cells (%)
CD8 3d
CD8+ /CD3+ T cells (%)
CD8+IFN-γ 3d
IFN-γ+ /CD3+ T cells (%)
CD8+PD-1low 3d
PD-1low /CD8+ T cells (%)
CD8+PD-1high 3d
PD-1high /CD8+ T cells (%)
CD8+TIM-3 3d
TIM-3+ /CD8+ T cells (%)
CD4 3d
CD4+ /CD3+ T cells (%)
Treg 3d
CD4+FOXP3+ /CD4+ T cells (%)
mature DC 3d
CD86+/CD11c+ MHCII+ DC (%)
PBS
TIL
OV-mOX40L/IL12
OV-mOX40L/IL12+TIL

Supplementary Figure 10. Analysis of tumors, tdLNs and splenocytes after MC38-tumor-bearing mice were treated with the OV and TILs.

Mice were subcutaneously implanted with MC38 cells and treated as indicated (n=3). A single-cell suspension was prepared from tumor tissue 7 days after treatment. Tumor cells (A) and tumor infiltrating immune cells (B) were stained and subjected to flow cytometry analysis of the expression of APC-related molecules (A) and profiling of different types of immune cells and their activation status (B). A single-cell suspension was prepared from spleen tissue 3 days (C) and 7 days (D) after treatment. Splenocytes were stained and subjected to a flow cytometry analysis. (E) Flow cytometry analysis of endogenous DCs and multiple immune cell phenotypes in the tdLNs. The statistical analysis was determined by one-way ANOVA, followed by Tukey's multiple comparison test analysis. All values are presented as the mean ± SEM. NS, not significant, *$p < 0.05$, **$p < 0.01$, ***$p < 0.001$ and ****$p < 0.0001$.

Supplementary Figure 11

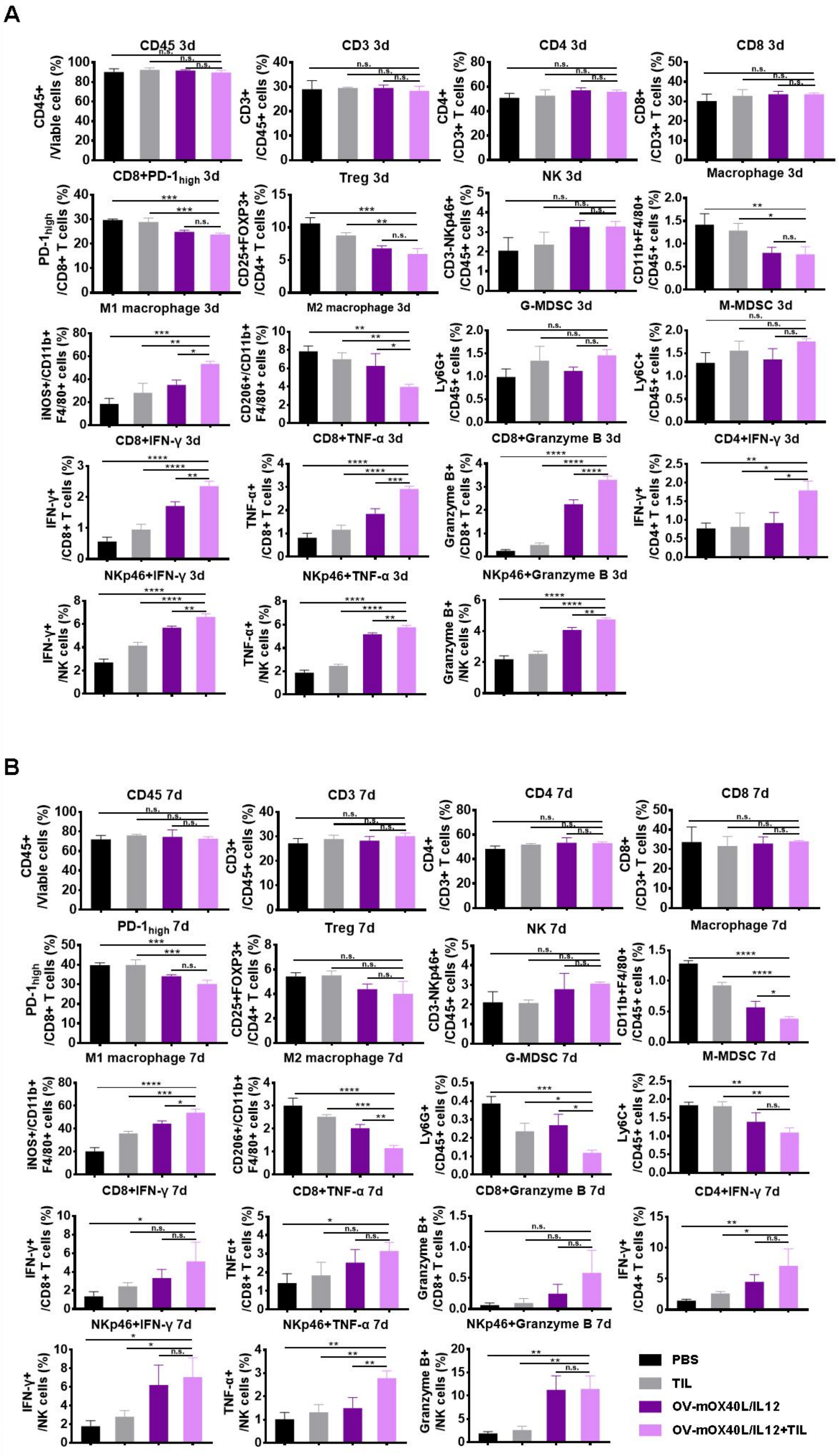

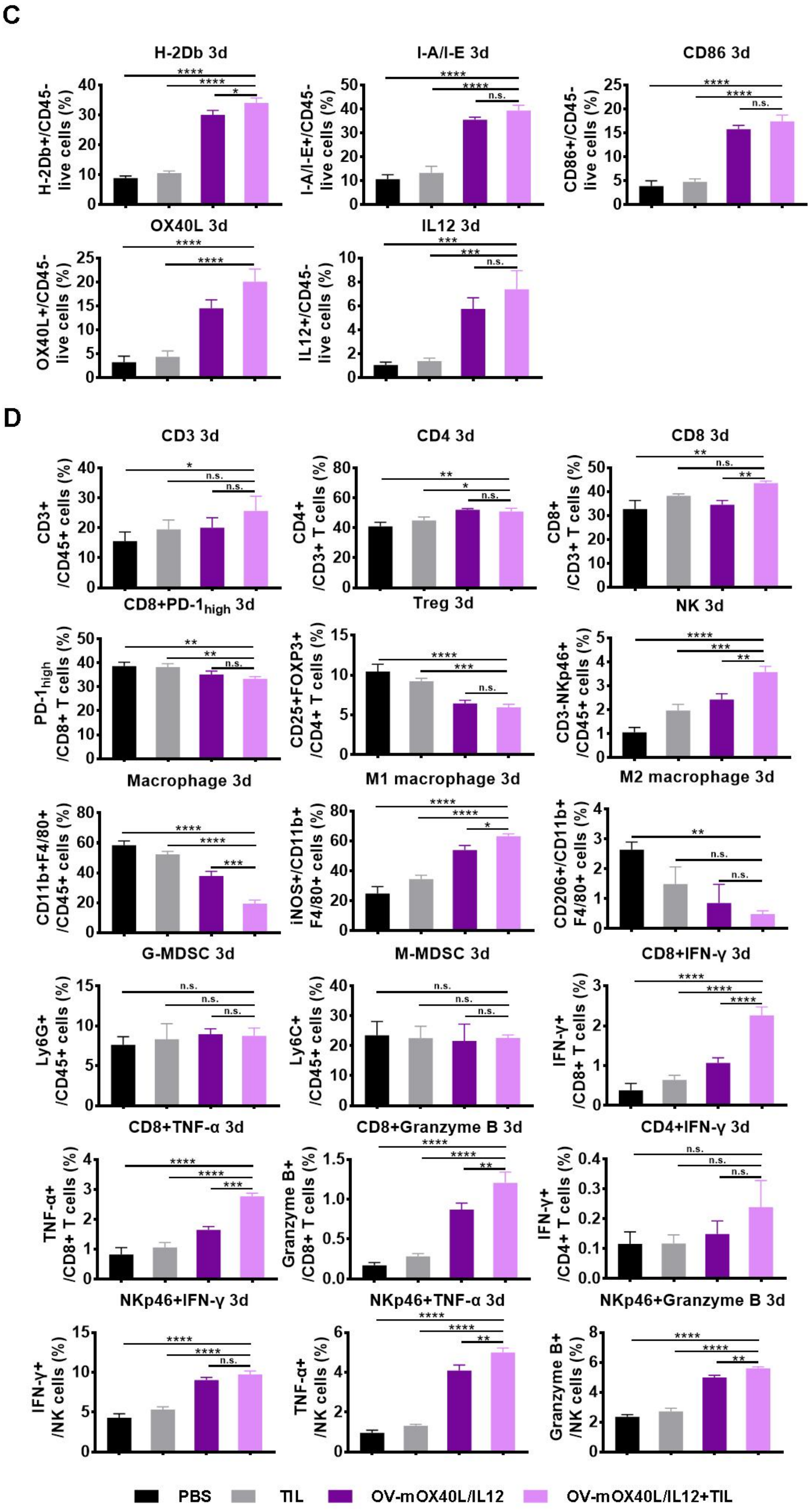
C
H-2Db 3d
H-2Db+/CD45- live cells (%)
I-A/I-E 3d
I-A/I-E+/CD45- live cells (%)
CD86 3d
CD86+/CD45- live cells (%)
OX40L 3d
OX40L+/CD45- live cells (%)
IL12 3d
IL12+/CD45- live cells (%)
D
CD3 3d
CD3+ /CD45+ cells (%)
CD4 3d
CD4+ /CD3+ T cells (%)
CD8 3d
CD8+ /CD3+ T cells (%)
CD8+PD-1high 3d
PD-1high /CD8+ T cells (%)
Treg 3d
CD25+FOXP3+ /CD4+ T cells (%)
NK 3d
CD3-NKp46+ /CD45+ cells (%)
Macrophage 3d
CD11b+F4/80+ /CD45+ cells (%)
M1 macrophage 3d
iNOS+/CD11b+ F4/80+ cells (%)
M2 macrophage 3d
CD206+/CD11b+ F4/80+ cells (%)
G-MDSC 3d
Ly6G+ /CD45+ cells (%)
M-MDSC 3d
Ly6C+ /CD45+ cells (%)
CD8+IFN-γ 3d
IFN-γ+ /CD8+ T cells (%)
CD8+TNF-α 3d
TNF-α+ /CD8+ T cells (%)
CD8+Granzyme B 3d
Granzyme B+ /CD8+ T cells (%)
CD4+IFN-γ 3d
IFN-γ+ /CD4+ T cells (%)
NKp46+IFN-γ 3d
IFN-γ+ /NK cells (%)
NKp46+TNF-α 3d
TNF-α+ /NK cells (%)
NKp46+Granzyme B 3d
Granzyme B+ /NK cells (%)
PBS
TIL
OV-mOX40L/IL12
OV-mOX40L/IL12+TIL

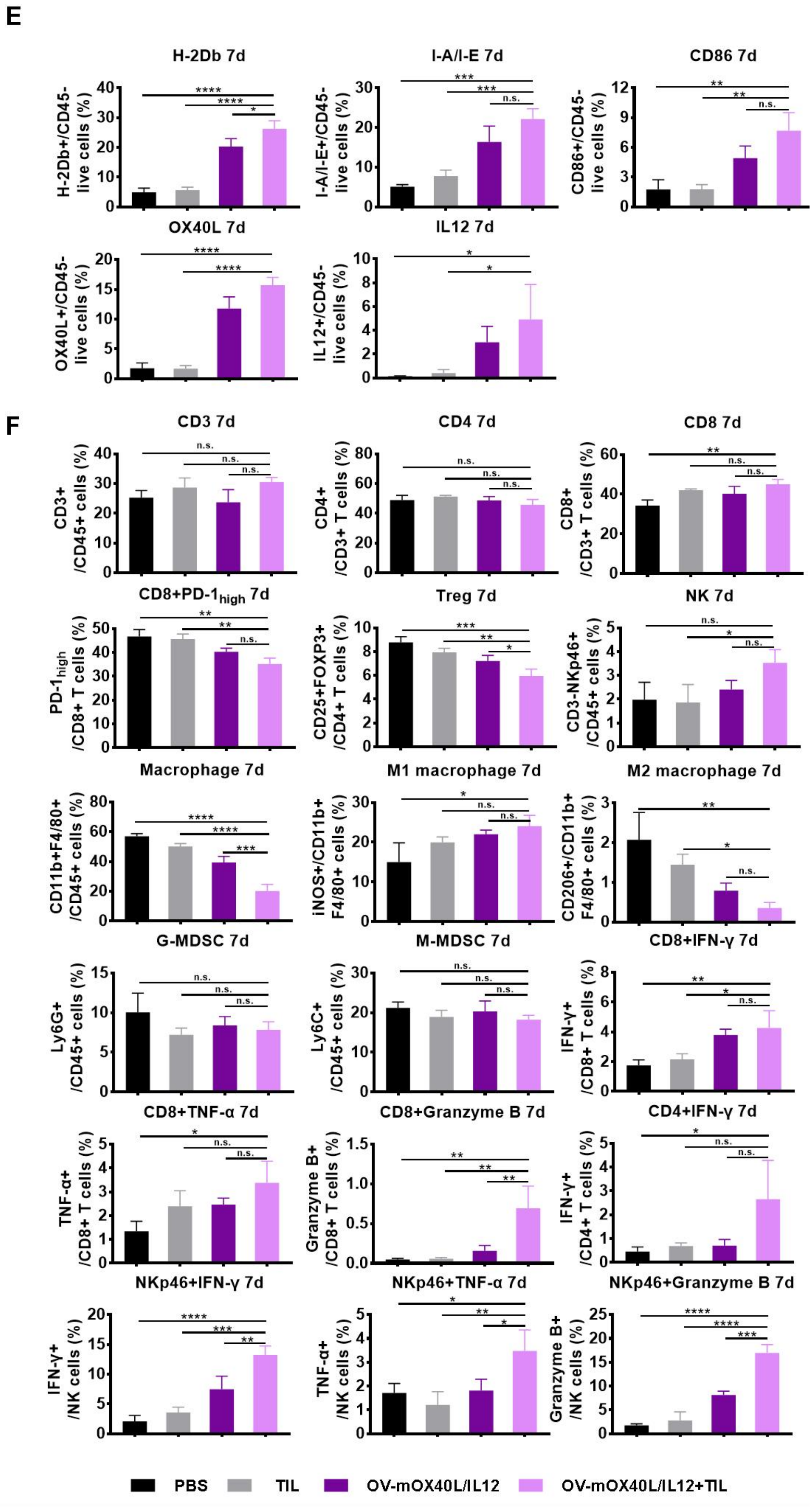
E
H-2Db 7d
H-2Db+/CD45- live cells (%)
I-A/I-E 7d
I-A/I-E+/CD45- live cells (%)
CD86 7d
CD86+/CD45- live cells (%)
OX40L 7d
OX40L+/CD45- live cells (%)
IL12 7d
IL12+/CD45- live cells (%)
F
CD3 7d
CD3+ /CD45+ cells (%)
CD4 7d
CD4+ /CD3+ T cells (%)
CD8 7d
CD8+ /CD3+ T cells (%)
CD8+PD-1high 7d
PD-1high /CD8+ T cells (%)
Treg 7d
CD25+FOXP3+ /CD4+ T cells (%)
NK 7d
CD3-NKp46+ /CD45+ cells (%)
Macrophage 7d
CD11b+F4/80+ /CD45+ cells (%)
M1 macrophage 7d
iNOS+/CD11b+ F4/80+ cells (%)
M2 macrophage 7d
CD206+/CD11b+ F4/80+ cells (%)
G-MDSC 7d
Ly6G+ /CD45+ cells (%)
M-MDSC 7d
Ly6C+ /CD45+ cells (%)
CD8+IFN-γ 7d
IFN-γ+ /CD8+ T cells (%)
CD8+TNF-α 7d
TNF-α+ /CD8+ T cells (%)
CD8+Granzyme B 7d
Granzyme B+ /CD8+ T cells (%)
CD4+IFN-γ 7d
IFN-γ+ /CD4+ T cells (%)
NKp46+IFN-γ 7d
IFN-γ+ /NK cells (%)
NKp46+TNF-α 7d
TNF-α+ /NK cells (%)
NKp46+Granzyme B 7d
Granzyme B+ /NK cells (%)
PBS
TIL
OV-mOX40L/IL12
OV-mOX40L/IL12+TIL

Figure S11. Analysis of tumors and splenocytes after Pan02-HVEM tumor-bearing mice were treated with the OV and TILs.

Mice were subcutaneously implanted with Pan02-HVEM cells and treated as indicated (n=3). A single-cell suspension was prepared from the spleen tissue 3 days (A) and 7 days (B) after treatment. Splenocytes were stained and subjected to a flow cytometry analysis. A single-cell suspension was prepared from tumor tissues 3 days (C, D) and 7 days (E, F) after treatment. Tumor cells (C, E) and tumor-infiltrating mononuclear cells (D, F) were stained and subjected to a flow cytometry analysis to determine the expression of APC-related molecules (C, E), and the profiles of different types of immune cells and their activation status are presented (D, F). The statistical analysis was determined by one-way ANOVA, followed by Tukey's multiple comparison test analysis. All values are presented as the mean ± SEM. NS, not significant, $*p < 0.05$, $**p < 0.01$, $***p < 0.001$ and $****p < 0.0001$.

Supplementary Figure 12

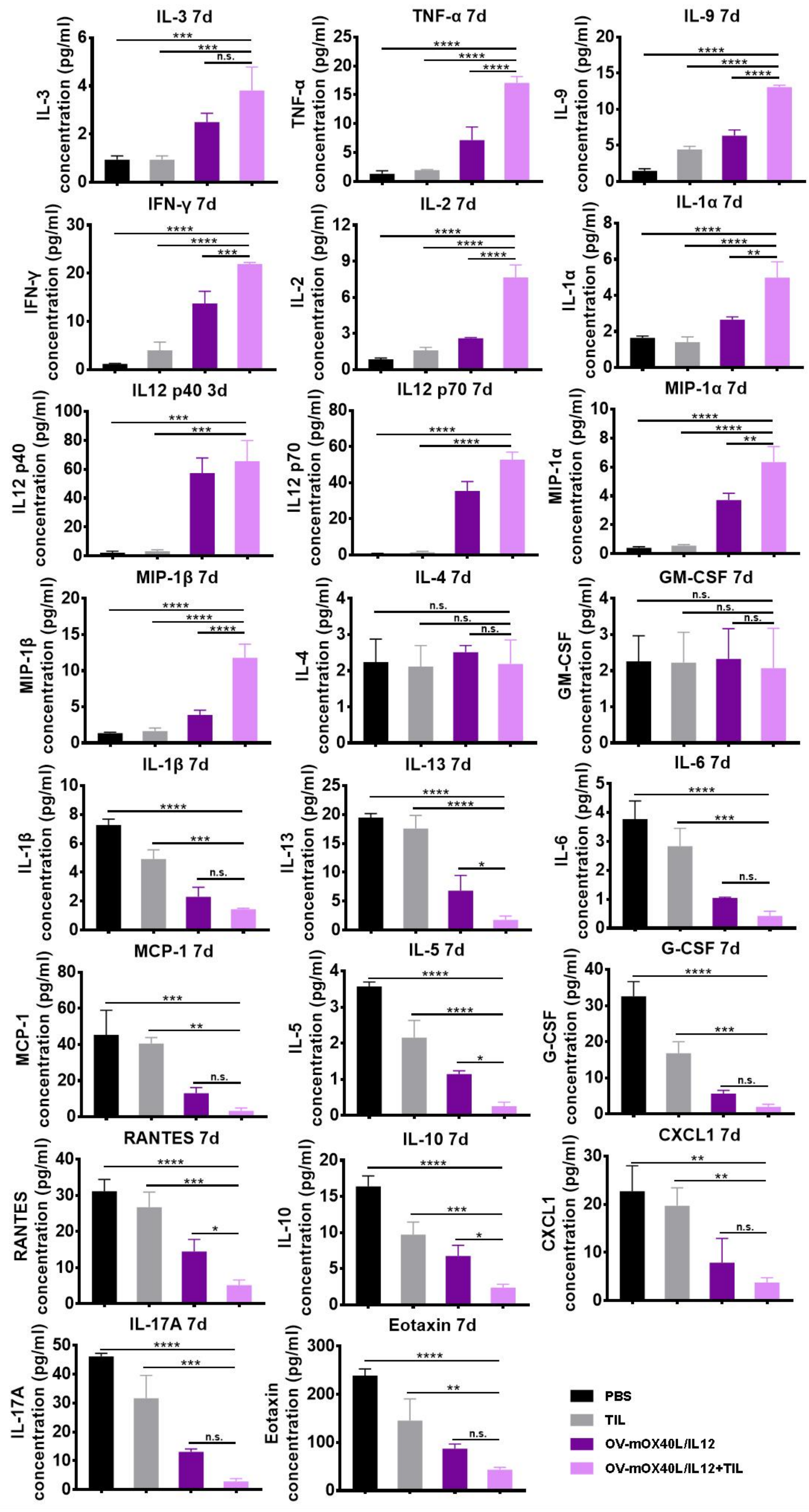

Figure S12. Profiles of cytokines and chemokines in serum samples from treated mice.

Mice were subcutaneously implanted with MC38 cells and treated as indicated (n=3). Cytokine concentrations in serum collected 7 days after treatment were determined using a Bio-Plex Mouse Cytokine 23-Plex Array. The statistical analysis was determined by one-way ANOVA, followed by Tukey's multiple comparison test analysis. All values are presented as the mean ± SEM. NS, not significant, *$p < 0.05$, **$p < 0.01$, ***$p < 0.001$ and ****$p < 0.0001$.

Supplementary Figure 13

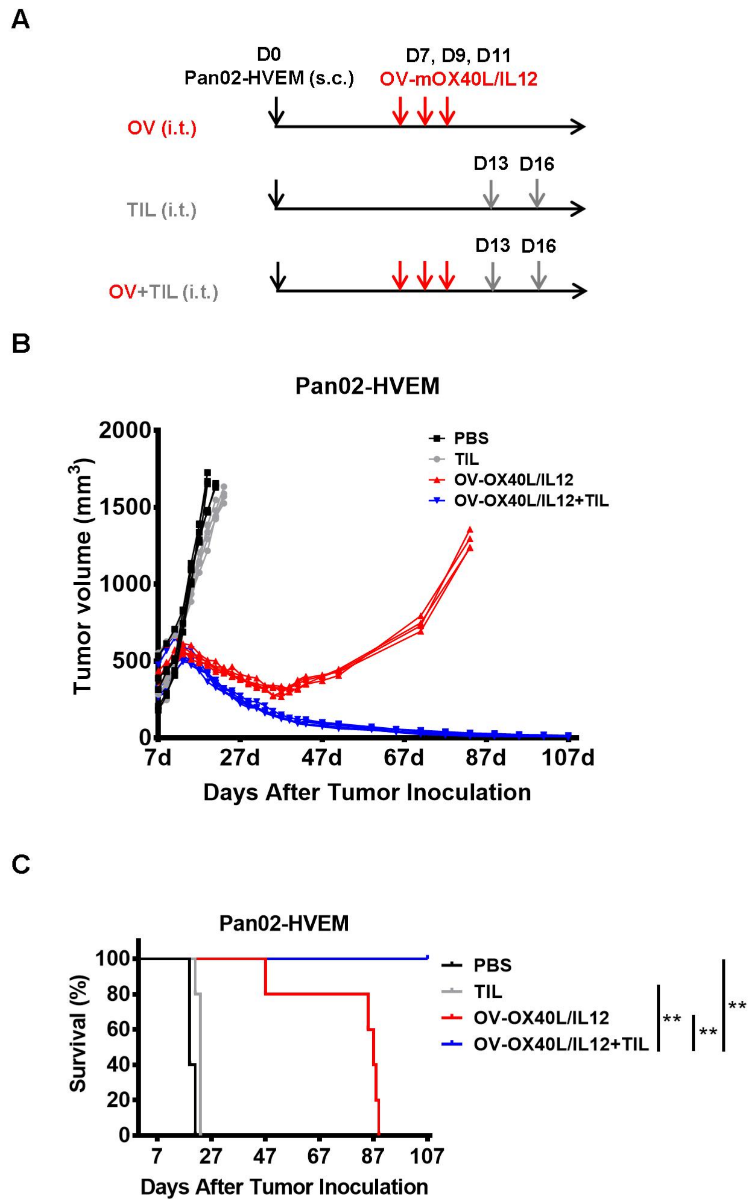

Figure S13. Tumor growth and survival of Pan02-HVEM tumor-bearing mice after treatment.

(A) Schematic of C57BL/6J mice with subcutaneous Pan02-HVEM tumors treated with OV-mOX40L/IL12 and TILs. (B) Mice were subcutaneously injected with Pan02-HVEM cells (1×10^6 cells) on day 0. On days 7, 9 and 11, mice were intratumorally treated with 0 or three doses of 2×10^6 PFUs of OV-mOX40L/IL12 per mouse. On days 13 and 16, the mice were administered intratumoral injections of two doses of TILs (1×10^6 cells). The tumor volume was measured with calipers. Data for each mouse (n = 5 mice per group) are shown. (C) Kaplan–Meier survival curves for the experiment described in (A). The p value indicates the difference between OV-mOX40L/IL12 and OV-mOX40L/IL12+TIL, as determined by using the log rank (Mantel–Cox) test. **$p < 0.01$.

Supplementary Figure 14

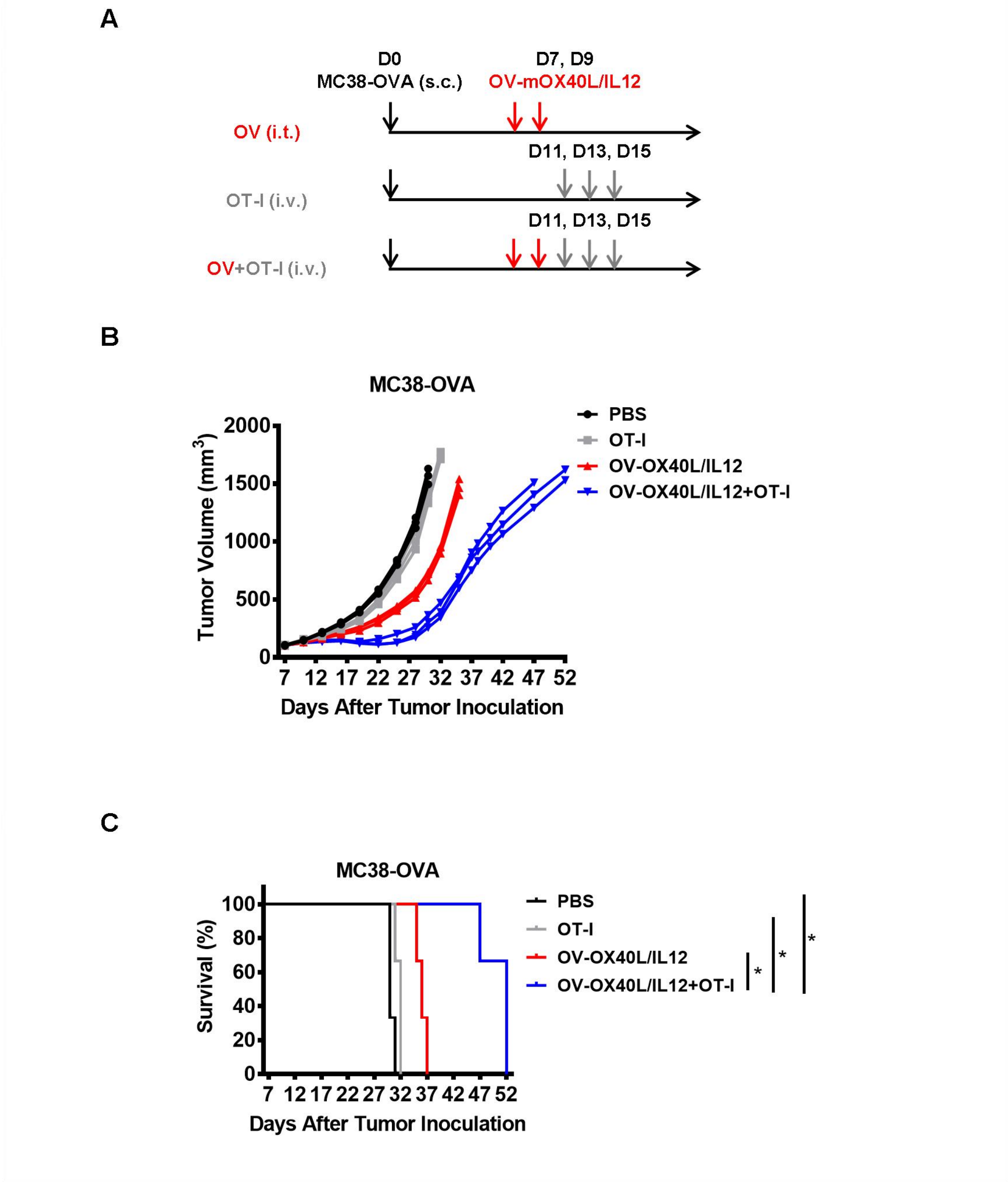

Figure S14. The antitumor effect of intratumoral administration of OV-OX40L/IL12 plus systemic administration of TILs.

(A) Schematic of C57BL/6J mice with subcutaneous MC38-OVA tumors treated with OV-mOX40L/IL12 and OT-I T cells. Mice were injected subcutaneously with MC38-OVA cells on day 0. On days 7 and 9, mice were subcutaneously sham injected or were injected with 2×10^6 PFUs of OV-mOX40L/IL12 per mouse. On days 11, 13 and 15, the mice were injected with PBS or OT-I T cells (1×10^7 cells) via the tail vein. n = 3 mice per group. (B) Growth of transplanted tumors in the MC38-OVA model (n = 3). (C) Kaplan–Meier survival curves for the experiment described in (A). The mean ± SEM are shown. The p value indicates the difference between OV-mOX40L/IL12 and OV-mOX40L/IL12+OT-I, as determined by using the log rank (Mantel–Cox) test. *$p < 0.05$.

Supplementary Figure 15

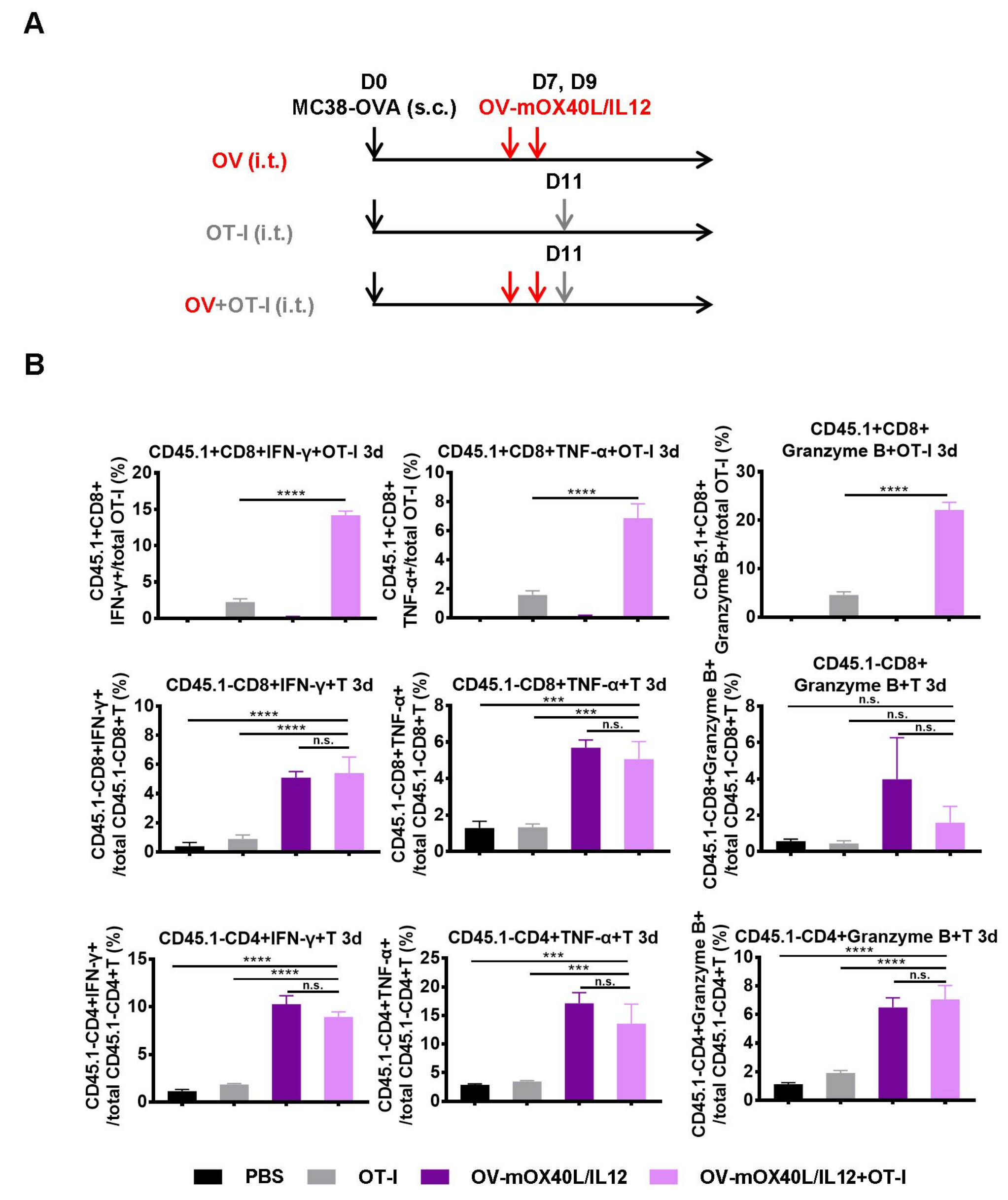

Figure S15. Effect of OV-mOX40L/IL12 on the activation of adoptively transferred and endogenous T cells.

(A) Schematic of C57BL/6J mice subcutaneous MC38-OVA tumors treated with OV-mOX40L/IL12 and OT-I T cells. (B) A single-cell suspension was prepared from tumor tissues 3 days after treatment. TILs were stained and subjected to a flow cytometry analysis to profile different types of immune cells and their activation status. The statistical analysis was determined by one-way ANOVA, followed by Tukey's multiple comparison test analysis. All values are presented as the mean ± SEM. NS, not significant, *$p < 0.05$, **$p < 0.01$, ***$p < 0.001$ and ****$p < 0.0001$.

Supplementary Tables

Table S1. List of anti-human antibodies

Item	Vendor	Catalog number
PE anti-human CD252 (OX40L) Antibody	Biolegend	326307
PE/Dazzle™ 594 anti-human HLA-A, B, C Antibody	Biolegend	311439
FITC anti-human HLA-DR, DP, DQ Antibody	Biolegend	361705
PE anti-human CD80 Antibody	Biolegend	305207
FITC anti-human CD86 Antibody	Biolegend	374203
PE/Dazzle™ 594 anti-human CD274 (PD-L1) Antibody	Biolegend	329731
PE anti-human CD197 (CCR7) Antibody	Biolegend	353203
FITC anti-human CD45RA Antibody	Biolegend	304106
Anti-HLA-DR Antibody, Rabbit Polyclonal	Sino Biological	100586-T08
Anti-HLA A, B, C Antibody, Rabbit Polyclonal	Bioss	bs-10634R
Anti-CD86 Antibody, Rabbit Polyclonal	Bioss	bs-1035R
Anti-IFN gamma Antibody, Rabbit Polyclonal	Bioss	bs-0481R
Anti-CD137 Antibody, Rabbit Polyclonal	Bioss	bs-2449R
Anti-CD134 Antibody, Rabbit Polyclonal	Bioss	bs-2685R

Table S2. List of anti-mouse antibodies

Item	Vendor	Catalog number
FITC anti-mouse CD45 Antibody	Biolegend	103108
PerCP/Cyanine5.5 anti-mouse CD3ε Antibody	Biolegend	100328
Pacific Blue™ anti-mouse CD4 Antibody	Biolegend	100428
Brilliant Violet 510™ anti-mouse CD8a Antibody	Biolegend	100752
Brilliant Violet 605™ anti-mouse CD25 Antibody	Biolegend	102036
PE/Cyanine7 anti-mouse CD335 (NKp46) Antibody	Biolegend	137618
PE/Dazzle™ 594 anti-mouse CD279 (PD-1) Antibody	Biolegend	135228
Alexa Fluor® 647 anti-mouse/rat/human FOXP3 Antibody	Biolegend	320014
PE anti-mouse/human Ki-67 Antibody	Biolegend	151210
PerCP/Cyanine5.5 anti-mouse CD19 Antibody	Biolegend	152406
Pacific Blue™ anti-mouse/human CD11b Antibody	Biolegend	101224
Brilliant Violet 510™ anti-mouse Ly-6C Antibody	Biolegend	128033
Brilliant Violet 605™ anti-mouse Ly-6G Antibody	Biolegend	127639
PE/Cyanine7 anti-mouse F4/80 Antibody	Biolegend	123114
PE/Dazzle™ 594 anti-mouse CD206 (MMR) Antibody	Biolegend	141732
APC anti-mouse I-A/I-E Antibody	Biolegend	107613
PE anti-mouse NOS2 (iNOS) Antibody	Thermo Scientific	12-5920-82
Brilliant Violet 605™ anti-mouse TNF-α Antibody	Biolegend	506329
PE/Dazzle™ 594 anti-mouse IFN-γ Antibody	Biolegend	505846
PE anti-human/mouse granzyme B recombinant Antibody	Biolegend	372208
Pacific Blue™ anti-mouse CD45 Antibody	Biolegend	157211
PE/Cyanine7 anti-mouse CD252 (OX40L) Antibody	Biolegend	108813
PerCP/Cyanine5.5 anti-mouse IL12/IL23 p40 Antibody	Biolegend	505211
PE/Dazzle™ 594 anti-mouse CD86 Antibody	Biolegend	105041
FITC anti-mouse H-2D^b Antibody	Biolegend	111505
Brilliant Violet 605™ anti-mouse CD279 (PD-1) Antibody	Biolegend	135220
PE anti-mouse CD366 (Tim-3) Antibody	Biolegend	134003
PerCP/Cyanine5.5 anti-mouse/human CD45R/B220 Antibody	Biolegend	103235
PerCP/Cyanine5.5 anti-mouse NK-1.1 Antibody	Biolegend	108727
Brilliant Violet 510™ anti-mouse CD11c Antibody	Biolegend	117337
PE/Cyanine7 anti-mouse CD45.1 Antibody	Biolegend	110729
APC anti-mouse/human CD44 Antibody	Biolegend	103012
FITC anti-mouse CD62L Antibody	Biolegend	104406

1. Zhang, H., Sturchler, E., Zhu, J., Nieto, A., Cistrone, P.A., Xie, J., He, L., Yea, K., Jones, T., Turn, R., et al. (2015). Autocrine selection of a GLP-1R G-protein biased agonist with potent antidiabetic effects. Nat. Commun. *6*, 8918.
