# Supplementary material for: An armed oncolytic virus enhances the efficacy of tumor-infiltrating lymphocyte therapy by converting tumors to artificial antigen-presenting cells in situ
Source: Mol Ther. 2022 Jun 17;30(12):3658–76. doi: 10.1016/j.ymthe.2022.06.010 (PMC9734027; doi:10.1016/j.ymthe.2022.06.010)
Supplement: Document S2. Article plus supplemental information [file mmc2.pdf]

Molecular Therapy
Original Article

An armed oncolytic virus enhances the efficacy of tumor-infiltrating lymphocyte therapy by converting tumors to artificial antigen-presenting cells *in situ*

Kai Ye,[1,3,5] Fan Li,[1,3,5] Ruikun Wang,[1,3,5] Tianyi Cen,[1,3,5] Shiyu Liu,[1,3,5] Zhuoqian Zhao,[1,3,5] Ruonan Li,[1,3,5] Lili Xu,[1,3,5] Guanmeng Zhang,[2] Zhaoyuan Xu,[2] Li Deng,[3,4] Lili Li,[3,4] Wei Wang,[6] Alexey Stepanov,[7] Yajuan Wan,[1] Yu Guo,[1,3,5] Yuanke Li,[1,3,5] Yuan Wang,[1,3,5] Yujie Tian,[3,5] Alexander G. Gabibov,[7] Yingbin Yan,[2] and Hongkai Zhang[1,3,5]

[1]State Key Laboratory of Medicinal Chemical Biology and College of Life Sciences, Nankai University, Tianjin 300350, PR China; [2]Department of Oromaxillofacial-Head and Neck Surgery, Tianjin Stomatological Hospital, Tianjin 300041, PR China; [3]CNBG-Nankai University Joint Research and Development Center, Nankai University, Tianjin 300350, PR China; [4]Beijing Institute of Biological Products, Beijing 100176, PR China; [5]Frontiers Science Center for Cell Responses, Nankai University, Tianjin 300350, PR China; [6]Shanghai Institute for Advanced Immunochemical Studies, ShanghaiTech University, Shanghai 201210, PR China; [7]M.M. Shemyakin and Yu.A. Ovchinnikov Institute of Bioorganic Chemistry of the Russian Academy of Sciences, Moscow 117997, Russia

The full potential of tumor-infiltrating lymphocyte (TIL) therapy has been hampered by the inadequate activation and low persistence of TILs, as well as inefficient neoantigen presentation by tumors. We transformed tumor cells into artificial antigen-presenting cells (aAPCs) by infecting them with a herpes simplex virus 1 (HSV-1)-based oncolytic virus encoding OX40L and IL12 (OV-OX40L/IL12) to provide local signals for optimum T cell activation. The infected tumor cells displayed increased expression of antigen-presenting cell-related markers and induced enhanced T cell activation and killing in coculture with TILs. Combining OV-OX40L/IL12 and TIL therapy induced complete tumor regression in patient-derived xenograft and syngeneic mouse tumor models and elicited an antitumor immunological memory. In addition, the combination therapy produced aAPC properties in tumor cells, activated T cells, and reprogrammed macrophages to a more M1-like phenotype in the tumor microenvironment. This combination strategy unleashes the full potential of TIL therapy and warrants further evaluation in clinical studies.

INTRODUCTION

Adoptive cell therapy (ACT) with tumor-infiltrating lymphocytes (TILs) has emerged as a promising therapy for the treatment of solid tumors and has exhibited antitumor efficacy in selected patients with melanoma, ovarian cancer, non-small cell lung cancer (NSCLC), cervical cancer, gastric cancer, and head and neck cancer.[1–4] However, inadequate activation, low persistence of TILs, and poor neoantigen presentation in the tumor microenvironment have prevented TILs from realizing their full potential in most patients.

Classic antigen-presenting cells (APCs), including dendritic cells (DCs), play a central role in initiating the immune response and serve as an ideal tool to enhance antitumor T cell responses.[5] *Ex vivo*-generated monocyte-derived DCs or blood DCs were loaded with tumor antigens in a variety of ways as therapeutic vaccines, and tumor-specific CD8$^+$ T cell responses were detected in vaccinated patients with melanoma, prostate cancer, B cell lymphoma, acute myeloid leukemia, and myeloma.[5,6] Nevertheless, the preparation of DC vaccines is time consuming, costly, and the delivery of the loaded DCs to the tumor microenvironment may be limited, subsequently hindering their potential in boosting T cell-based therapy. Generating APC-like cells in the tumor microenvironment seems to be a plausible alternative. Ideally, artificial APCs (aAPCs) have been engineered to provide the following three signals for T cell activation: signal 1, presents peptide epitopes derived from cancer neoantigens through their major histocompatibility complex (MHC) to T cell receptors (TCR) on T cells; signal 2, provides costimulatory signals by facilitating the binding of CD80/CD86, OX40L, and/or 4-1BBL to their receptors on T cells; and signal 3, secretes cytokines such as interleukin (IL) 12 or IL15 to promote T cell survival, activation, and proliferation.[7,8] APCs derived from K562 cells or other cells lines ectopically expressed a particular human leukocyte antigen (HLA) type, and costimulatory molecules CD80, CD86, 4-1BBL, and cytokine IL15 elicited strong stimulation and expansion of polyclonal or antigen-specific cytotoxic T lymphocytes (CTLs) to the levels required for clinical therapy.[9–12] Sufficient numbers of MART1-specific CTLs were prepared using HLA-A*02:01, CD80-, and CD83-producing aAPCs and infused into patients with melanoma; the adoptively transferred T cells persisted *in vivo*, preferentially localized to tumor sites, and

Received 15 December 2021; accepted 13 June 2022;
https://doi.org/10.1016/j.ymthe.2022.06.010.

Correspondence: Yingbin Yan, Department of Oromaxillofacial-Head and Neck Surgery, Tianjin Stomatological Hospital, Tianjin 300041, PR China.
E-mail: yingbinyan@qq.com
Correspondence: Hongkai Zhang, State Key Laboratory of Medicinal Chemical Biology and College of Life Sciences, Nankai University, Tianjin 300350, PR China.
E-mail: hongkai@nankai.edu.cn

 © 2022 The Author(s).
This is an open access article under the CC BY license (http://creativecommons.org/licenses/by/4.0/).

mediated an antigen-specific immune response characterized by clinically meaningful tumor destruction.[13,14]

Within the tumor microenvironment, tumor cells represent the best candidates for this task because of their abundance in all types of tumors. The concept of transforming tumor cells into APCs has been discussed for more than 30 years.[7,15] Restoring antigen presentation by tumor cells through a variety of treatments may increase the generation of tumor-specific T cells and their capacity to recognize and eradicate tumor cells.[7] In the present study, oncolytic viruses (OVs) armed with cytokines and/or immunostimulatory molecules were selected as tools to transform tumor cells *in situ* because they preferentially infect and replicate in tumor cells.[16] OVs also function as multiplexed immune-modulating platforms by expressing factors such as immune checkpoint inhibitors, tumor antigens, cytokines, and T cell engagers.[17,18] Talimogene laherparepvec (T-Vec), a genetically modified granulocyte-macrophage colony-stimulating factor (GM-CSF)-expressing herpes simplex virus 1 (HSV-1), promoted dendritic cell accumulation and differentiation in tumor foci and increased intratumor T cell infiltration.[19] Dual expression of IL7 and IL12 by an oncolytic vaccinia virus sensitized patients to T cell-based immunotherapies, including checkpoint inhibitors.[20] OVs were modified with CD40 ligand (CD40L) to induce DC maturation and evoke a tumor-specific T cell response.[21–23]

In the present study, we engineered oncolytic HSV-1 to express trimerized OX40L and IL12. After tumor cells were infected with the armed OV and cocultured with TILs, the tumor cells acquired APC-like properties and the tumor-specific T cells were activated and exhibited enhanced tumor-killing activity. Tumor regression was achieved with the combination therapy of OV-OX40L/IL12 and TILs in patient-derived xenograft (PDX) and syngeneic tumor models. The antitumor effect was achieved in both treated and untreated distal tumors, indicating that the combination therapy elicited a systemic immunological response to tumors. In addition, mice exhibiting complete responses were subsequently protected from tumor rechallenge, suggesting that the treated mice developed antitumor immune memory. Flow cytometry analysis of tumor-infiltrating immune cells and immune cell depletion experiments revealed that the armed OV reshaped the tumor microenvironment by increasing the immune activation. Overall, the data showed that OV-OX40L/IL12 transformed tumor cells into aAPCs *in situ* and potentially sensitized patients to TIL therapy, which warrants further evaluation in clinical studies.

RESULTS

Tumor cells infected with armed OVs express transgenes

We generated an armed oncolytic HSV-1 coexpressing the immunestimulatory molecule IL12 and membrane-tethered trimerized human OX40L in the backbone of an ICP34.5 and ICP47 double-deleted oncolytic HSV-1. Recombinant OVs expressing trimerized OX40L, IL12, or GFP were also generated (Figures 1B and S1). Notably, the viral backbone is the same as that of T-Vec and teserpaturev, which have been approved for the treatment of melanoma and glioma.

Primary oral cancer cells from four patients were infected with the parental or armed OVs to assess their ability to infect tumor cells. All the armed OVs efficiently infected the tumor cells and the modification of the OVs did not attenuate their tumoricidal activity (Figure S2). The GFP-encoding OV (OV-GFP) was added to the primary oral cancer tissues, and the susceptibility of the tumor tissues to the OV was confirmed (Figure S3). The cytopathic effect (CPE) induced by the parental and armed OVs in a variety of tumor cells, including glioma, breast cancer, colon cancer, and fibrosarcoma cell lines, was observed (Figure S4). The surface expression of OX40L on the infected cells was confirmed by flow cytometry (Figure 1C) and the activation of cocultured OX40 reporter cells that expressed GFP upon OX40 engagement (Figure 1D). High levels of IL12 secreted from the infected cells were detected by ELISA (Figure 1E). The biological activity of IL12 was confirmed with a luciferase reporter cell line (Figure 1F).

OV-OX40L/IL12 transformed tumor cells to artificial APCs and activated tumor-specific T cells *in vitro*

We evaluated whether the armed OVs successfully transformed tumor cells to aAPCs by monitoring the phenotype in a coculture system. Tumor cells and matching autologous TILs (expanded from the same patient) from four patients with oral cancer were used in the study. The expression of MHC I and MHC II molecules and costimulatory receptors in OV-treated tumor cells was evaluated by using real-time quantitative PCR and flow cytometry. OVs facilitated the display of HLA-A/B/C and HLA-DR/DP/DQ on the surface of tumor cells. All armed OVs, including OV-OX40L, OV-IL12, and OV-OX40L/IL12, increased the expression of the costimulatory receptors CD80 and CD86 on tumor cells, with the strongest upregulation observed in cells infected with OV-OX40L/IL12, followed by OV-OX40L and OV-IL12. The programmed death-ligand 1 (PD-L1) expression on tumor cells was also increased when tumor cells were infected with OV-OX40L, OV-IL12, or OV-OX40L/IL12 and cocultured with TILs, providing the rationale for the combination therapy of these OVs with programmed cell death protein 1 (PD-1) inhibitors (Figures 2A and 2B).

In the coculture, tumor cells infected with OV-Δ34.5 had no effect on inducing TIL expansion in the short-term coculture experiment (Figure 2C), while OV-OX40L-, OV-IL12-, or OV-OX40L/IL12-infected tumor cells stimulated T cell proliferation (Figure 2C).

Interferon gamma (IFN-γ) secretion was examined by IFN-γ enzyme-linked immunospot (ELISpot) and ELISA as an indicator of T cell activation. TILs cocultured with OV-OX40L/IL12-infected tumor cells exhibited the most significant increase in the number of IFN-γ-secreting cells compared with TILs cocultured with mock- or OV-Δ34.5-infected tumor cells (Figures 2D and 2E). The finding was confirmed by IFN-γ concentration in the culture supernatant, showing that cocultures with tumor cells infected with OV-OX40L/IL12 increased IFN-γ production to the highest level (Figures 2F and S5A).

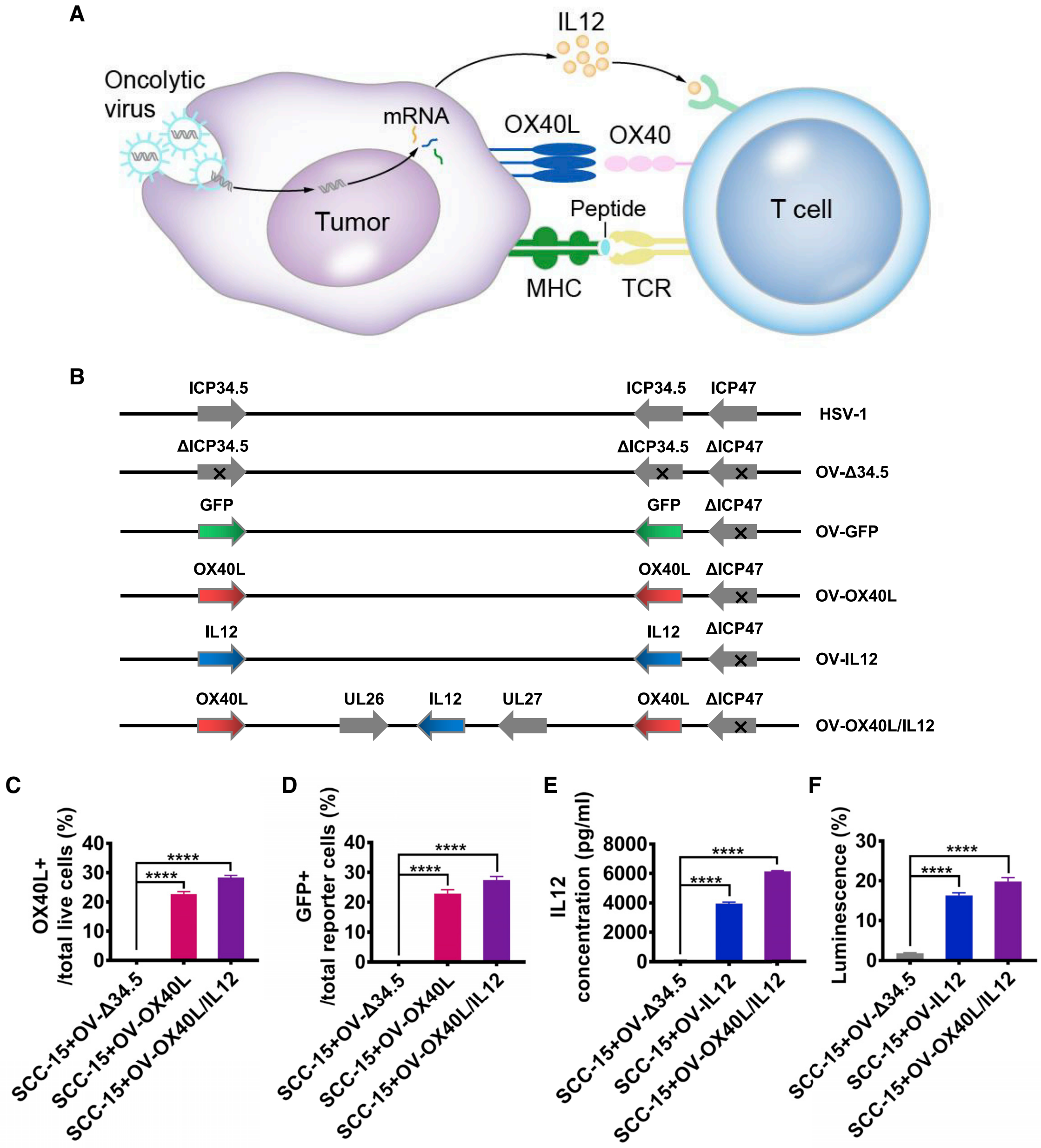

Figure 1. Generation of engineered OVs encoding trimerized OX40L and/or IL12

(A) Diagram showing the transformation of tumor cells into aAPCs to activate tumor-specific T cells through OV-mediated expression of OX40L and IL12 on tumor cells. (B) Schematic representation of HSV-1-based OVs encoding OX40L, IL12, or both proteins. The expression of functional OX40L by the infected SCC-15 tumor cells was analyzed (C) by using flow cytometry and (D) by monitoring the activation of cocultured OX40 reporter cells that expressed GFP upon OX40 signaling (n = 3). (E) Secretion of IL12 from the infected cells was detected by using an ELISA (n = 3). (F) The expression of functional IL12 by the infected SCC-15 tumor cells was analyzed by monitoring the activation of cocultured IL12 luciferase reporter cells (n = 3). The statistical analysis was determined by one-way ANOVA, followed by Tukey's multiple comparison test analysis. All values are presented as the mean ± SEM. ****$p < 0.0001$.

We performed single-cell TCR sequencing (scTCR-seq) and single-cell RNA sequencing (scRNA-seq) to comprehensively analyze the TCR repertoire and transcriptome of TILs cocultured with mock- or OV-OX40L/IL12-infected tumor cells. Both groups possessed a similar level of diversity among the TCRs. No significant difference in the CDR3 length, T cell receptor alpha variable (TRAV), and T cell receptor beta variable (TRBV) germline usage was observed compared with the TCRs of the TILs without coculture

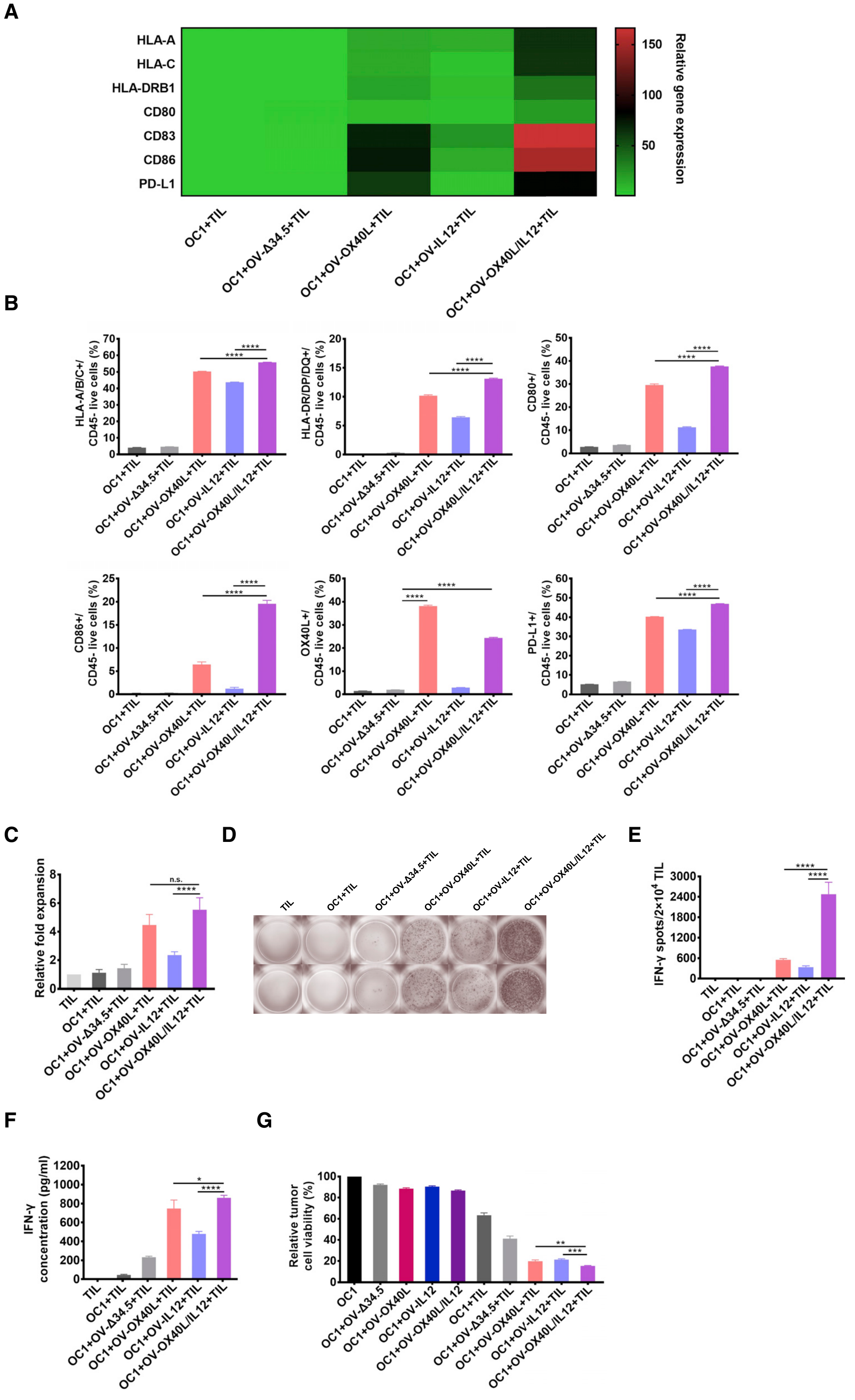

(legend on next page)

(Figures S6A and S6B). Among the 10 most prevalent clonotypes, the percentage of IFN-γ-positive T cells (which are considered activated T cells) in the OV-OX40L/IL12 group was 2- to 3-fold higher than that in the mock-infected group (Figure S6C). Molecular deconvolution of the TCR clonotypes and the corresponding activation status corroborated the effectiveness of the armed OV.

Tumor cell viability was quantified in the cocultures to assess the cytotoxicity of TILs. After tumor cells were infected with the low dose of the unarmed OV-Δ34.5 or the armed OVs, the viability decreased by 10%–20% due to the direct oncolytic activity of the OVs. Autologous TILs expanded from the same patients decreased tumor cell viability by 30%–40%, showing that TIL therapy alone produced modest tumoricidal activity. In the presence of TILs and a low dose of the armed OVs, significantly increased killing of the tumor cells was observed compared with TIL and parental OV-Δ34.5 or OV in the absence of TILs (Figures 2G and S5B), suggesting that the killing of tumor cells was mediated by TILs and enhanced by the armed OV encoding OX40L and IL12.

Combination immunotherapy with OV-OX40L/IL12 and autologous TILs led to tumor regression in PDX models

The *in vivo* antitumor activity of the OX40L- and IL12-encoding OV and TIL combination therapy was evaluated in two oral cancer PDX models. The PDX mouse models were administered a single intratumor injection of OV-Δ34.5 or OV-OX40L/IL12, followed by intratumor delivery of autologous TILs 2 days later (Figure 3A). In the first PDX model (OC1 PDX), TIL therapy alone or in combination with unarmed OV-Δ34.5 resulted in slower tumor growth than that in the control group, with eventual tumor progression. The combination therapy of OV-OX40L/IL12 and TILs induced complete or nearly complete tumor regression in all animals, with no tumor regrowth observed 55 days after TIL treatment (Figure 3B). In a separate pharmacodynamic (PD) study, mice were euthanized at different time points to study the effect on the tumor microenvironment. One week after treatment, combination therapy with OV-OX40L/IL12 and TILs significantly increased the intratumor IFN-γ level compared with that in the other groups (Figure 3C). Immunohistochemical (IHC) staining revealed that OV-OX40L/IL12 and TIL combination therapy resulted in stronger staining for HLA-A/B/C, HLA-DR, CD86, CD134, CD137, and IFN-γ compared with the other groups at 14 and 21 days after treatment (Figures 3D and S7).

The antitumor efficacy of the combination therapy of OV-OX40L/IL12 and TILs was confirmed in the second oral cancer PDX model (OC4 PDX), with all mice receiving the combination therapy of OV-OX40L/IL12 and TILs displaying progressive tumor shrinkage, while all animals in the other groups showed tumor progression 4 weeks after treatment (Figure S8).

Combination therapy with OV-mOX40L/IL12 and TILs led to marked tumor regression and long-term immune memory in immunocompetent murine tumor models

Next, syngeneic MC38 or Pan02 models were established to study the systemic immunological effects and immune memory. For this purpose, we generated an OV encoding murine IL12 and trimerized OX40L and validated their expression (Figures S9A and S9B). In addition, herpesvirus entry mediator (HVEM) was introduced into Pan02 tumor cells to ensure that the cells were susceptible to HSV-1 infection. $CD8^+$ T cells were isolated from subcutaneously engrafted MC38 or Pan02-HVEM tumors and expanded for adoptive transfer.

The antitumor activity of this combination therapy was tested in a syngeneic tumor model established using C57BL/6J mice bearing subcutaneous implanted murine colorectal MC38 tumors. Single treatment with OV-Δ34.5 or TILs or their combination marginally delayed tumor growth, with continued tumor progression observed in all animals. The combination of OV-Δ34.5 and MC38 TILs exhibited additive antitumor effect. OV-mOX40L/IL12 treatment resulted in marked tumor growth inhibition, but no mice were cured of tumors. The combination of OV-mOX40L/IL12 and MC38 TILs led to complete regression in all treated mice, and animals remained tumor free until 180 days after treatment. The anti-PD-1 antagonist antibody only marginally increased the efficacy of OV-mOX40L/IL12 in the MC38 syngeneic mouse model (Figures 4B and 4C).

The *in vivo* efficacy was also tested in a murine pancreatic cancer Pan02 model. Pan02 is a very aggressive syngeneic tumor model and has been shown to be refractory to checkpoint inhibition. In this model, Pan02-HVEM TILs, OV-Δ34.5, and their combination did not show significant antitumor efficacy. The combination of OV-mOX40L/IL12 and a PD-1 antibody with or without Pan02-HVEM TILs resulted in significant inhibition of tumor growth. Tumors completely regressed in three of six mice (Figures S9C and S9D). Therefore, three doses of OV-mOX40L/OV-IL12 and two doses of TIL were administered to the Pan02-HVEM-bearing mice. Strikingly, this new regimen led to a 100% complete tumor rejection in mice (Figures S13A–S13C).

All treatments were well tolerated, with no significant weight loss or other signs of distress observed. We also assessed whether intratumor

Figure 2. OV-OX40L/IL12 transformed tumor cells into aAPCs to activate tumor-specific T cells

Primary cancer cells from a patient with oral cancer numbered OC1 were infected with the indicated OV and cocultured with TILs expanded from the same patients. The expression of APC-related genes (HLA-A/B/C, HLA-DP/DR/DQ, CD80, CD86, and OX40L) by tumor cells (n = 3) was assessed using RT–PCR (A) and flow cytometry (B). (C) The number of TILs in the coculture assay was counted and normalized to the number of TILs cultured without OV-infected tumor cells (n = 3). (D) After coculture, the suspension cells (mostly TILs) were added to precoated ELISpot wells to detect IFN-γ-secreting cells. (E) The spot number in the ELISpot experiment was counted (n = 3). (F) IFN-γ levels in the coculture supernatant were determined by using an ELISA (n = 3). (G) The viability of adherent (tumor) cells in the coculture assay was assessed by using the MTT assay (n = 3). The statistical analysis was determined by one-way ANOVA, followed by Tukey's multiple comparison test analysis. All values are presented as the mean ± SEM. NS, not significant, *p < 0.05, **p < 0.01, ***p < 0.001, and ****p < 0.0001.

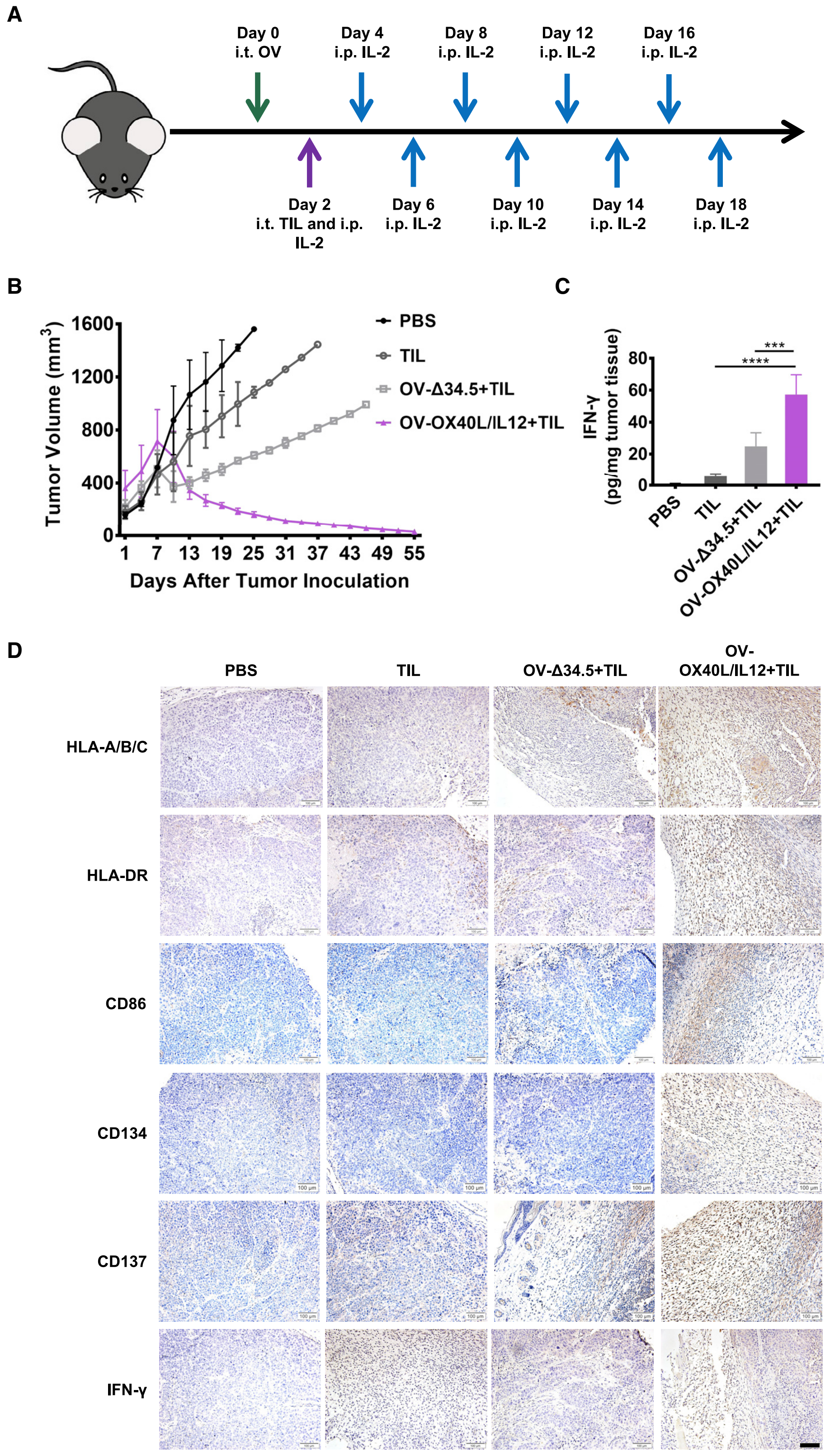

(legend on next page)

delivery of OV resulted in the infection of nontumor tissues. The OV was not detected in the spleen, liver, or lung of mice injected intratumorally with the OV alone or OV combined with TILs. These data suggested low safety risks with the combination therapy.

Rechallenge studies were performed to evaluate immune memory. Mice cured of MC38 tumors were resistant to rechallenge with MC38 tumors on the untreated side, whereas all age-matched tumor-naive control mice developed tumors (Figures 4D and 4E). This protective effect against tumor rechallenge was also observed in the Pan02-HVEM model (Figures S9E and S9F), implying the development of long-term immune memory.

In the bilateral MC38 tumor-bearing mice, the OV-OX40L/IL12 and TILs were injected into one side of tumor to evaluate the systemic effect. Robust tumor inhibition efficacy in both treated and contralateral tumors was noted with both OV therapy alone and the combination therapy. Remarkably, the combination therapy led to near-complete control of both treated and distal untreated tumors (Figures 4F and 4G), indicating that it elicited a systemic antitumor effect. The results support the potential of this approach in the treatment of metastatic disease.

The effect of OV-mOX40L/IL12 on intravenously administered T cells were evaluated (Figure S14A). The combination of intratumor administration of OV-mOX40L/IL12 with intravenously infused ovalbumin (OVA)-specific OT-I T cells significantly inhibited tumor growth and prolonged the overall survival of mice compared with the adoptive transfer of OT-I T cells alone (Figures S14B and S14C), supporting the hypothesis that OV-OX40L/IL12 can enhance the activity of systemically administered TILs.

$CD8^+$ CTL cells were important for the inhibition of tumor growth induced by the combination therapy consisting of the OV and adoptive transfer of TILs

In vivo depletion of $CD4^+$ T cells, $CD8^+$ T cells, and natural killer (NK) cells was performed to determine the role of immune cell subsets in the antitumor effect. Depletion of $NK1.1^+$ cells or $CD8^+$ T cells in mice treated with OV-mOX40L/IL12 restored tumor growth. In contrast, no significant difference in tumor growth was observed in $CD4^+$ T cell-depleted mice compared with the undepleted mice. In the mice treated with the combination therapy, only the depletion of $CD8^+$ cells abrogated the antitumor effect of the treatment, while the depletion of NK cells exerted a minor effect and depletion of $CD4^+$ T cells had a minimal effect on tumor inhibition by the combination therapy (Figures 4H–4J). Thus, our data indicated that $CD8^+$ T cells play an indispensable role in the inhibition of tumor growth in mice treated with OV-mOX40L/IL12 or the armed OV and TILs combination therapy.

Local treatment reinvigorated tumor-infiltrating immune cells

We analyzed the tumor cells and tumor-infiltrating immune cells in both the MC38 and Pan02-HVEM tumor models by using flow cytometry on days 3 and 7 post treatment to identify the mechanism underlying the synergistic effect of TILs and OV-mOX40L/IL12 (Table S2).

Treatment with OV-mOX40L/IL12 alone or in combination with autologous TIL therapy increased the expression of MHC I, MHC II, costimulatory ligands (CD86 and OX40L), and IL12 in a substantial percentage of the tumor cells, indicating the successful conversion of tumor cells into APC-like cells (Figures 5A, S10A, S11C, and S11E). Moreover, both OV-mOX40L/IL12 alone and the combination therapy increased the proportion of mature DCs in tumors and tumor-draining lymph nodes (Figures 5B and S10E).

The administration of OV-mOX40L/IL12 alone or in combination with TILs led to increased frequencies of $CD8^+$ T cells and NK cells compared with the control group or TIL alone group. After treatment with OV-mOX40L/IL12 or the combination therapy, the expression of IFN-γ, TNF-α, and granzyme B in $CD8^+$ T cells and NK cells was upregulated, while the percentage of PD-1-high and TIM-3-positive exhausted T cells was reduced, with the greatest effect observed in the combination therapy group (Figures 5B, S10B, S11D, and S11F).

To distinguish the effects on endogenous T cells and adoptively transferred T cell populations, $CD45.1^+$ OT-I T cells were adoptively transferred into $CD45.2^+$ C57BL/6 mice bearing MC38-OVA tumors (Figure S15). OV-mOX40L/IL12 treatment alone and in combination with TIL therapy promoted the activation of both endogenous T cells and adoptively transferred OT-I T cells (Figure S15).

The OV alone or in combination with TILs also reprogrammed the myeloid cells in the tumor microenvironment toward an increasingly immune-active state. The number of antitumor or M1 macrophages (inducible nitric oxide synthase, $iNOS^+$) increased, whereas the number of protumor or M2 macrophages ($CD206^+$) decreased substantially (Figures 5B, S10B, S11D, and S11F).

Splenocytes were also analyzed to assess the systemic immune landscape outside the tumor microenvironment. The frequencies of

Figure 3. Antitumor efficacy of the combination therapy with OV-OX40L/IL12 and TILs in the first PDX tumor model (OC1 PDX model)

(A) Schematic diagram of the PDX model treated with OV-OX40L/IL12 and TILs expanded from the same patient numbered OC1. NSG mice were subcutaneously implanted with patient-derived tumor tissues, and the established tumors were intratumorally injected with the indicated OV followed by intratumoral injection of *ex vivo* expanded TILs from the same patient. Tumor growth was measured every other day (n = 4). The average tumor volumes (B) are shown. Arrows indicate the OV, TIL, and IL2 injections. (C) Intratumor IFN-γ levels were determined by using an ELISA assay. (D) Representative images of IHC staining for HLA-A/B/C, HLA-DR, CD86, CD134, CD137, and IFN-γ in tumor tissue sections at 14 days after treatment (n = 4). Scale bars, 100 μm. The statistical analysis was determined by one-way ANOVA, followed by Tukey's multiple comparison test analysis. All values are presented as the mean ± SEM. ***$p < 0.001$, ****$p < 0.0001$.

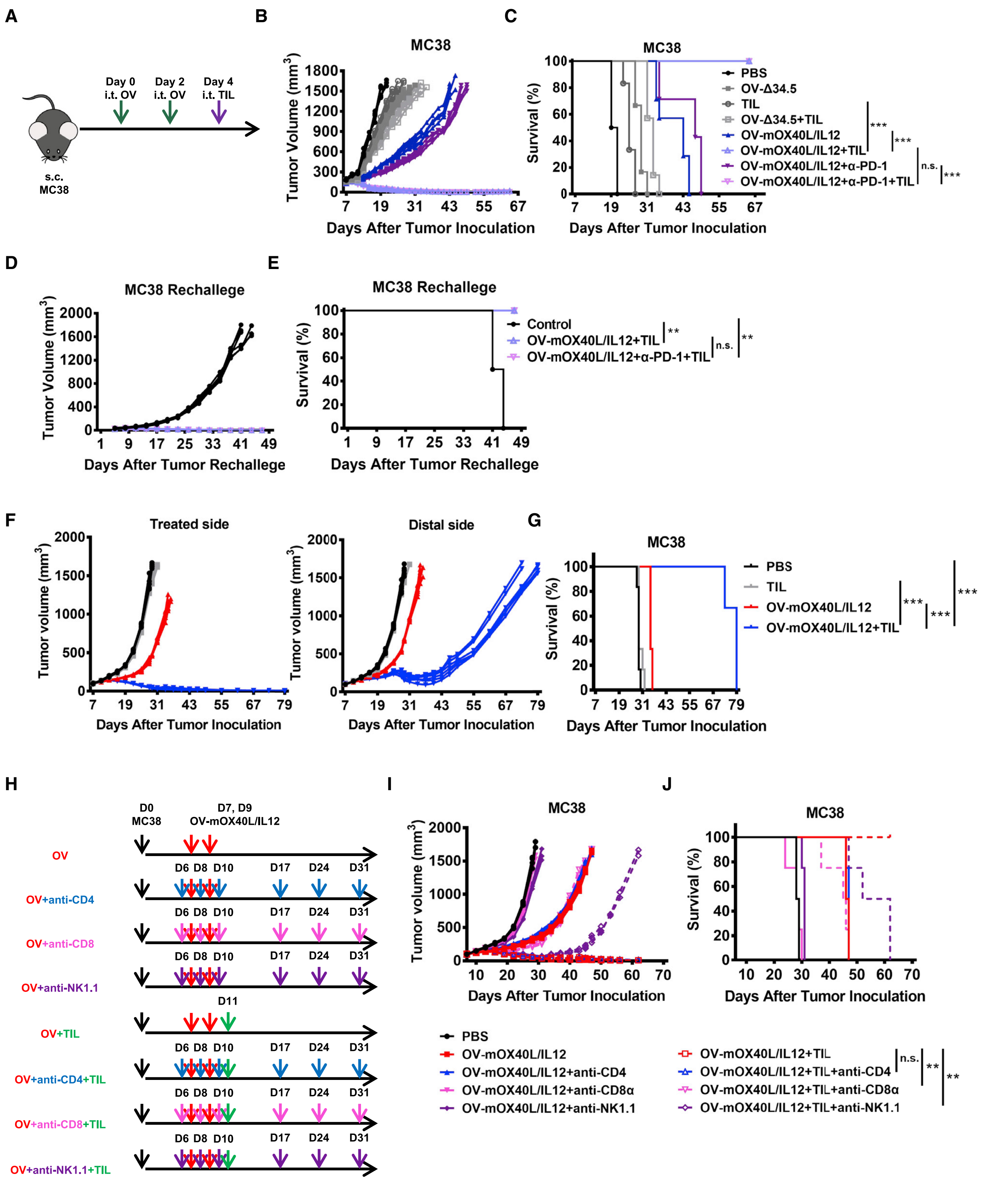

(legend on next page)

conventional $CD4^+$ T cells and $CD8^+$ T cells did not change; however, the frequency of NK cells was increased and that of regulatory T cells (Tregs) was decreased upon treatment with OV alone or in combination with TIL therapy. More T cells and NK cells were activated, and the macrophages functionally switched toward the M1-like phenotype, not the M2-like phenotype, upon treatment with the OV alone or in combination with TILs (Figures S10C, S10D, S11A, and S11B).

We profiled the cytokines in serum on day 3 (Figure 6) and day 7 (Figure S12) after the last treatment using the Bio-Plex Pro Mouse Cytokine 23-plex immunoassay. IL12 levels were significantly increased on both day 3 and day 7 after treatment with the OV alone or in combination with TILs, potentially due to the secretion of the cytokines following the expression of the transgene. The levels of the pharmacodynamic response markers IFN-γ, IL2, and TNF-α were increased on both day 3 and day 7. These cytokines were shown to be associated with type 1 cellular immunity and were critically required for effective priming, proliferation, and recruitment of the tumor-specific T cell response. Notably, IL9 is one of the cytokines whose levels are highly increased in serum upon treatment with the OV alone or in combination with TILs. IL9 is the main effector cytokine of Th9 cells, which have a stronger and longer antitumor ability than T helper (Th) 1 and Th17 cells. In contrast, we observed a significant decrease in IL5, IL10, IL13, and IL6 levels, which are typically associated with tumor-promoting type 2 immune responses, which are known to support tumor growth, metastatic dissemination, and tumor evasion of immune surveillance. These data suggested that local delivery of either OV-mOX40L/IL12 alone or in combination with TILs elicited a systemic immune response.

Tumor-derived aAPCs primed naive T cell responses

Mature DCs are known to activate naive T cells in addition to antigen-experienced TILs. To evaluate whether tumor-derived aAPCs are able to do so, OV-infected MC38-OVA tumor cells were cocultured with naive T cells isolated from OT-I or OT-II mice, and analyzed by flow cytometry (Figures 7A and 7C) and ELISpot (Figures 7B and 7D).

OV-OX40L/IL12-infected tumor cells promoted the development of effector functions, including cytolytic activity and IFN-γ production, in naive T cells. After coculture with OV-OX40L/IL12-infected tumor cells, naive OT-I and OT-II T cells differentiated into memory and effector T cells (Figure 7). Thus, OV-OX40L/IL12-infected cells primed naive T cell responses.

The OV-infected cancer cells efficiently reactivated TILs, and TNF-α and IFN-γ (Figures 6 and S12), which are known to upregulate MHC and costimulatory molecules on the surface of tumor cells and may synergize with OV-OX40L/IL12 to prime a naive T cell. Therefore, TNF-α and IFN-γ were added to the coculture of virus-infected tumors and naive T cells. As expected, the addition of TNF-α and IFN-γ exerted a synergistic effect with OV-OX40L/IL12 on promoting differentiation of naive cells into memory T cells and effector T cells, as well as their effector function (Figure 7). The data suggest that the engineered tumor cells are APC-like cells that activate naive T cells, especially when TILs are present in the tumor microenvironment.

DISCUSSION

Cell therapies such as chimeric antigen receptor T (CAR-T) cell and TIL therapy have been in the spotlight in the field of cancer immunotherapy. Nevertheless, the clinical outcomes of immune cell therapy have been largely disappointing in solid tumors, which is partially attributed to the heterogeneous mutations in tumors and inadequate T cell activation and proliferation in the immunosuppressive tumor microenvironment. A low dose of the armed oncolytic virus OV-OX40L/IL12 was injected into tumors to convert tumor cells into artificial APCs *in situ* prior to the adoptive transfer of TILs as a method to augment tumor-specific T cell expansion and antitumor activities.

When suboptimally primed, T cells become hyporesponsive or anergic and show low proliferation and effector functions in response to antigens.[14] T cells must be initially primed by APCs within an optimal immunologic context to avoid this anergic state induced by suboptimal priming. Originally, autologous APCs, such as DCs loaded with tumor antigens *ex vivo* and administered as vaccines, were found to elicit antitumor immunity and tumor regression in clinical trials.[24] The preparation of autologous DCs or monocyte-derived DCs remains a cumbersome, time-consuming task, and delivery to the tumor microenvironment may be limited. Furthermore, the quantity and quality of prepared autologous APC varies between individuals,

Figure 4. Combination therapy with OV-mOX40L/IL12 and TILs led to marked tumor regression in immunocompetent murine tumor models

(A) Schematic of C57BL/6J mice with subcutaneously implanted tumors treated with the OV and TILs. Immunocompetent C57BL/6J mice (n = 7) were implanted subcutaneously with MC38 cells, and the established tumors were intratumorally injected with the indicated OV followed by intratumoral adoptive transfer of the corresponding TILs. Tumor growth was measured every other day. (B) Tumor volumes are shown as the mean ± SEM. (C) The survival curve of MC38 tumor-bearing mice (n = 7) was plotted using the Kaplan-Meier analysis, and the log rank test indicated a significant difference in the survival curves between the OV-mOX40L/IL12, PD-1 antagonist antibody and TIL combination therapy groups and the OV-Δ34.5 + TIL group. NS, not significant, $***p < 0.001$. Mice that were previously cured of MC38 tumors with the combination therapy and age-matched treatment-naive mice (n = 6) were subcutaneously inoculated with MC38 cells. Tumor growth in individual mice (D) and the survival curve (E) are shown. NS, not significant, $**p < 0.01$. (F) Mice were subcutaneously inoculated with MC38 cells in both flanks. After the establishment of tumors, PBS and OV-mOX40L/IL12 were injected into the tumors on one side (treated side) on days 7 and 9. On day 11, mice were intratumorally administered with TILs. Growth of the injected tumors and the distant tumors was measured. Data from each mouse (n = 6 mice per group) are shown. (G) Kaplan-Meier survival curves from the experiment. $***p < 0.001$. (H) Antibody-mediated immune cell depletion was then performed individually for $CD4^+$ T cells, NK cells, and $CD8^+$ T cells via i.p. injection according to the depicted schedule. (I) Tumor growth was monitored every 3 days using electric calipers to measure the tumor size (n = 4). (J) Kaplan-Meier survival curves from the experiment described in (H). The p value indicates the difference between tumor-bearing mice receiving and not receiving CD4, CD8, or NK depletion antibodies, as determined by using the log rank (Mantel-Cox) test. NS, not significant, $**p < 0.01$.

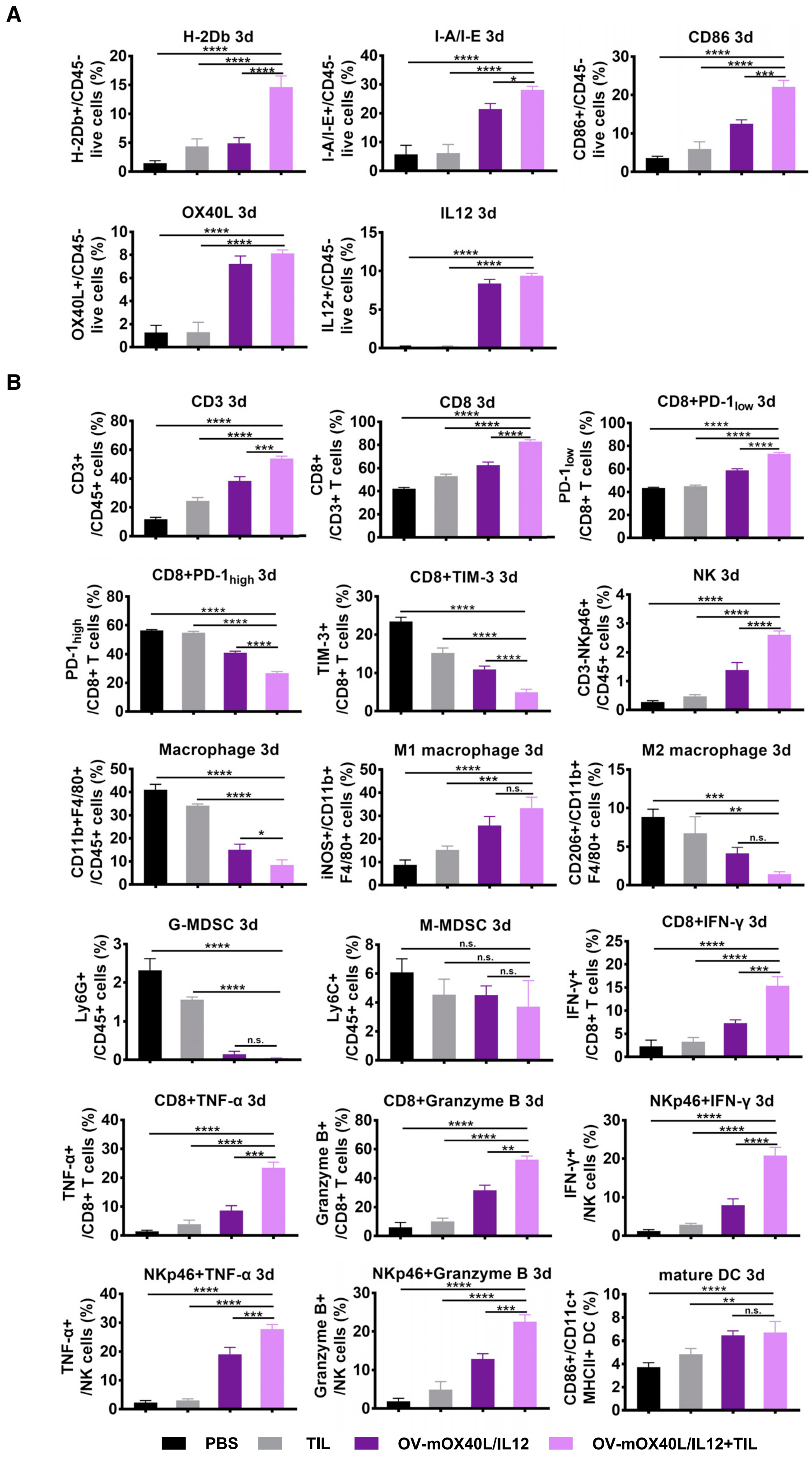

(legend on next page)

and this treatment is only accessible to patients living close to one of the production centers.[14] Additional tumor-intrinsic factors also contribute to the limited success of DC vaccines.[25] Researchers have not determined whether the DC vaccine will still produce cytokines and upregulate costimulatory molecules in the tumor microenvironment. These issues inherent to DC vaccines have led to efforts to develop strategies that provide optimal signals to T cells.

Artificial APCs (aAPCs) have been developed as a promising alternative to overcome the limitations and disadvantages of autologous APCs and DC-based vaccines.[26] Cellular aAPC systems are developed from cells that are genetically engineered to introduce molecules required for immune synapse formation. More recently, acellular aAPCs based on synthetic platforms showed the potential to engage with and activate T cells by conjugating the biological signals onto the surface of microparticles or nanoparticles. Zhang et al. produced a nanoparticle with cell membrane derived from cancer cells engineered to express CD80, a costimulatory signal.[27] Liu et al. developed a nanovaccine derived from DCs that genetically expressed a specific peptide-MHC complex, anti-PD1 antibody, and B7 costimulatory molecules to integrate antigen self-presentation and immunosuppression reversal.[28]

Rosa et al. showed that ectopic expression of a combination of transcription factors is useful to direct the reprogramming of fibroblasts into antigen-presenting DCs.[29] This reprogramming technology inspires the use of gene therapy to reprogram cancer cells into APCs *in situ*. Conversion of tumor cells to aAPCs has four unique advantages. First, this approach transforms one of the most important cellular sources of immune suppression into immune activation. Second, it resolves the most critical issues of natural or adoptively transferred professional APCs, including homing to tumors and becoming dysfunctional in the tumor microenvironment.[15,30] Third, it enhances cancer cell presentation of their own antigens to trigger the proliferation and activation of tumor-specific TILs and does not rely on the identification of antigen epitopes, which has been challenging. Last, the OV is a universal therapy that can be administered to different patients to induce personalized immune responses. We expected that our method will harness the full potential of TIL therapy and other T cell-mediated therapies, as suggested by the results of the TIL combination therapy in PDX and syngeneic mouse models.

MHC I and MHC II molecules are required for the presentation of neoantigens and elicitation of CTLs and Th cells, respectively.[31] The downregulation or absence of MHC molecules from the tumor cell surface leads to the escape of these tumors from immunosurveillance. Mouse tumor cells transfected with MHC I or MHC II genes are rejected by mice.[15] MHC I molecules are upregulated on cells upon infection with the OV.[32] Notably, HSV-1 encodes ICP47 to disrupt MHC I antigen processing and presentation;[33] thus, ICP47 was deleted in our oncolytic virus backbone. Although MHC II is usually not expressed by solid tumors, its expression in tumor cells can be induced by IFN-γ,[34] forming a positive feedback loop between T cell activation and MHC II induction in tumor cells. Moreover, OVs induced autophagy in cancer cells to stimulate antigen processing for MHC class II but also facilitated antigen cross-priming to generate tumor-associated antigen-specific CD8$^+$ T cells.[35]

The dominant second signal is transmitted when B7-1/CD80 and B7-2/CD86 on the surface of APCs engage CD28 on T cells.[7] Tumors usually do not trigger costimulatory signals that are necessary for full activation of T cells. Different mouse tumor cells were transfected or transduced with the costimulatory ligand B7 or other costimulatory ligands, and these cells were rejected by mice. PsiOxus and DNAtrix oncolytic adenoviruses were armed with full-length CD80 and OX40L to induce a vigorous T cell response.[30] Nevertheless, upregulation of CD80 and CD86 on tumor cells was observed after treatment with OV-OX40L/IL12 *ex vivo* or *in vivo* in our study, suggesting that including some costimulatory ligands is sufficient to jump-start a network of costimulatory signals.

Although the OX40L monotransgene OV seems to be almost as effective as the double transgene OV in modulating T cell proliferation in the 48-h cell coculture assay, the double transgene virus induced significantly more IFN-γ-secreting cells than the monotransgene OV in the ELISpot assay (Figures 2C–2E). This is consistent with previous *in vitro* studies using aAPCs and *in vivo* studies using a peptide immunization models, where it was revealed that the development of optimum effector function, and programming for CD8$^+$ T cell memory development, requires IL12 or type I IFN.[36–39] In the absence of IL12, stimulation with antigen in the presence of B7-1 costimulatory signal results in T cell proliferation but suboptimal effector functions, and the cells become anergic. In the presence of IL12, cells displayed comparable proliferation but strong effector functions and productive memory.[36,40] Nevertheless, clinical applications of IL12 have been hampered by severe adverse events observed upon its systemic administration. Intratumoral delivery of the IL12 gene using mRNA, DNA, or an OV overcame toxicity issues and showed promising results in clinical trials.[41–43] The codelivery of OX40L and IL12 has not been previously reported, and our data revealed that OVs armed with both OX40L and IL12 induced robust antitumor activity and established antitumor immune memory.

Transforming tumor cells into aAPCs through OVs encoding OX40L and IL12 represents a unique strategy to enhance T cell-based immunotherapy. A variety of armed OVs have been studied

Figure 5. Analysis of tumor cells and tumor-infiltrating immune cells after the tumor-bearing mice were treated with the OV and TILs

Mice (n = 3) were subcutaneously implanted with MC38 cells and treated as indicated. A single-cell suspension was prepared from tumor tissue 3 days after treatment. Tumor cells (A) and tumor-infiltrating immune cells (B) were stained and subjected to a flow cytometry analysis of the expression of APC-related molecules on the tumor cells (A) and profiling of different types of immune cells and their activation status (B). The statistical analysis was determined by one-way ANOVA, followed by Tukey's multiple comparison test analysis. All values are presented as the mean ± SEM. NS, not significant, *$p < 0.05$, **$p < 0.01$, ***$p < 0.001$, and ****$p < 0.0001$.

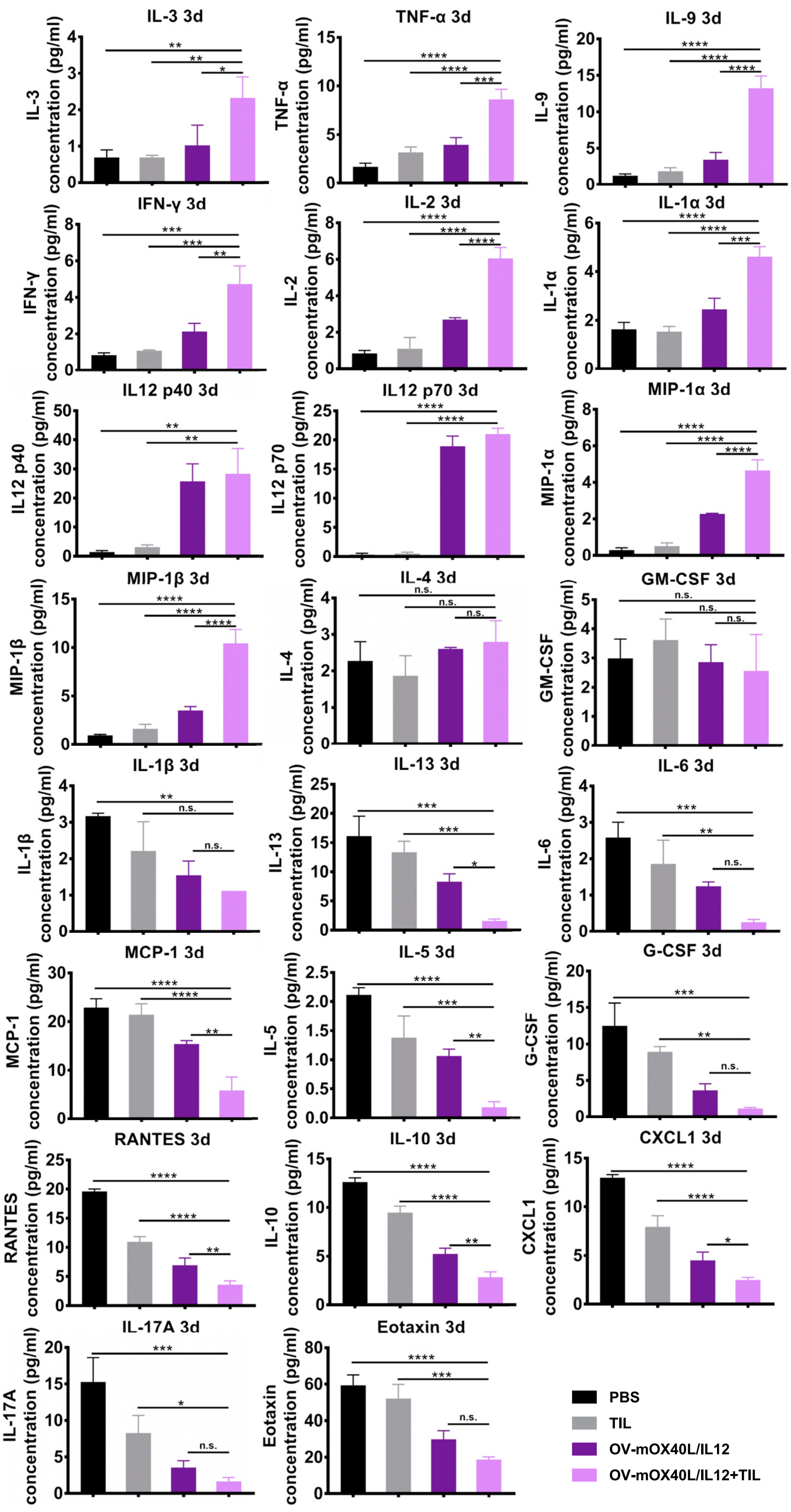

Figure 6. Profiles of cytokines and chemokines in serum samples from treated mice

Mice (n = 3) were subcutaneously implanted with MC38 cells and treated as indicated. Cytokine concentrations in serum collected at 3 days post treatment were determined using a Bio-Plex Mouse Cytokine 23-Plex Array. The statistical analysis was determined by one-way ANOVA, followed by Tukey's multiple comparison test analysis. All values are presented as the mean ± SEM. NS, not significant, *p < 0.05, **p < 0.01, ***p < 0.001, and ****p < 0.0001.

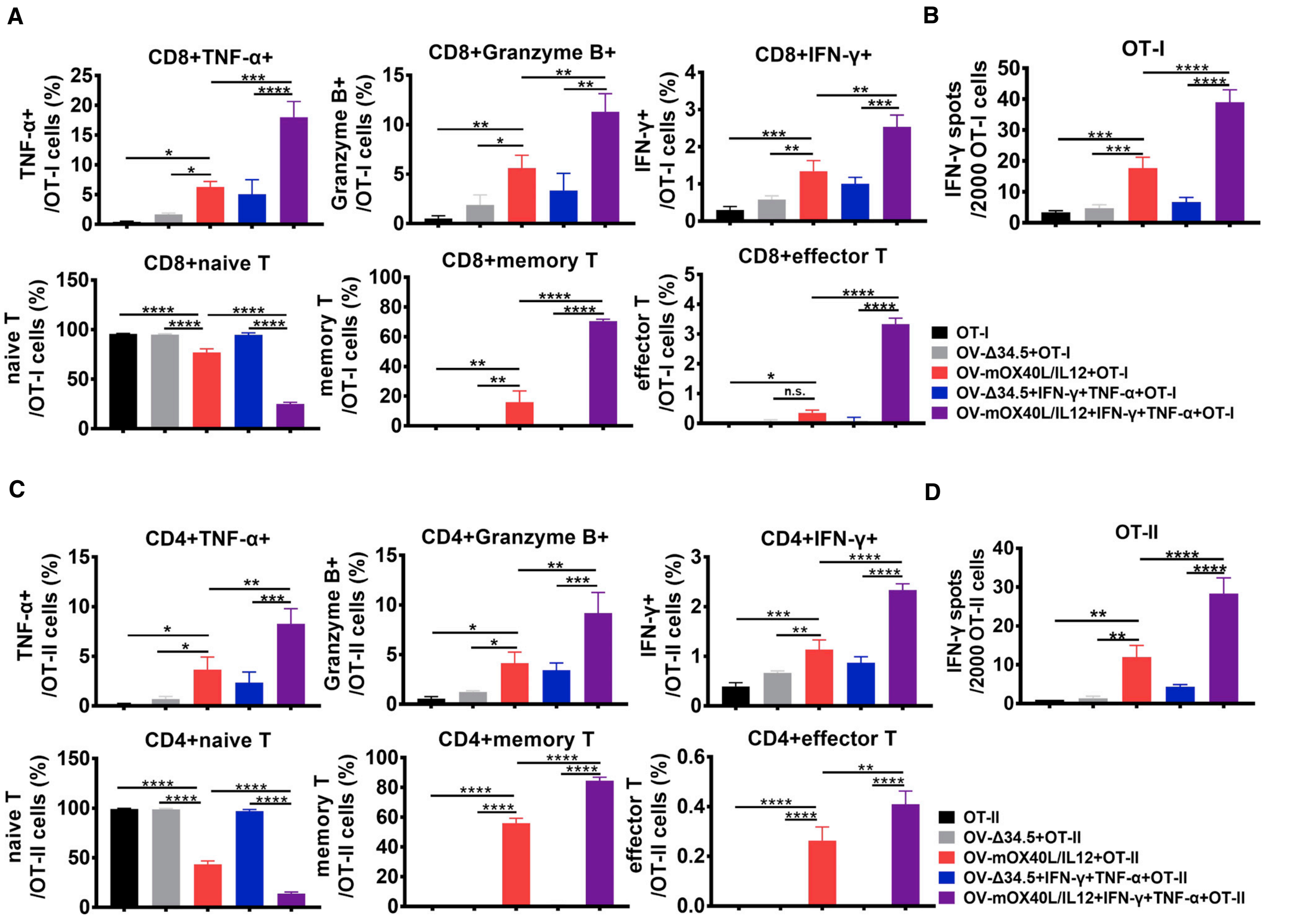

Figure 7. Effect of OV-mOX40L/IL12-infected tumor cells on activating naive T cells

OT-I naive T cells (A, B) or OT-II naive T cells (C, D) were cocultured with MC38-OVA cells pretreated with OV-Δ34.5 or OV-mOX40L/IL12. T cells were stained and subjected to a flow cytometry analysis (A, C) and IFN-γ ELISpot assay (B, D) to profile T cell phenotype and activation status (n = 3). Statistical significance was determined by using one-way ANOVA, followed by Tukey's multiple comparison test analysis. All values are presented as the mean ± SEM. **p < 0.01, ***p < 0.001, and ****p < 0.0001.

to improve the clinical effectiveness of ACT with TILs or CAR-T cells in recent years.[44–46] Recently, Santos et al. reported the possibility of using a TNF-α- and IL2-encoding adenovirus (TILT-123) to enhance the tumor reactivity of TILs in the context of the immunosuppressive environment of ovarian cancer.[47] Notably, Feist et al. reported that an IL2-armed OV promoted the generation and infiltration of tumor-reactive TILs in poorly immunogenic tumors as a pre-TIL approach, and tumor-reactive TILs were harvested and expanded for ACT.[48] In addition to shifting the tumor microenvironment similarly to these reports, our study also focused on converting tumor cells to aAPCs to promote local tumor-specific TILs. However, we envision that OV-OX40L/IL12 integrated into TIL therapy as a pre-TIL procedure before harvesting TILs for ACT may be also beneficial.

Notably, mRNA-based cancer immunotherapy is an emerging modality for the transient and local translation of cytokines.[49] Intratumor delivery of an mRNA-encoding cytokine mixture composed of IL12, IFN-α, GM-CSF, IL15, IL12, IL23, IL36, and OX40L promoted antitumor immunity and tumor regression in preclinical tumor models and clinical trials.[50–54] OV-based and mRNA-based gene therapies represent two classes of cancer gene therapies. OV treatment is different from mRNA-based therapy because OVs replicate inside tumor cells. However, antiviral immunity may limit the oncolytic activity of OVs. We found that the virus titer was below the detection level 3 days after the administration of the virus,[55] suggesting that the long-lasting anticancer effect of OVs is attributed to their elicitation of a robust antitumor immune response rather than the direct oncolytic activity at the dose tested.

In summary, an OV encoding OX40L and IL12 is a potent tumor-specific oncolytic immunotherapy that transforms tumor cells into aAPCs *in situ* and sensitizes solid tumors to TIL therapy. The

results have inspired further investigation of OV-OX40L/IL12 in combination with ACT-TIL and other T cell-centered therapies for the treatment of solid tumors.

MATERIALS AND METHODS

Animal and tumor cell lines

All animal experiments were performed in accordance with and approved by the Institutional Animal Care And Use Committee (IACUC) of Nankai University. Five-week-old male C57BL/6J mice were obtained from Vital River Laboratories. NOD/SCID/IL2R−/− (NSG) mice were obtained from the Shanghai Model Organisms Center.

The human glioma cell line (SHG-44), human tongue squamous cell carcinoma cell line (SCC-15), human breast cancer cell line (MCF-7), human fibrosarcoma cell line (HT-1080), and human colon cancer cell line (HT-29) were cultured in DMEM supplemented with 10% (v/v) fetal bovine serum (FBS) and 1% penicillin/streptomycin. Pan02-HVEM cells were transduced with a lentivirus encoding human HVEM,[55] and the expression of HVEM was monitored by using a flow cytometry analysis with an anti-HVEM antibody (R&D Systems, MAB356). Pan02-HVEM and MC38 cells were also cultured in DMEM containing 10% FBS and 1% penicillin/streptomycin. All cells were incubated at 37°C with 5% CO_2.

OV modification

The donor plasmid containing the homology arms of ICP34.5 flanking a GFP-expressing cassette was transfected into 293 cells, followed by infection with wild-type HSV-1. The produced viruses were harvested, and diluted viruses were then used to infect Vero cells. GFP-positive plaques were selected under a fluorescence microscope. The resulting ICP34.5-negative virus OV-Δ34.5 and ICP34.5-negative and GFP-positive virus OV-GFP were confirmed by DNA sequencing. The ICP47 gene in the ICP34.5-negative virus was replaced with a red fluorescent protein (RFP)-expressing cassette using an approach similar to that described above. Finally, the RFP gene was deleted, and Us11 became an early expression gene. The modified virus was named OV-GFP and used as a template for producing OV-OX40L, OV-IL12, or OV-OX40L/IL12.

The donor plasmid containing homology arms of ICP34.5 flanking OX40L or the IL12-expressing cassette was transfected into 293FT cells followed by infection with the OV-GFP described above. The produced viruses were harvested and used to infect Vero cells. GFP-negative plaques were picked under a fluorescence microscope, and PCR was performed to identify the insertion of OX40L and IL12 into the viral genome.

Similarly, the GFP expression cassette was inserted into the UL26-UL27 intergenic region of OV-OX40L to generate OV-OX40L/GFP. The donor plasmid containing the homology arms of UL26 and UL27 flanking the IL12 expression cassette was transfected into 293FT cells, followed by infection with OV-OX40L/GFP. As a method to increase the efficiency of identification of the correct recombinant sequences, flow cytometry was used to isolate cells that were successfully modified.

Virus propagation and preparation

The viruses were further purified for *in vivo* injection. Vero cells were infected with the indicated viruses at a multiplicity of infection (MOI) = 0.01. The culture medium was collected at 72 h post infection (hpi), and the supernatant was passed through a HiScreen Capto Core 700 (GE Healthcare, 17-5481-15) to obtain purified viruses. The purified viruses were concentrated in an ultrafiltration tube (Millipore, UFC910096) and resuspended in HSV-1 protection solution (PBS containing 6% sorbitol and 12% inositol). The viral titer was determined by counting the number of oncolytic plaques on a monolayer of Vero cells infected with serial dilutions of concentrated virus. A Vero cell HCP ELISA Kit (Cygnus, F500) was used to detect the residual host protein in the purified viruses according to the manufacturer's protocol.

TIL production

TILs were produced as described previously.[56–58] Tumor tissue was minced into small pieces and digested in cell digestion solution (collagenase, 1.25 mg/mL, Sigma, C5138; hyaluronidase, 0.375 mg/mL, Sigma, H2126; and deoxyribonuclease I, 0.0375 mg/mL, Sigma, D5025). The digested cells were passed through a 70-μm cell strainer (Falcon, 352350), washed with PBS, and resuspended in red blood cell (RBC) lysis buffer (Solarbio, R1010). After one wash with PBS, the cell pellet was resuspended in cell culture medium (CCM) (RPMI 1640 medium supplemented with 10% FBS, penicillin/streptomycin [100 U/mL], sodium pyruvate [1 mM], minimum essential medium with nonessential amino acids [MEM-NEAA, 100 μM], β-mercaptoethanol [14.3 μM], L-glutamine [2 mM], gentamicin [10 μg/mL], and amphotericin [2.5 μg/mL]), seeded in a 24-well plate, and cultured overnight.[58] The suspended cells were collected, and TILs were enriched by Percoll gradient density centrifugation. The TILs were resuspended in 50 mL of REP Media I medium (CCM supplemented with IL2 [5 ng/mL, PeproTech, AF-200-02-10], IL7 [10 ng/mL, PeproTech, AF-200-07-10], IL15 [10 ng/mL, PeproTech, AF-200-07-10], and OKT-3 [50 ng/mL, Acro, CDE-M120a]).[57] After the initial outgrowth of T cells (approximately 2 weeks), TILs were rapidly expanded in REP Media II medium. REP Media II medium consisted of a 1 to 1 mixture of REP Media I medium to AIM V medium (Invitrogen, 12055083). TILs were expanded for approximately 3 weeks.

Tumor cells were treated with decitabine (DEC) medium containing decitabine (10 μM, Solarbio, D9010), IFN-γ (100 U/mL, ACRO, IFG-H4211), and TNF-α (10 ng/mL, ACRO, TNA-H4211) for 48 h.[57] TILs were cocultured with pretreated tumor cells for 96 h and then cultured for 12 h in cytokine-free medium followed by stimulation with autologous tumor cells for 6 h. After coculture for 6 h, TILs were isolated using CD45 magnetic beads (MACS, 130-118-780) and intratumorally injected into mice.

Monitoring the effects of OVs on tumor cells with an MTT assay

Cells were mock infected or infected with the indicated viruses at an MOI = 1. At the indicated time points, the cells were incubated with MTT solution (5 mg/mL, Beyotime, ST316) for 4 h at 37°C. The supernatant was aspirated, and 150 μL of dimethyl sulfoxide (DMSO; Solarbio, D8371) were added to each well and incubated in an incubator for 30 min. The absorbance of each well was determined at 490 nm.

Monitoring the effects of OVs on primary tumor tissue with alamarBlue reagent

Primary tumor tissues were cut into 10 mm^3 tissue blocks and seeded in wells of a microplate. alamarBlue cell viability detection reagent (Solarbio, A7631) was added to the tumor tissues and incubated for 1 h at 37°C. After the incubation, the fluorescence was detected with a plate reader (MD, SpectraMax i3x) (excitation light of 530 nm; emission light of 590 nm).

Tumor cell and TIL coculture assay

Tumor samples were digested into a single-cell suspension, washed, and resuspended in DMEM containing 20% FBS and 2 mM L-glutamine (Solarbio, G0200). Tumor cells were treated with DEC medium containing decitabine (10 μM), IFN-γ (100 U/mL), and TNF-α (10 ng/mL) for 48 h and TILs were added at different TIL to tumor cell ratios. After an incubation at 37°C with 5% CO_2 for 24 h, 20 μL of supernatant were collected for the analysis of IFN-γ levels using an ELISA kit (R&D Systems, VAL104).

ELISpot assay

ELISpot assays were performed using an IFN-γ precoated ELISpot kit according to the manufacturer's instructions (Dakewe Biotech). Tumor cells were infected with different OVs (MOI = 0.01) followed by the addition of tumor-primed TILs. After 24 h of coculture, TILs were added to precoated ELISpot wells and incubated for 24 h. After the incubation, captured IFN-γ was detected by using biotinylated IFN-γ antibody followed by the addition of streptavidin-horseradish peroxidase (HRP) and an aminoethyl carbazole (AEC) solution. Spots were analyzed and counted with an automated plate reader (Dakewe Biotech).

Real-time RT-PCR analysis of gene expression in infected tumor cells

Infected tumor cells were lysed using Cell Lysis Buffer (TRIzol, Thermo Fisher, 15596018), and cDNAs were synthesized from cell lysates using a HiScript 1st Strand cDNA Synthesis Kit (Vazyme Biotech, R111-01). Real-time PCR analysis was performed on the resulting cDNAs using FS Universal SYBR Green Master Rox (Roche, 4913850001) and the ABI 7700 Sequence Detection System.

scTCR-seq and scRNA-seq analyses of TILs cocultured with OV-infected tumor cells

TILs were cocultured with OV-infected or mock-infected primary tumor cells. TILs from both groups were isolated using CD45 magnetic beads (MACS, 130-118-780) and subjected to scTCR-seq and scRNA-seq analyses using the 10x Genomics Chromium platform. Gene expression sequencing reads from scRNA-seq and the TCR variable-diversity-joining (VDJ) sequencing reads were mapped to the GRCh38 genome and the reference dataset vdj_GRCh38_alts_ensembl-5.0.0 using the CellRanger software package (version 6.0.2). Single-cell transcriptomes were analyzed using Seurat (version 3.2.3).[59] Cells with fewer than 200 or more than 5,000 detected genes, cells with more than 20,000 unique molecular identifiers (UMIs), and cells in which mitochondrial protein-coding genes represented more than 10% of the UMI content were excluded. Paired TCRα and TCRβ chains in single cells were identified by their shared barcodes. Cells containing only one productive TCRα chain and one productive TCRβ chain were retained in scRepertoire (v1.3.3).[60] The number of germline TCR genes was calculated using scRepertoire. Only the cells with both TCR and scRNA-seq data were subjected to a downstream analysis. Cells with the same CDR3 peptide sequence in both TCRα and TCRβ chains were considered to belong to the same TCR clonotype. The frequency of each clonotype in each sample was then calculated as the clone count. The clone size proportion was calculated as the clone count divided by the number of cells per sample. scRepertoire added the TCR clonotype information to the Seurat object as metadata. For every clonotype in each sample, we calculated the percentage of cells that expressed IFN-γ.

Establishment of the PDX model

All patient-related studies were performed in accordance with the Declaration of Helsinki. Primary tumor samples were obtained from patients undergoing surgery for head and neck squamous cell carcinoma at Tianjin Stomatological Hospital who signed informed consent forms, and the procedure was approved by the Institutional Ethics Committee of Tianjin Stomatological Hospital. Immunocompromised NSG mice were used for implantation of patient tumors. The tumor tissues were preserved in DMEM supplemented with 10% FBS, 1% penicillin-streptomycin solution, 2.5 μg/mL amphotericin, and 50 μg/mL gentamicin. Tumor samples were cut into 2-mm pieces, and three pieces of tumor tissue were mixed with Matrigel matrix (Biocoat, 354234) and subcutaneously implanted into the left flank of each NSG mouse. After implantation, PDX growth was monitored three times per week by using Vernier calipers. Passage 1 (P1) PDX tumors were extracted, minced into approximately 10-mm^3 pieces, and implanted into the left flanks of NSG mice to generate passage 2 (P2) PDX tumor models.

When the PDX tumor reached 200–300 mm^3, mice were intratumorally administered a single dose of 2×10^5 plaque-forming units (PFU) of OVs per mouse. For the combination therapy experiments, mice were intratumorally injected with 2×10^6 TILs per mouse 2 days after the OV injection. The mice were intraperitoneally (i.p.) injected with 10 μg of IL2 every other day from days 2 to 18 to support TIL persistence *in vivo*.

Isolation of OT-I/II-naive T cells

CD8/CD4-naive T cells were isolated from the spleens of OT-I/II TCR transgenic mice by using a Naive CD8a$^+$ T Cell Isolation Kit

(Miltenyi) and a Naive CD4$^+$ T Cell Isolation Kit (Miltenyi) according to the manufacturer's instructions.

Syngeneic tumor mouse models

Approximately 10^5 viable MC38 or Pan02-HVEM cells were subcutaneously implanted into the right flank of 6-week-old mice. Tumor growth was measured two to three times per week with calipers. When tumor volumes reached approximately 50 mm^3, mice were intratumorally injected with two doses of 2×10^6 PFU of OVs per mouse. For combination therapy experiments, mice were intratumorally injected with 10^6 TILs per mouse 2 days after the second OV treatment. Tumor growth was measured two to three times per week by using calipers. When tumor volumes reached 1,500 mm^3, mice were euthanized according to the protocol approved by IACUC, and the Kaplan-Meier survival analysis was performed using GraphPad Prism software.

For the rechallenge experiments, mice with complete responses to the combination therapy were injected with the same type of tumor cells (5×10^5 cells per mouse) in the flank opposite the initial injection site. The same number of tumor cells was implanted into treatment-naive mice to confirm tumor growth.

Antibody depletion was performed using blocking antibodies for *in vivo* depletion of CD8$^+$, CD4$^+$, and NK cells using a protocol similar to that described previously.[61] C57BL/6J mice (n = 4 mice/group) were depleted of CD8$^+$, CD4$^+$, and NK1.1$^+$ cells using 250 μg of relevant anti-NK1.1, anti-CD4, or anti-CD8 antibodies in 200 μL. Mice were injected with InVivoMab anti-mouse CD4 (anti-CD4), InVivoMab anti-mouse CD8α (anti-CD8), or InVivoMab anti-mouse NK1.1 (anti-NK1.1) i.p. once a day before each treatment and once a week after the treatment until the mice died. The tumor volume was measured every 2 days.

For bilateral inoculation, MC38 tumor cells (1×10^6 cells per injection site) were subcutaneously inoculated into the right and left flanks of mice at the same time. When tumors reached approximately 100 mm^3, 2×10^6 PFU of OV-mOX40L/IL12 were injected into the tumors on days 7 and 9. On day 11, mice received an intratumor injection of TILs (1×10^6 cells).

Splenocytes from OT-I TCR transgenic mice (Shanghai Model Organisms) were cultured in the presence of 200 ng/mL mIL2 (Sino Biological), 100 ng/mL Ultra-LEAF purified anti-mouse CD3ε antibody (clone 145-2C11, BioLegend), and 100 ng/mL Ultra-LEAF purified anti-mouse CD28 antibody (clone 37.51, BioLegend) for 7 days before the enrichment of CD8$^+$ T cells was performed with a CD8$^+$ T Cell Isolation Kit II (Miltenyi) according to the manufacturer's instructions. Enriched T cells were cultured further in the presence of mIL2, anti-CD3ε, and anti-CD28 antibodies for 7 days before adoptive transfer into MC38-OVA tumor-bearing mice. When tumors reached approximately 100 mm^3, 2×10^6 PFU of OV-mOX40L/IL12 were injected into the tumors on days 7 and 9. On days 11, 13, and 15, the mice were injected with 1×10^7 CD8$^+$-enriched OT-I T cells in 500 μL of PBS via the tail vein.

IHC analysis

Tumor tissues were harvested and fixed for 72 h with 4% paraformaldehyde (Solarbio) before storage in 70% ethanol until further processing. Paraffin-embedded tumor sections (10 μm) were deparaffinized and subjected to heat-mediated antigen retrieval for 30 min in sodium citrate antigen retrieval solution (Beyotime). After antigen retrieval, tumor sections were permeabilized with 100% methanol. Sections were blocked for 30 min with goat serum (Solarbio) and then incubated with a rabbit anti-IFN-γ polyclonal antibody (Bioss), rabbit anti-HLA-A/B/C polyclonal antibody (Bioss), rabbit anti-HLA-DR monoclonal antibody (Bioss), rabbit anti-CD134 polyclonal antibody (Bioss), rabbit anti-CD137 polyclonal antibody (Bioss), or rabbit anti-CD86 polyclonal antibody (Bioss) diluted 1:100 in goat serum overnight in a humidified chamber at 4°C (Table S1). After the incubation, tumor sections were washed and incubated with a goat anti-rabbit secondary antibody (ZSGB-Bio) for 1 h at room temperature. The sections were stained with diaminobenzidine (DAB, ZSGB-Bio). Images were obtained using a Nanozoomer 2.0HT digital slide scanner and the associated NDP.view2 software (Hamamatsu).

Flow cytometry analysis

Resected tumor samples and tumor-draining lymph nodes (tdLNs) were cut into small pieces and incubated with digestion solution (Hank's balanced salt solution, Thermo Fisher) containing collagenase type IV (0.4 mg/mL) and deoxyribonuclease I (0.2 mg/mL) for 30 min. Tumor and tumor-infiltrating mononuclear cells were separated by Percoll gradient centrifugation (40%/70%). Splenocytes were isolated from excised spleens by filtering them through a 70-μm strainer. Erythrocytes were lysed using RBC lysis buffer (Solarbio, R1010). The tumor cells were stained with panels of antibodies to characterize the expression of APC-related proteins. Immune cells from tumors/tdLNs and splenocytes were blocked using TruStain FcX (anti-mouse CD16/32) (BioLegend, 101320) to avoid nonspecific binding and stained using four panels of antibodies (Table S2) to profile the frequency and activation status of lymphocytes, myeloid cells, and DCs. Samples were then subjected to a flow cytometry analysis (LSR Fortessa), and the data were analyzed using FlowJo software.

T cells cocultured with tumor cells were collected and blocked using TruStain FcX (anti-mouse CD16/32), and stained using panels of antibodies (Table S2) to characterize the frequency and activation status of the lymphocytes.

Serum cytokine profile

No less than 150 μL of serum samples were packed in dry ice and sent to the LabEx laboratory of Shanghai Universal Biotech Company. Cytokine profiles were assessed using the Bio-Plex Pro Mouse Cytokine 23-plex panel (Bio-Rad Laboratories). The cytokines analyzed included IL1α, IL1β, IL2, IL3, IL4, IL5, IL6, IL9, IL10, IL12 p40, IL12 p70, IL13, IL17, eotaxin (CCL11), CXCL-1, G-CSF, GM-CSF,

IFN-γ, MCP-1, MIP-1α, MIP-1β, RANTES, and TNF-α. Experiments were conducted by LabEx.

Statistical analysis

Statistical analyses were determined by one-way ANOVA, followed by Tukey's multiple comparison test analysis. Percentage survival was estimated using the Kaplan-Meier method, and statistical significance was calculated by the log rank test. All statistical analyses were conducted using GraphPad Prism 7 (GraphPad). Significance of differences is reported as *p < 0.05, **p < 0.01, ***p < 0.001, and ****p < 0.0001. NS, not significant.

SUPPLEMENTAL INFORMATION

Supplemental information can be found online at https://doi.org/10.1016/j.ymthe.2022.06.010.

ACKNOWLEDGMENTS

We thank Dr. He Zhou (Migrasome Therapeutics), Dr. Yuanqiao He (Nanchang Royo Biotech Co,. Ltd), Dr. Long Li, Dr. Jie Yin (Tianjin Medical University), Dr. Jun Chen (Sun Yat-Sen University), and Dr. Lihua Yin (Lanzhou University) for participating in scientific discussions. The construction of the second PDX model was supported by Nanchang Royo Biotech Co,. Ltd. This work was supported by the Fundamental Research Funds for the Central Universities, Nankai University (grant number 63223035), the Key Research Fund of Tianjin Project & Team (grant number XB202010), the Key Research and Development Program of Tianjin (grant number 20YFZCSY00450), Tianjin Key Medical Discipline (Specialty) Construction Project, the Natural Science Foundation of Tianjin (grant number 19JCZDJC32900), and the grant 17-74-30019. The research was also funded by the State Key Laboratory of Medicinal Chemical Biology open funding (grant numbers 2019022 and 2010022) and CNBG-Nankai University Joint Research and Development Center.

AUTHOR CONTRIBUTIONS

H.Z. and Y.Y. conceived the study. H.Z. and Y.Y. supervised the project. H.Z., Y.Y., and K.Y. designed all experiments. K.Y., F.L., R.W., T.C., Z.Z., R.L., L.X., G.Z., Z.X., W.L., S.L., and Y.W. performed the experiments. K.Y., F.L., T.C., and L.X. generated the recombinant viruses. K.Y., R.W., T.C., Z.Z., and L.X. conducted the *in vivo* experiments. L.D. and L.L. assisted with the viral construction, production, and characterization. L.K.Y., R.W., and Y.W. analyzed the fluorescence-activated cell sorting (FACS) data. G.Z. and Z.X. collected the patient samples and performed the IHC experiments. W.W. and H.Z. interpreted the scRNA-seq data. H.Z., Y.Y., K.Y., Y.G., M.Z., A.S., and A.G. discussed the results and wrote the manuscript.

DECLARATION OF INTEREST

L.D. and L.L. are employees of Beijing Institute of Biological Products. The remaining authors declare no competing interests.

REFERENCES

1. Sarnaik, A.A., Hamid, O., Khushalani, N.I., Lewis, K.D., Medina, T., Kluger, H.M., Thomas, S.S., Domingo-Musibay, E., Pavlick, A.C., Whitman, E.D., et al. (2021). Lifileucel, a tumor-infiltrating lymphocyte therapy, in metastatic melanoma. J. Clin. Oncol. *39*, 2656–2666. https://doi.org/10.1200/jco.21.00612.
2. Dudley, M.E., Wunderlich, J.R., Robbins, P.F., Yang, J.C., Hwu, P., Schwartzentruber, D.J., Topalian, S.L., Sherry, R., Restifo, N.P., Hubicki, A.M., et al. (2002). Cancer regression and autoimmunity in patients after clonal repopulation with antitumor lymphocytes. Science *298*, 850–854. https://doi.org/10.1126/science.1076514.
3. Rosenberg, S.A., Yannelli, J.R., Yang, J.C., Topalian, S.L., Schwartzentruber, D.J., Weber, J.S., Parkinson, D.R., Seipp, C.A., Einhorn, J.H., and White, D.E. (1994). Treatment of patients with metastatic melanoma with autologous tumor-infiltrating lymphocytes and interleukin 2. J. Natl. Cancer Inst. *86*, 1159–1166. https://doi.org/10.1093/jnci/86.15.1159.
4. Creelan, B.C., Wang, C., Teer, J.K., Toloza, E.M., Yao, J., Kim, S., Landin, A.M., Mullinax, J.E., Saller, J.J., Saltos, A.N., et al. (2021). Tumor-infiltrating lymphocyte treatment for anti-PD-1-resistant metastatic lung cancer: a phase 1 trial. Nat. Med. *27*, 1410–1418. https://doi.org/10.1038/s41591-021-01462-y.
5. Sabado, R.L., Balan, S., and Bhardwaj, N. (2017). Dendritic cell-based immunotherapy. Cell Res. *27*, 74–95. https://doi.org/10.1038/cr.2016.157.
6. Harari, A., Graciotti, M., Bassani-Sternberg, M., and Kandalaft, L.E. (2020). Antitumour dendritic cell vaccination in a priming and boosting approach. Nat. Rev. Drug Discov. *19*, 635–652. https://doi.org/10.1038/s41573-020-0074-8.
7. de Charette, M., Marabelle, A., and Houot, R. (2016). Turning tumour cells into antigen presenting cells: the next step to improve cancer immunotherapy? Eur. J. Cancer *68*, 134–147. https://doi.org/10.1016/j.ejca.2016.09.010.
8. Etxeberria, I., Olivera, I., Bolaños, E., Cirella, A., Teijeira, Á., Berraondo, P., and Melero, I. (2020). Engineering bionic T cells: signal 1, signal 2, signal 3, reprogramming and the removal of inhibitory mechanisms. Cell. Mol. Immunol. *17*, 576–586. https://doi.org/10.1038/s41423-020-0464-1.
9. Suhoski, M.M., Golovina, T.N., Aqui, N.A., Tai, V.C., Varela-Rohena, A., Milone, M.C., Carroll, R.G., Riley, J.L., and June, C.H. (2007). Engineering artificial antigen-presenting cells to express a diverse array of co-stimulatory molecules. Mol. Ther. *15*, 981–988. https://doi.org/10.1038/mt.sj.6300134.
10. Forget, M.A., Malu, S., Liu, H., Toth, C., Maiti, S., Kale, C., Haymaker, C., Bernatchez, C., Huls, H., Wang, E., et al. (2014). Activation and propagation of tumor-infiltrating lymphocytes on clinical-grade designer artificial antigen-presenting cells for adoptive immunotherapy of melanoma. J. Immunother. *37*, 448–460. https://doi.org/10.1097/cji.0000000000000056.
11. Latouche, J.B., and Sadelain, M. (2000). Induction of human cytotoxic T lymphocytes by artificial antigen-presenting cells. Nat. Biotechnol. *18*, 405–409. https://doi.org/10.1038/74455.
12. Thomas, A.K., Maus, M.V., Shalaby, W.S., June, C.H., and Riley, J.L. (2002). A cell-based artificial antigen-presenting cell coated with anti-CD3 and CD28 antibodies enables rapid expansion and long-term growth of CD4 T lymphocytes. Clin. Immunol. *105*, 259–272. https://doi.org/10.1006/clim.2002.5277.
13. Yee, C., Thompson, J.A., Byrd, D., Riddell, S.R., Roche, P., Celis, E., and Greenberg, P.D. (2002). Adoptive T cell therapy using antigen-specific CD8+ T cell clones for the treatment of patients with metastatic melanoma: in vivo persistence, migration, and antitumor effect of transferred T cells. Proc. Natl. Acad. Sci. USA *99*, 16168–16173. https://doi.org/10.1073/pnas.242600099.
14. Butler, M.O., and Hirano, N. (2014). Human cell-based artificial antigen-presenting cells for cancer immunotherapy. Immunol. Rev. *257*, 191–209. https://doi.org/10.1111/imr.12129.
15. Ostrand-Rosenberg, S. (1994). Tumor immunotherapy: the tumor cell as an antigen-presenting cell. Curr. Opin. Immunol. *6*, 722–727. https://doi.org/10.1016/0952-7915(94)90075-2.
16. Wan, P.K.T., Ryan, A.J., and Seymour, L.W. (2021). Beyond cancer cells: targeting the tumor microenvironment with gene therapy and armed oncolytic virus. Mol. Ther. *29*, 1668–1682. https://doi.org/10.1016/j.ymthe.2021.04.015.
17. Twumasi-Boateng, K., Pettigrew, J.L., Kwok, Y.Y.E., Bell, J.C., and Nelson, B.H. (2018). Oncolytic viruses as engineering platforms for combination immunotherapy. Nat. Rev. Cancer *18*, 419–432. https://doi.org/10.1038/s41568-018-0009-4.
18. Harrington, K., Freeman, D.J., Kelly, B., Harper, J., and Soria, J.C. (2019). Optimizing oncolytic virotherapy in cancer treatment. Nat. Rev. Drug Discov. *18*, 689–706. https://doi.org/10.1038/s41573-019-0029-0.

19. Rehman, H., Silk, A.W., Kane, M.P., and Kaufman, H.L. (2016). Into the clinic: Talimogene laherparepvec (T-VEC), a first-in-class intratumoral oncolytic viral therapy. J. Immunother. Cancer *4*, 53. https://doi.org/10.1186/s40425-016-0158-5.

20. Nakao, S., Arai, Y., Tasaki, M., Yamashita, M., Murakami, R., Kawase, T., Amino, N., Nakatake, M., Kurosaki, H., Mori, M., et al. (2020). Intratumoral expression of IL-7 and IL-12 using an oncolytic virus increases systemic sensitivity to immune checkpoint blockade. Sci. Transl. Med. *12*, eaax7992. https://doi.org/10.1126/scitranslmed.aax7992.

21. Malmström, P.U., Loskog, A.S., Lindqvist, C.A., Mangsbo, S.M., Fransson, M., Wanders, A., Gårdmark, T., and Tötterman, T.H. (2010). AdCD40L immunogene therapy for bladder carcinoma–the first phase I/IIa trial. Clin. Cancer Res. *16*, 3279–3287. https://doi.org/10.1158/1078-0432.ccr-10-0385.

22. Zafar, S., Sorsa, S., Siurala, M., Hemminki, O., Havunen, R., Cervera-Carrascon, V., Santos, J.M., Wang, H., Lieber, A., De Gruijl, T., et al. (2018). CD40L coding oncolytic adenovirus allows long-term survival of humanized mice receiving dendritic cell therapy. Oncoimmunology *7*, e1490856. https://doi.org/10.1080/2162402x.2018.1490856.

23. Wang, R., Chen, J., Wang, W., Zhao, Z., Wang, H., Liu, S., Li, F., Wan, Y., Yin, J., Wang, R., et al. (2022). CD40L-armed oncolytic herpes simplex virus suppresses pancreatic ductal adenocarcinoma by facilitating the tumor microenvironment favorable to cytotoxic T cell response in the syngeneic mouse model. J. Immunother. Cancer *10*, e003809. https://doi.org/10.1136/jitc-2021-003809.

24. Gu, Y.Z., Zhao, X., and Song, X.R. (2020). Ex vivo pulsed dendritic cell vaccination against cancer. Acta Pharmacol. Sin. *41*, 959–969. https://doi.org/10.1038/s41401-020-0415-5.

25. Fu, C., Zhou, L., Mi, Q.S., and Jiang, A. (2020). DC-based vaccines for cancer immunotherapy. Vaccines (Basel) *8*, 706. https://doi.org/10.3390/vaccines8040706.

26. Turtle, C.J., and Riddell, S.R. (2010). Artificial antigen-presenting cells for use in adoptive immunotherapy. Cancer J. *16*, 374–381. https://doi.org/10.1097/ppo.0b013e3181eb33a6.

27. Jiang, Y., Krishnan, N., Zhou, J., Chekuri, S., Wei, X., Kroll, A.V., Yu, C.L., Duan, Y., Gao, W., Fang, R.H., et al. (2020). Engineered cell-membrane-coated nanoparticles directly present tumor antigens to promote anticancer immunity. Adv. Mater. *32*, e2001808. https://doi.org/10.1002/adma.202001808.

28. Liu, C., Liu, X., Xiang, X., Pang, X., Chen, S., Zhang, Y., Ren, E., Zhang, L., Liu, X., Lv, P., et al. (2022). A nanovaccine for antigen self-presentation and immunosuppression reversal as a personalized cancer immunotherapy strategy. Nat. Nanotechnol. *17*, 531–540. https://doi.org/10.1038/s41565-022-01098-0.

29. Rosa, F.F., Pires, C.F., Kurochkin, I., Ferreira, A.G., Gomes, A.M., Palma, L.G., Shaiv, K., Solanas, L., Azenha, C., Papatsenko, D., et al. (2018). Direct reprogramming of fibroblasts into antigen-presenting dendritic cells. Sci. Immunol. *3*, eaau4292. https://doi.org/10.1126/sciimmunol.aau4292.

30. Jiang, H., Rivera-Molina, Y., Gomez-Manzano, C., Clise-Dwyer, K., Bover, L., Vence, L.M., Yuan, Y., Lang, F.F., Toniatti, C., Hossain, M.B., et al. (2017). Oncolytic adenovirus and tumor-targeting immune modulatory therapy improve autologous cancer vaccination. Cancer Res. *77*, 3894–3907. https://doi.org/10.1158/0008-5472.can-17-0468.

31. Alspach, E., Lussier, D.M., Miceli, A.P., Kizhvatov, I., DuPage, M., Luoma, A.M., Meng, W., Lichti, C.F., Esaulova, E., Vomund, A.N., et al. (2019). MHC-II neoantigens shape tumour immunity and response to immunotherapy. Nature *574*, 696–701. https://doi.org/10.1038/s41586-019-1671-8.

32. Boagni, D.A., Ravirala, D., and Zhang, S.X. (2021). Current strategies in engaging oncolytic viruses with antitumor immunity. Mol. Ther. Oncolytics *22*, 98–113. https://doi.org/10.1016/j.omto.2021.05.002.

33. Hansen, T.H., and Bouvier, M. (2009). MHC class I antigen presentation: learning from viral evasion strategies. Nat. Rev. Immunol. *9*, 503–513. https://doi.org/10.1038/nri2575.

34. Steimle, V., Siegrist, C.A., Mottet, A., Lisowska-Grospierre, B., and Mach, B. (1994). Regulation of MHC class II expression by interferon-gamma mediated by the transactivator gene CIITA. Science *265*, 106–109. https://doi.org/10.1126/science.8016643.

35. Bartlett, D.L., Liu, Z., Sathaiah, M., Ravindranathan, R., Guo, Z., He, Y., and Guo, Z.S. (2013). Oncolytic viruses as therapeutic cancer vaccines. Mol. Cancer *12*, 103. https://doi.org/10.1186/1476-4598-12-103.

36. Ye, Z., Xu, S., Moyana, T., Yang, J., and Xiang, J. (2008). Defect of CD8+ memory T cells developed in absence of IL-12 priming for secondary expansion. Cell. Mol. Immunol. *5*, 147–152. https://doi.org/10.1038/cmi.2008.18.

37. Le Bon, A., Etchart, N., Rossmann, C., Ashton, M., Hou, S., Gewert, D., Borrow, P., and Tough, D.F. (2003). Cross-priming of CD8+ T cells stimulated by virus-induced type I interferon. Nat. Immunol. *4*, 1009–1015. https://doi.org/10.1038/ni978.

38. Curtsinger, J.M., and Mescher, M.F. (2010). Inflammatory cytokines as a third signal for T cell activation. Curr. Opin. Immunol. *22*, 333–340. https://doi.org/10.1016/j.coi.2010.02.013.

39. Xiao, Z., Casey, K.A., Jameson, S.C., Curtsinger, J.M., and Mescher, M.F. (2009). Programming for CD8 T cell memory development requires IL-12 or type I IFN. J. Immunol. *182*, 2786–2794. https://doi.org/10.4049/jimmunol.0803484.

40. Kieper, W.C., Prlic, M., Schmidt, C.S., Mescher, M.F., and Jameson, S.C. (2001). Il-12 enhances CD8 T cell homeostatic expansion. J. Immunol. *166*, 5515–5521. https://doi.org/10.4049/jimmunol.166.9.5515.

41. Nguyen, H.M., Guz-Montgomery, K., and Saha, D. (2020). Oncolytic virus encoding a master pro-inflammatory cytokine interleukin 12 in cancer immunotherapy. Cells *9*, 400. https://doi.org/10.3390/cells9020400.

42. Omar, N.B., Bentley, R.T., Crossman, D.K., Foote, J.B., Koehler, J.W., Markert, J.M., Platt, S.R., Rissi, D.R., Shores, A., Sorjonen, D., et al. (2021). Safety and interim survival data after intracranial administration of M032, a genetically engineered oncolytic HSV-1 expressing IL-12, in pet dogs with sporadic gliomas. Neurosurg. Focus *50*, E5. https://doi.org/10.3171/2020.11.focus20844.

43. Vannini, A., Leoni, V., and Campadelli-Fiume, G. (2021). Targeted delivery of IL-12 adjuvants immunotherapy by oncolytic viruses. Adv. Exp. Med. Biol. *1290*, 67–80. https://doi.org/10.1007/978-3-030-55617-4_4.

44. Ajina, A., and Maher, J. (2017). Prospects for combined use of oncolytic viruses and CAR T-cells. J. Immunother. Cancer *5*, 90. https://doi.org/10.1186/s40425-017-0294-6.

45. Porter, C.E., Rosewell Shaw, A., Jung, Y., Yip, T., Castro, P.D., Sandulache, V.C., Sikora, A., Gottschalk, S., Ittman, M.M., Brenner, M.K., et al. (2020). Oncolytic adenovirus armed with BiTE, cytokine, and checkpoint inhibitor enables CAR T cells to control the growth of heterogeneous tumors. Mol. Ther. *28*, 1251–1262. https://doi.org/10.1016/j.ymthe.2020.02.016.

46. Wing, A., Fajardo, C.A., Posey, A.D., Jr., Shaw, C., Da, T., Young, R.M., Alemany, R., June, C.H., and Guedan, S. (2018). Improving CART-cell therapy of solid tumors with oncolytic virus-driven production of a bispecific T-cell engager. Cancer Immunol. Res. *6*, 605–616. https://doi.org/10.1158/2326-6066.cir-17-0314.

47. Santos, J.M., Heiniö, C., Cervera-Carrascon, V., Quixabeira, D.C.A., Siurala, M., Havunen, R., Butzow, R., Zafar, S., de Gruijl, T., Lassus, H., et al. (2020). Oncolytic adenovirus shapes the ovarian tumor microenvironment for potent tumor-infiltrating lymphocyte tumor reactivity. J. Immunother. Cancer *8*, e000188. https://doi.org/10.1136/jitc-2019-000188.

48. Feist, M., Zhu, Z., Dai, E., Ma, C., Liu, Z., Giehl, E., Ravindranathan, R., Kowalsky, S.J., Obermajer, N., Kammula, U.S., et al. (2021). Oncolytic virus promotes tumor-reactive infiltrating lymphocytes for adoptive cell therapy. Cancer Gene Ther. *28*, 98–111. https://doi.org/10.1038/s41417-020-0189-4.

49. Beck, J.D., Reidenbach, D., Salomon, N., Sahin, U., Türeci, Ö., Vormehr, M., and Kranz, L.M. (2021). mRNA therapeutics in cancer immunotherapy. Mol. Cancer Ther. *20*, 69. https://doi.org/10.1186/s12943-021-01348-0.

50. Hewitt, S.L., Bai, A., Bailey, D., Ichikawa, K., Zielinski, J., Karp, R., Apte, A., Arnold, K., Zacharek, S.J., Iliou, M.S., et al. (2019). Durable anticancer immunity from intratumoral administration of IL-23, IL-36γ, and OX40L mRNAs. Sci. Transl. Med. *11*, eaat9143. https://doi.org/10.1126/scitranslmed.aat9143.

51. Haabeth, O.A.W., Blake, T.R., McKinlay, C.J., Tveita, A.A., Sallets, A., Waymouth, R.M., Wender, P.A., and Levy, R. (2019). Local delivery of Ox40l, Cd80, and Cd86 mRNA kindles global anticancer immunity. Cancer Res. *79*, 1624–1634. https://doi.org/10.1158/0008-5472.can-18-2867.

52. Hotz, C., Wagenaar, T.R., Gieseke, F., Bangari, D.S., Callahan, M., Cao, H., Diekmann, J., Diken, M., Grunwitz, C., Hebert, A., et al. (2021). Local delivery of mRNA-encoded cytokines promotes antitumor immunity and tumor eradication across multiple preclinical tumor models. Sci. Transl. Med. *13*, eabc7804. https://doi.org/10.1126/scitranslmed.abc7804.

53. Hewitt, S.L., Bailey, D., Zielinski, J., Apte, A., Musenge, F., Karp, R., Burke, S., Garcon, F., Mishra, A., Gurumurthy, S., et al. (2020). Intratumoral IL12 mRNA therapy promotes TH1 transformation of the tumor microenvironment. Clin. Cancer Res. *26*, 6284–6298. https://doi.org/10.1158/1078-0432.ccr-20-0472.

54. Lai, I., Swaminathan, S., Baylot, V., Mosley, A., Dhanasekaran, R., Gabay, M., and Felsher, D.W. (2018). Lipid nanoparticles that deliver IL-12 messenger RNA suppress tumorigenesis in MYC oncogene-driven hepatocellular carcinoma. J. Immunother. Cancer *6*, 125. https://doi.org/10.1186/s40425-018-0431-x.

55. Zhang, L., Wang, W., Wang, R., Zhang, N., Shang, H., Bi, Y., Chen, D., Zhang, C., Li, L., Yin, J., et al. (2021). Reshaping the immune microenvironment by oncolytic herpes simplex virus in murine pancreatic ductal adenocarcinoma. Mol. Ther. *29*, 744–761. https://doi.org/10.1016/j.ymthe.2020.10.027.

56. Tran, E., Turcotte, S., Gros, A., Robbins, P.F., Lu, Y.C., Dudley, M.E., Wunderlich, J.R., Somerville, R.P., Hogan, K., Hinrichs, C.S., et al. (2014). Cancer immunotherapy based on mutation-specific CD4+ T cells in a patient with epithelial cancer. Science *344*, 641–645. https://doi.org/10.1126/science.1251102.

57. Tan, Q., Zhang, C., Yang, W., Liu, Y., Heyilimu, P., Feng, D., Xing, L., Ke, Y., and Lu, Z. (2019). Isolation of T cell receptor specifically reactive with autologous tumour cells from tumour-infiltrating lymphocytes and construction of T cell receptor engineered T cells for esophageal squamous cell carcinoma. J. Immunother. Cancer *7*, 232. https://doi.org/10.1186/s40425-019-0709-7.

58. Kokolus, K.M., Obermajer, N., and Kalinski, P. (2020). Quantitative evaluation of tumor-specific T cells in tumors and lymphoid tissues. Methods Enzymol. *635*, 149–166. https://doi.org/10.1016/bs.mie.2019.05.041.

59. Butler, A., Hoffman, P., Smibert, P., Papalexi, E., and Satija, R. (2018). Integrating single-cell transcriptomic data across different conditions, technologies, and species. Nat. Biotechnol. *36*, 411–420. https://doi.org/10.1038/nbt.4096.

60. Borcherding, N., Bormann, N.L., and Kraus, G. (2020). scRepertoire: an R-based toolkit for single-cell immune receptor analysis. F1000Res. *9*, 47. https://doi.org/10.12688/f1000research.22139.1.

61. Chen, C.H., Wang, T.L., Hung, C.F., Yang, Y., Young, R.A., Pardoll, D.M., and Wu, T.C. (2000). Enhancement of DNA vaccine potency by linkage of antigen gene to an HSP70 gene. Cancer Res. *60*, 1035–1042.

YMTHE, Volume *30*

Supplemental Information

An armed oncolytic virus enhances the efficacy of tumor-infiltrating lymphocyte therapy by converting tumors to artificial antigen-presenting cells *in situ*

Kai Ye, Fan Li, Ruikun Wang, Tianyi Cen, Shiyu Liu, Zhuoqian Zhao, Ruonan Li, Lili Xu, Guanmeng Zhang, Zhaoyuan Xu, Li Deng, Lili Li, Wei Wang, Alexey Stepanov, Yajuan Wan, Yu Guo, Yuanke Li, Yuan Wang, Yujie Tian, Alexander G. Gabibov, Yingbin Yan, and Hongkai Zhang

Supplementary Figure 1

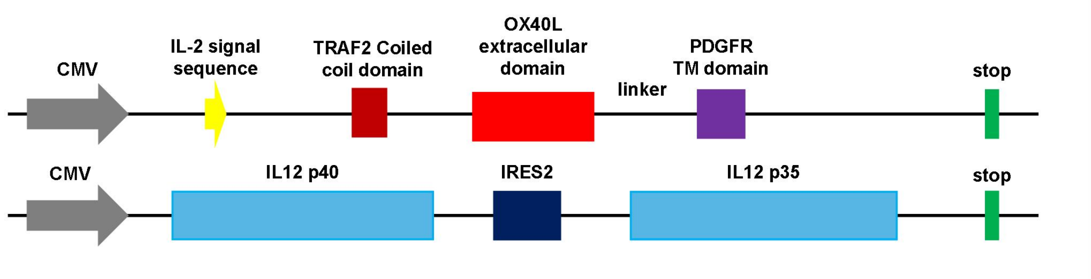

Figure S1. Schematic representation of the trimerized OX40L and IL12 design.

The OX40L extracellular domain is fused to the coiled coil trimerization domain of the TRAF2 protein and the PDGFR transmembrane[1] (TM) domain. The IL12 domain consists of IL12 p40 and IL12 p35, which are linked by internal ribosome entry site 2 (IRES2).

Supplementary Figure 2

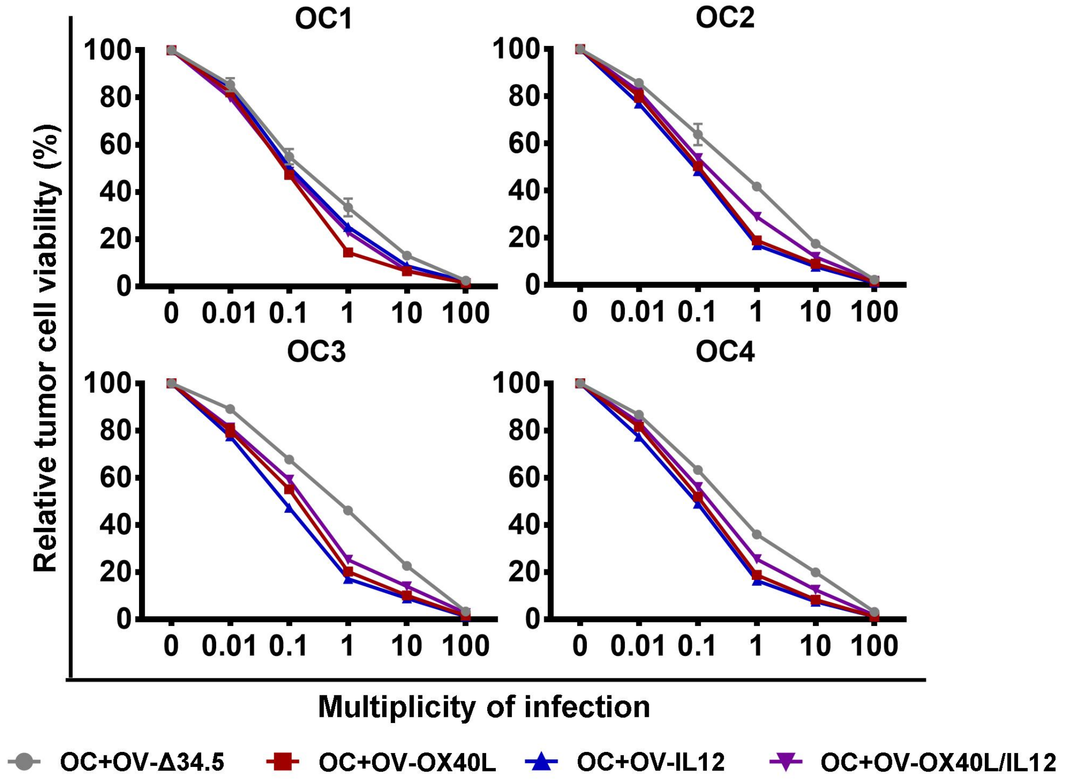

Figure S2. Susceptibility of primary cancer cells to different modified OVs.

Primary oral cancer cells from 4 patients were infected with the indicated OVs at different MOIs. Cell viability was determined at 48 h after infection by using the MTT assay (n=3).

Supplementary Figure 3

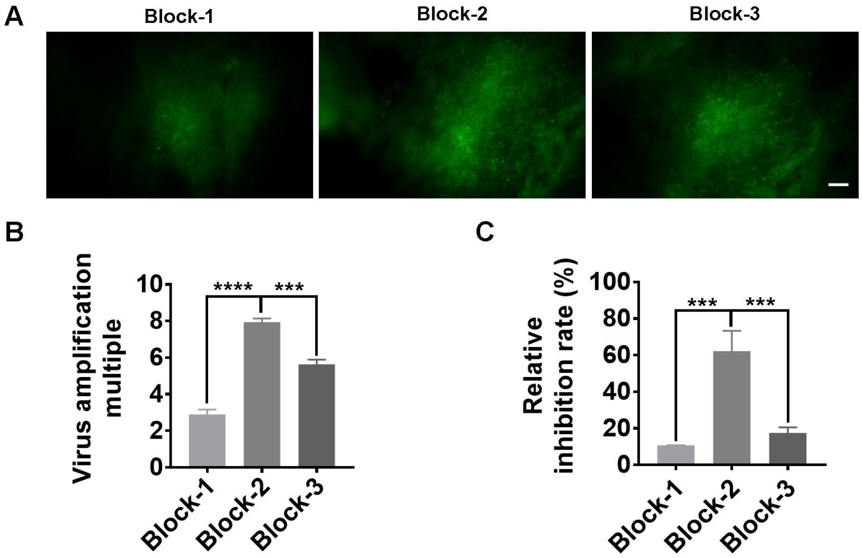

Figure S3. Susceptibility of primary oral cancer tissues to OV-GFP.

(A) Fluorescence images of 3 oral cancer tissue blocks that were infected with OV-GFP for 48 h (n=3). Scale bars, 200 μm. (B) The replication of viruses in these cancer tissues was determined by using the titration assay (n=3). (C) The viability of tumor tissues was determined with the alamar blue assay (n=3). The relative inhibition rate was calculated by dividing the alamar blue dye signal at 48 h after OV-GFP treatment by the dye signal before the addition of OV-GFP. The statistical analysis was determined by one-way ANOVA, followed by Tukey's multiple comparison test analysis. All values are presented as the mean ± SEM. ***$p < 0.001$, ****$p < 0.0001$.

70 **Supplementary Figure 4**

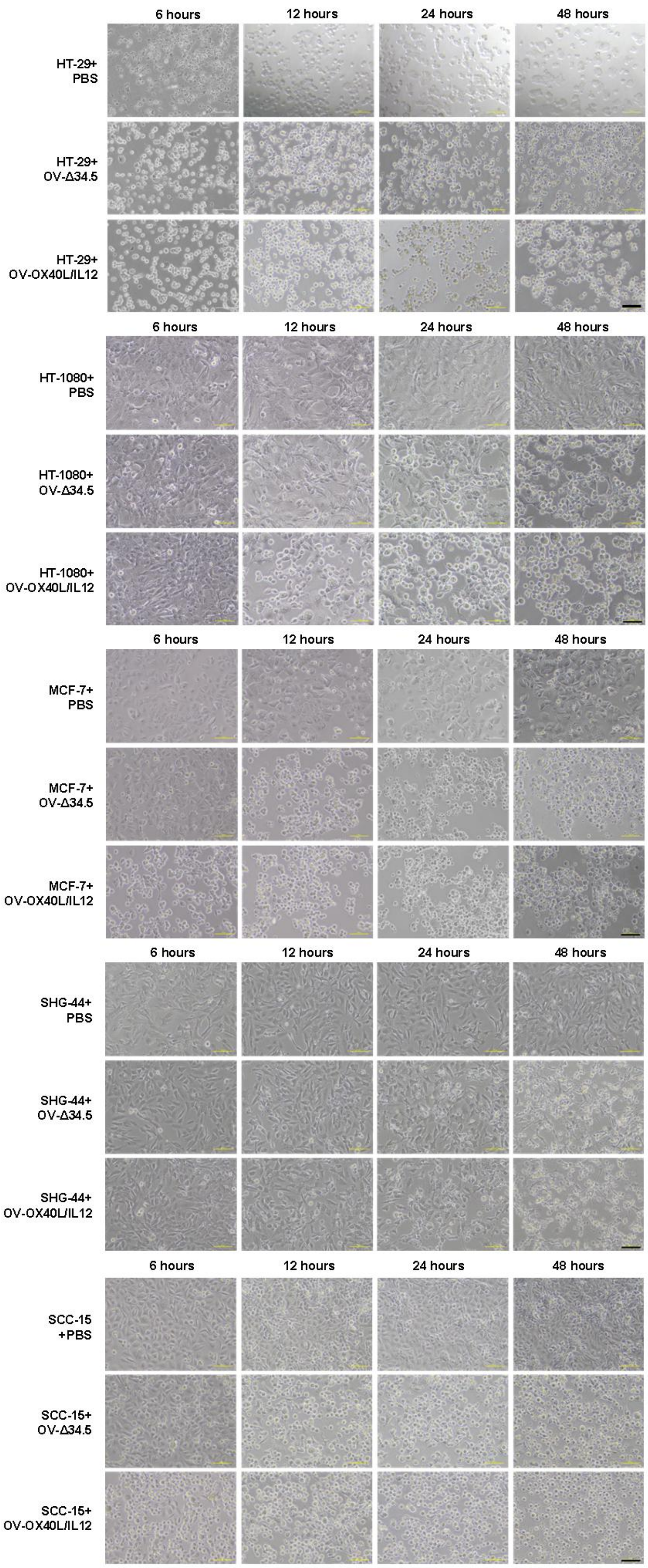

71

Figure S4. Susceptibility of primary oral cancer tissues to OV-Δ34.5 and OV-OX40L/IL12.

SHG-44, SCC-15, MCF-7, HT-1080 and HT-29 cells were cultured with OV-Δ34.5 or OV-OX40L/IL12 (MOI=0.01) for 6, 12, 24 and 48 h (n=3). Representative images showing CPEs are shown. Scale bars, 200 μm.

Supplementary Figure 5

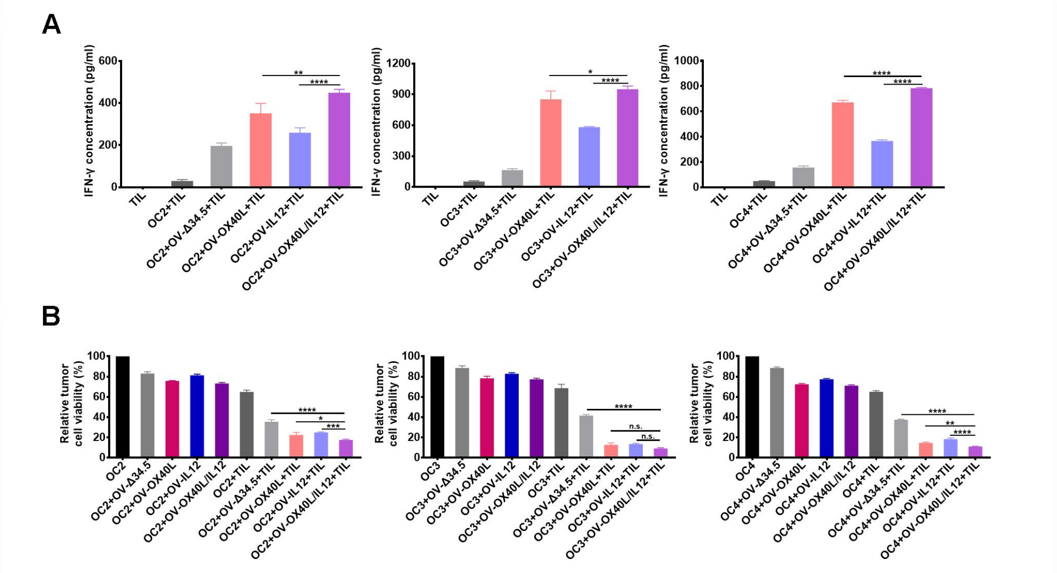

Figure S5. TILs were activated by OV-OX40L/IL12-infected tumor cells and exhibited enhanced tumoricidal activity.

Oral cancer cells from 3 patients numbered OC2, OC3 and OC4 were infected with the indicated OV and cocultured with TILs from the matched patients for 24 h. (A) IFN-γ levels in the culture supernatant were determined by using ELISA (n=3). (B) The viability of the tumor cells in coculture was assessed by using the MTT assay (n=3). Statistical significance was determined by using one-way ANOVA followed by multiple comparison test. All values are presented as the mean ± SEM. NS, not significant, *$p < 0.05$, **$p < 0.01$, ***$p < 0.001$ and ****$p < 0.0001$.

Supplementary Figure 6

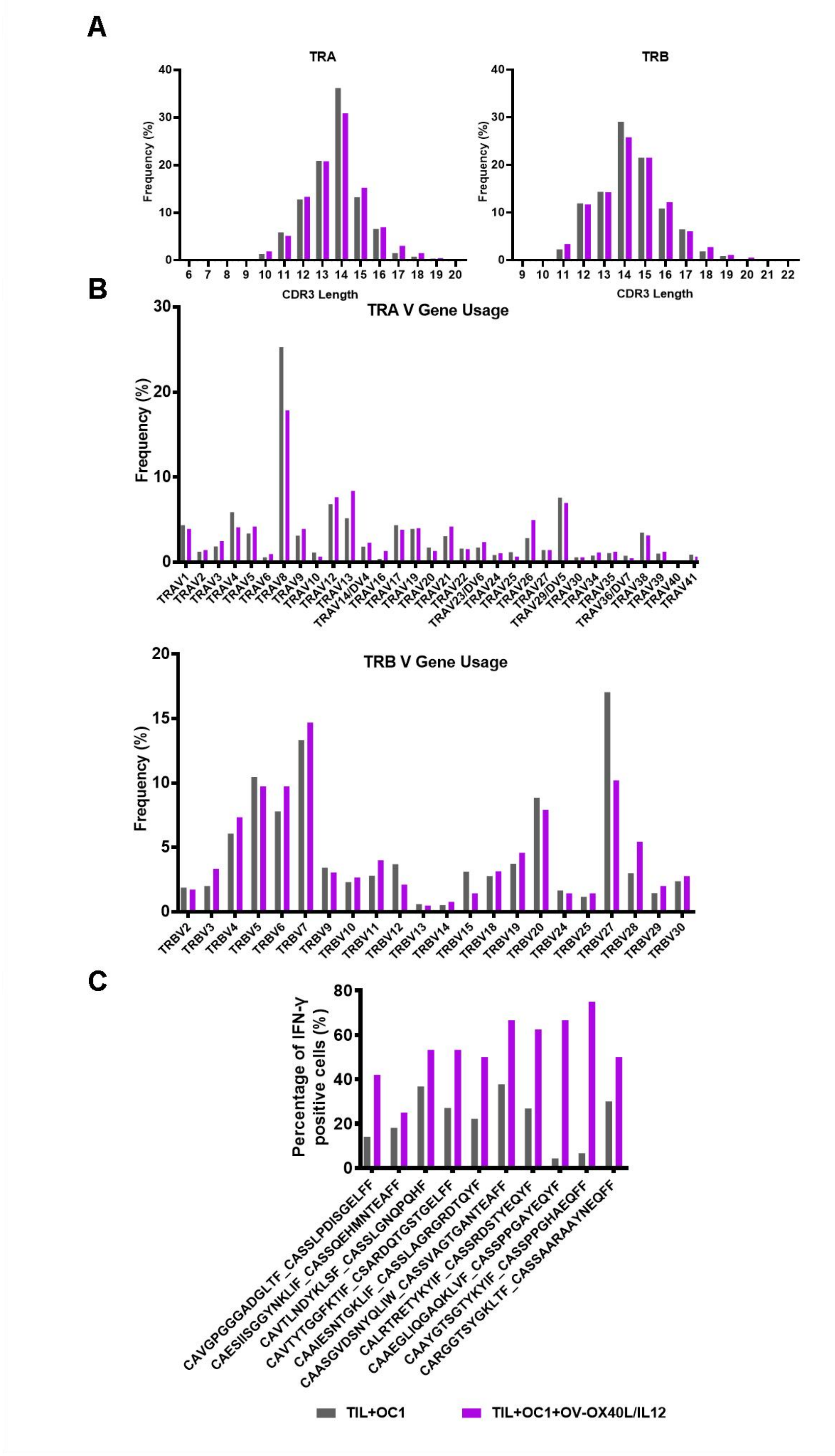

Figure S6. The length distribution of CDR3 or TRAV and TRBV germline usage for TCRs of TILs in coculture.

TILs were cocultured with mock-infected tumor cells or OV-OX40L/IL12-infected tumor cells. (A) The distribution of CDR3 length in both alpha and beta chains of TCRs. (B) TRAV and TRBV gene segment germline usage. (C) scTCR-seq and scRNA-seq were performed on TILs cocultured with OV-OX40L/IL12- or mock-infected tumor cells. The frequency of IFN-γ-positive T cells is shown for the 10 most prevalent TCR clonotypes of TILs (n=3).

Supplementary Figure 7

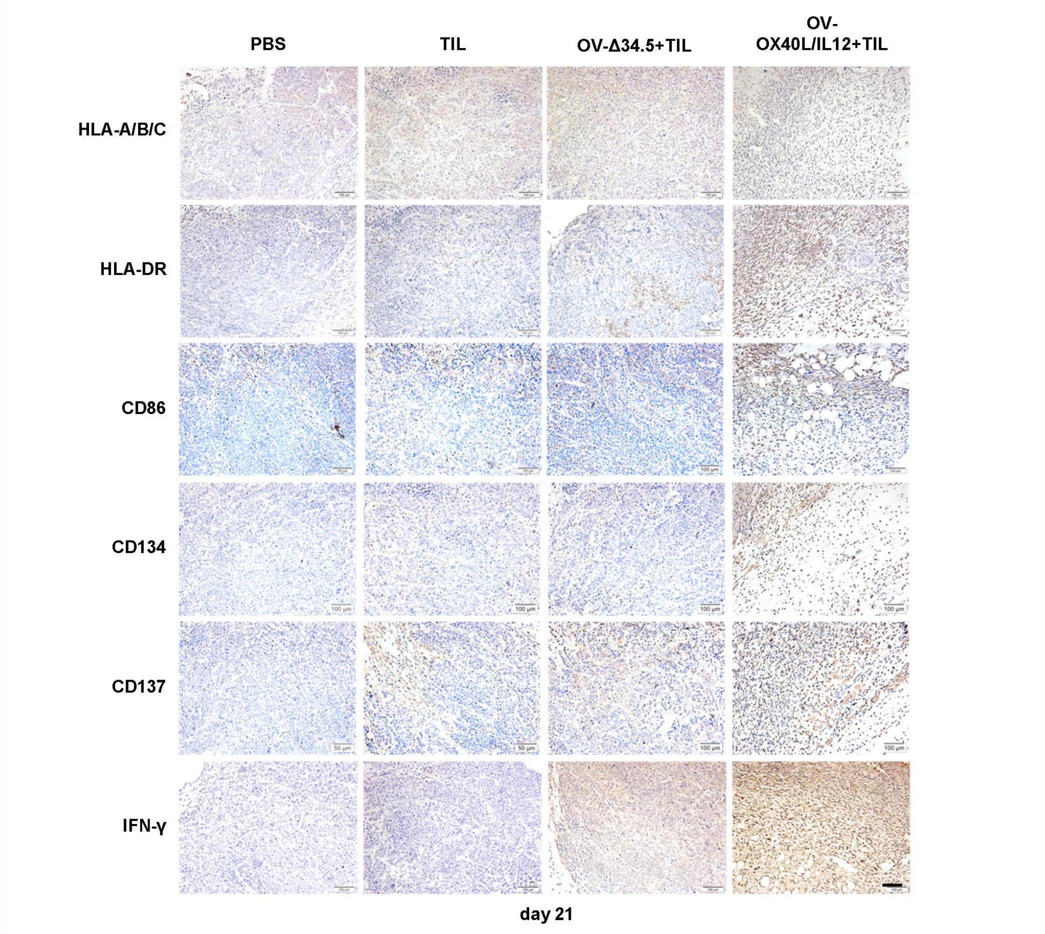

Figure S7. Representative images of IHC staining for HLA-ABC, HLA-DR, CD86, CD134, CD137 and IFN-γ in tumor tissue sections 21 days after treatment of the OC1 PDX model (n=3). Scale bars, 100 μm.

Supplementary Figure 8

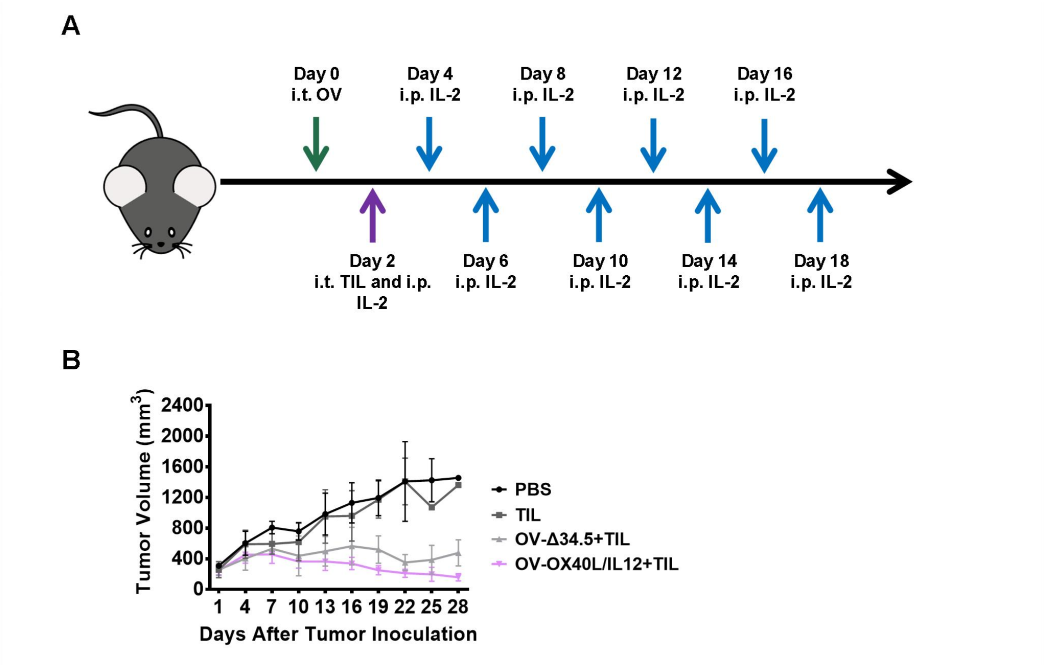

Figure S8. Antitumor efficacy of the combination therapy with OV-OX40L/IL12 and TILs in the 4th PDX tumor model (OC4 PDX model). (A) NSG mice were subcutaneously implanted with patient-derived tumor tissues, and the established tumors were intratumorally injected with the indicated OV followed by an intratumor injection of *ex vivo* expanded TILs from the same patient numbered OC4 (n=4). Tumor growth was measured every other day. The average tumor volumes (B) are shown.

Supplementary Figure 9

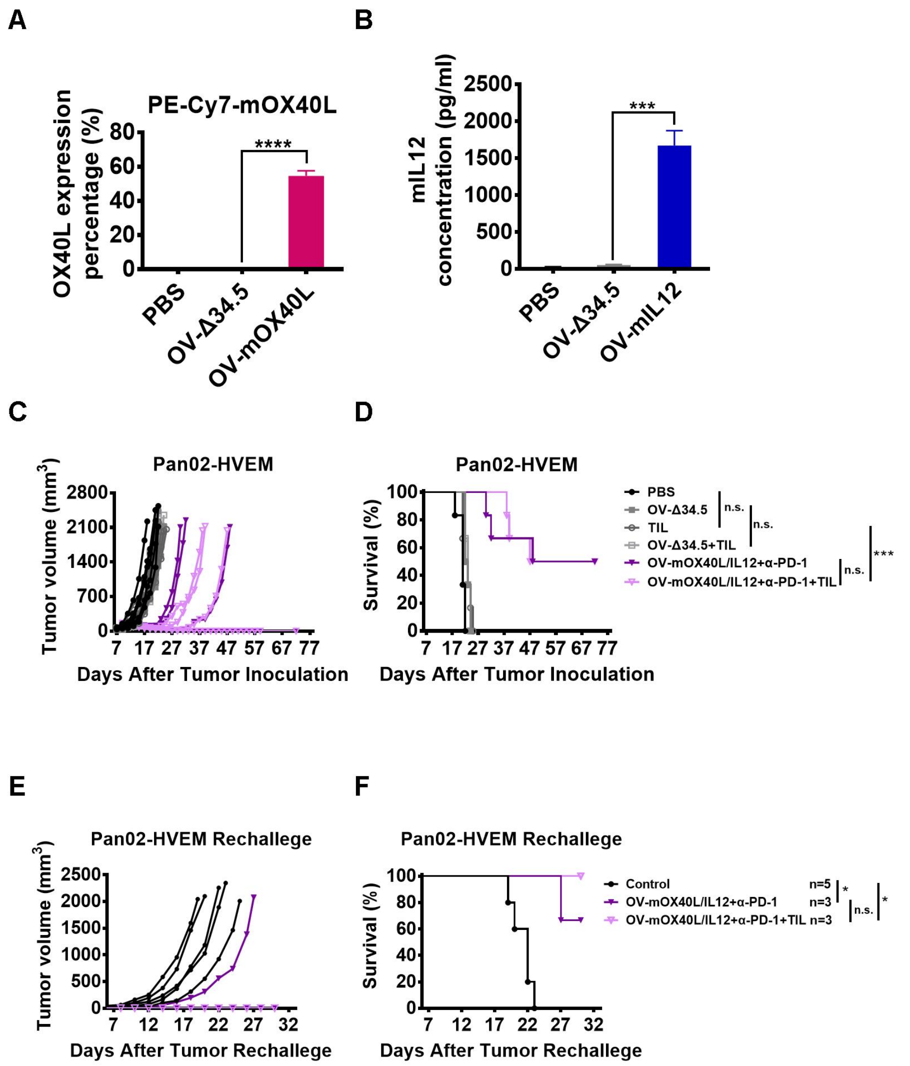

Figure S9. Combination therapy with OV-mOX40L/IL12 and TILs led to marked tumor regression in immunocompetent murine tumor models.

(A) The expression of functional mOX40L by the infected MC38 tumor cells was analyzed (n=3) by using flow cytometry. (B) Secretion of mIL12 from the infected MC38 cells was detected by using ELISA (n=3). The statistical analysis was determined by one-way ANOVA, followed by Tukey's multiple comparison test analysis. ***p < 0.001, ****p < 0.0001. (C) Tumor growth was monitored over time using electric calipers to measure tumor size in two dimensions (n=6). (D) The survival curve of Pan02-HVEM tumor-bearing mice was plotted using Kaplan-Meier analysis, and the log-rank test indicated a significant difference in the survival curves between the OV-mOX40L/IL12, PD-1 antagonist antibody and TIL combination therapy groups and the OV-Δ34.5+TIL group (n=6). Mice previously cured of Pan02-HVEM tumors by the combination therapy and age-matched treatment-naïve mice were subcutaneously inoculated with Pan02-HVEM cells. Tumor growth (E) in individual mice and the survival curve (F) are shown. All values are presented as the mean ± SEM. NS, not significant, *p < 0.05 and ***p < 0.001.

Supplementary Figure 10

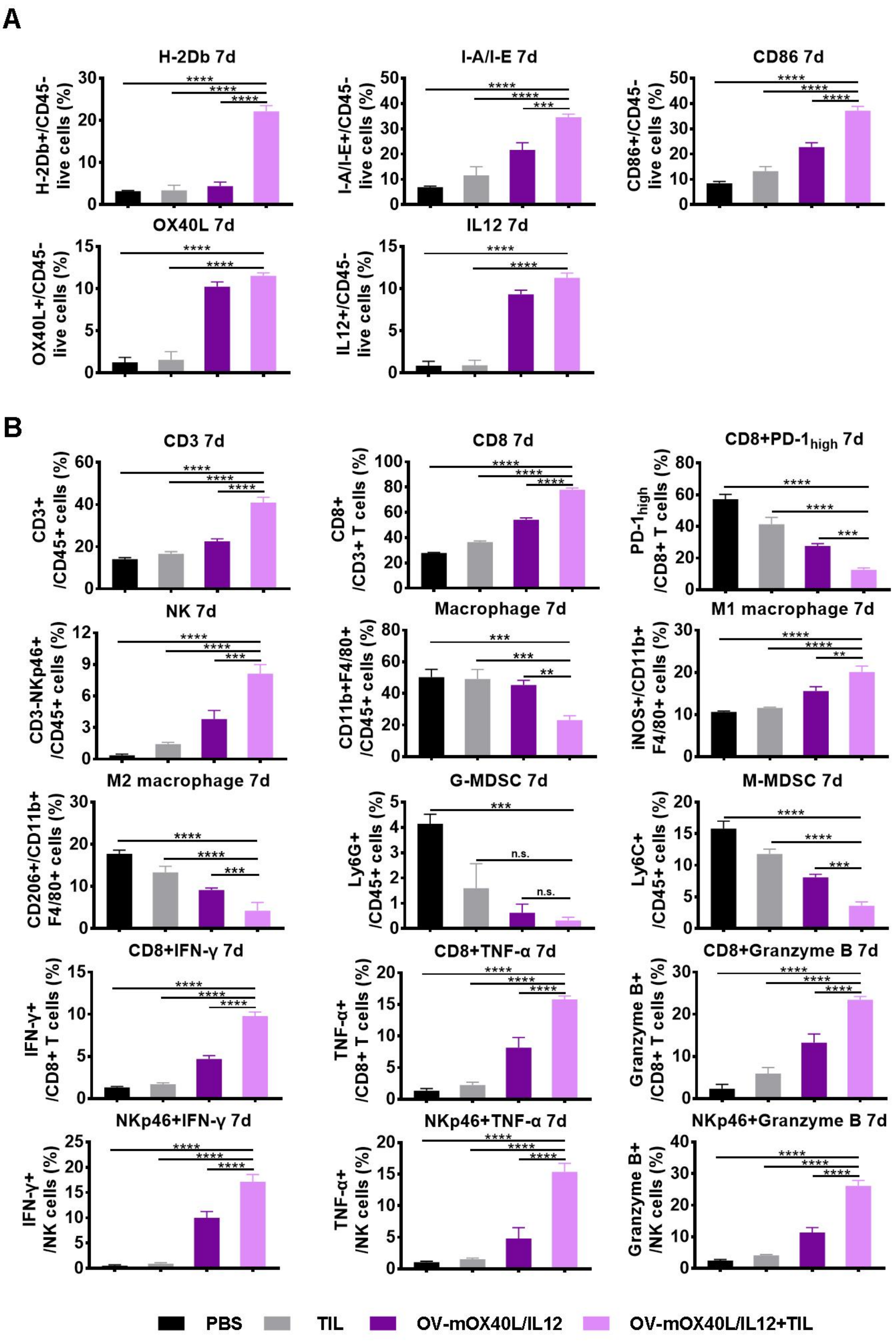

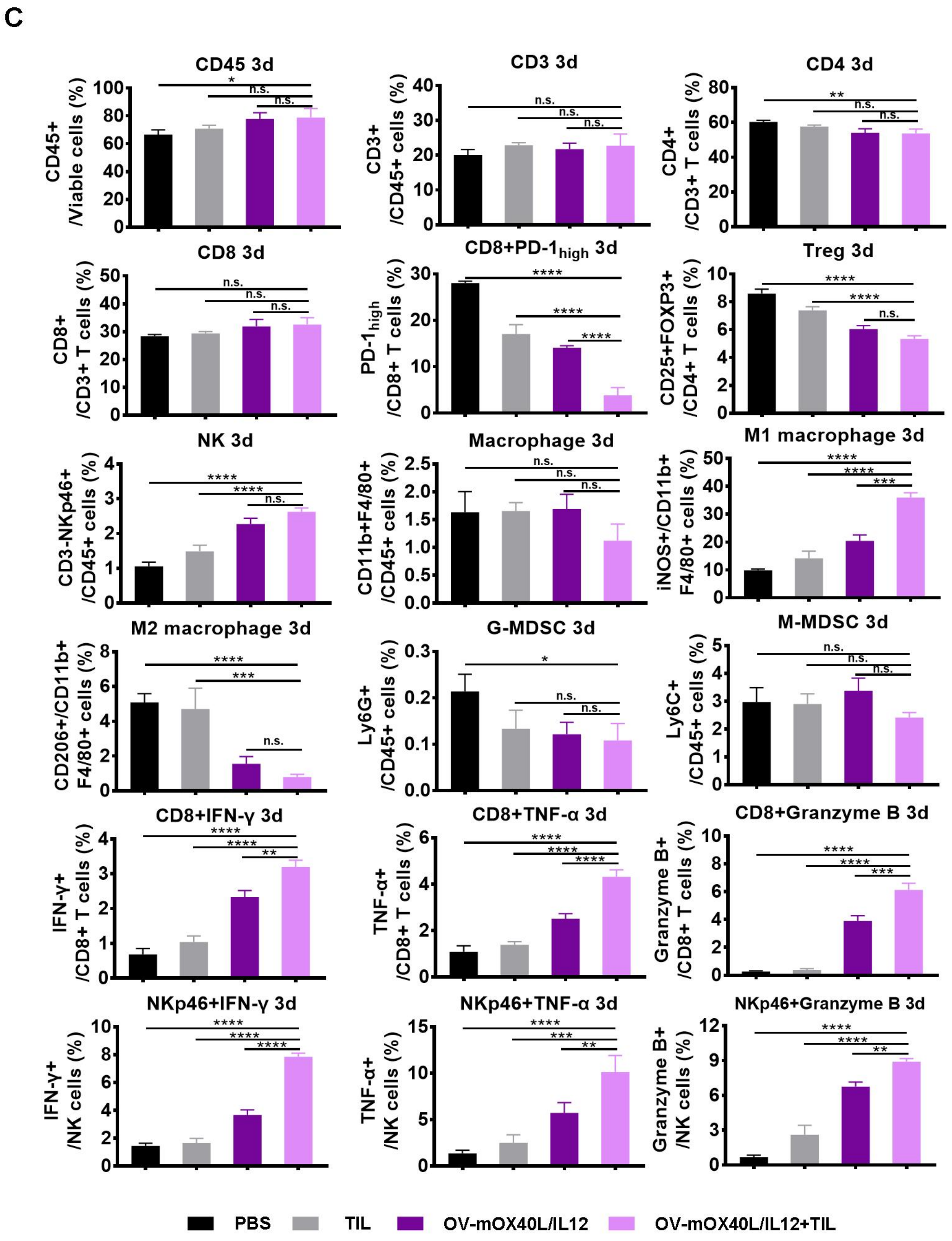
C
CD45 3d
CD45+ /Viable cells (%)
CD3 3d
CD3+ /CD45+ cells (%)
CD4 3d
CD4+ /CD3+ T cells (%)
CD8 3d
CD8+ /CD3+ T cells (%)
CD8+PD-1high 3d
PD-1high /CD8+ T cells (%)
Treg 3d
CD25+FOXP3+ /CD4+ T cells (%)
NK 3d
CD3-NKp46+ /CD45+ cells (%)
Macrophage 3d
CD11b+F4/80+ /CD45+ cells (%)
M1 macrophage 3d
iNOS+/CD11b+ F4/80+ cells (%)
M2 macrophage 3d
CD206+/CD11b+ F4/80+ cells (%)
G-MDSC 3d
Ly6G+ /CD45+ cells (%)
M-MDSC 3d
Ly6C+ /CD45+ cells (%)
CD8+IFN-γ 3d
IFN-γ+ /CD8+ T cells (%)
CD8+TNF-α 3d
TNF-α+ /CD8+ T cells (%)
CD8+Granzyme B 3d
Granzyme B+ /CD8+ T cells (%)
NKp46+IFN-γ 3d
IFN-γ+ /NK cells (%)
NKp46+TNF-α 3d
TNF-α+ /NK cells (%)
NKp46+Granzyme B 3d
Granzyme B+ /NK cells (%)
PBS
TIL
OV-mOX40L/IL12
OV-mOX40L/IL12+TIL

D

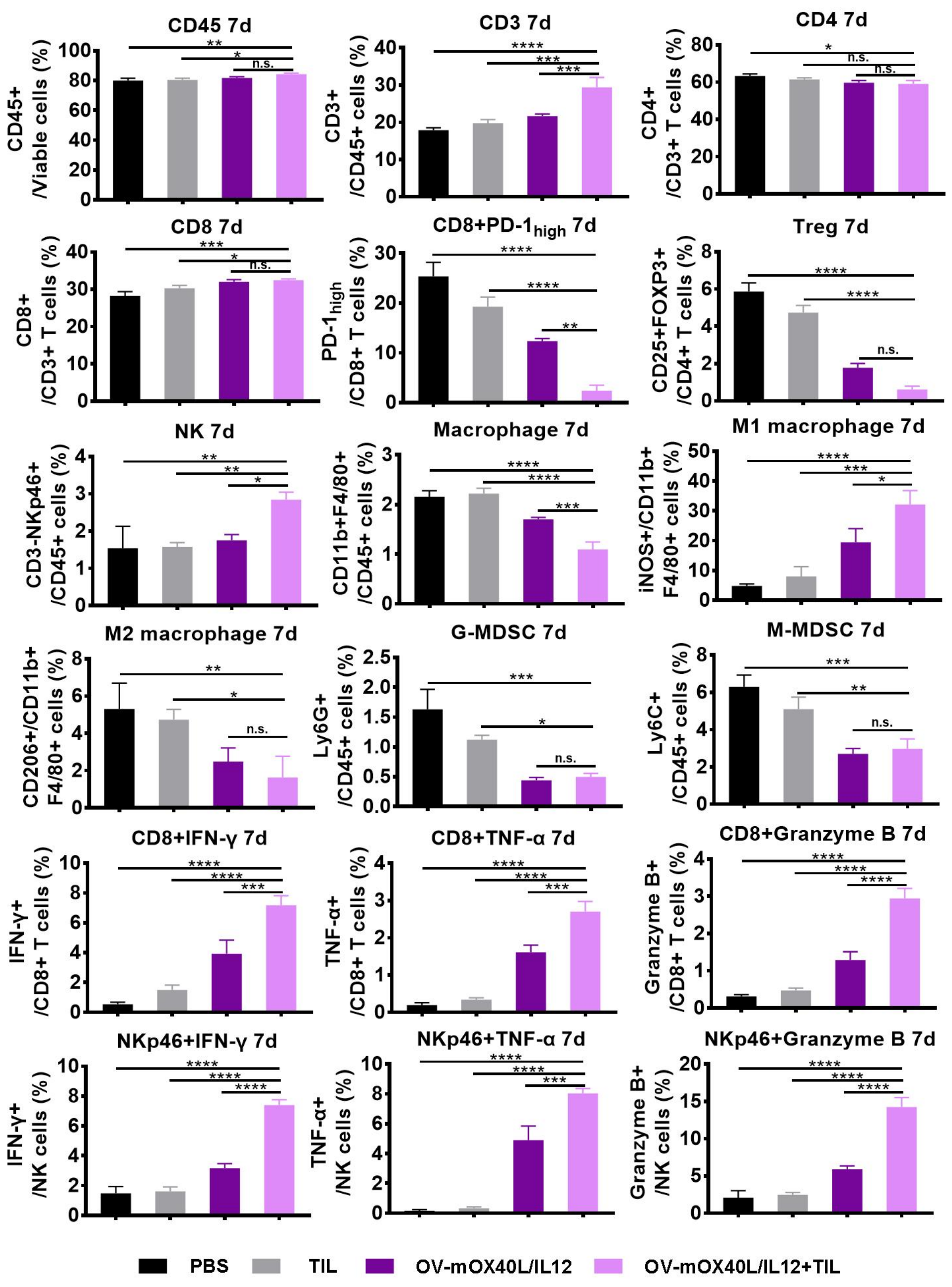
CD45 7d
CD3 7d
CD4 7d
CD8 7d
CD8+PD-1high 7d
Treg 7d
NK 7d
Macrophage 7d
M1 macrophage 7d
M2 macrophage 7d
G-MDSC 7d
M-MDSC 7d
CD8+IFN-γ 7d
CD8+TNF-α 7d
CD8+Granzyme B 7d
NKp46+IFN-γ 7d
NKp46+TNF-α 7d
NKp46+Granzyme B 7d
PBS
TIL
OV-mOX40L/IL12
OV-mOX40L/IL12+TIL

208

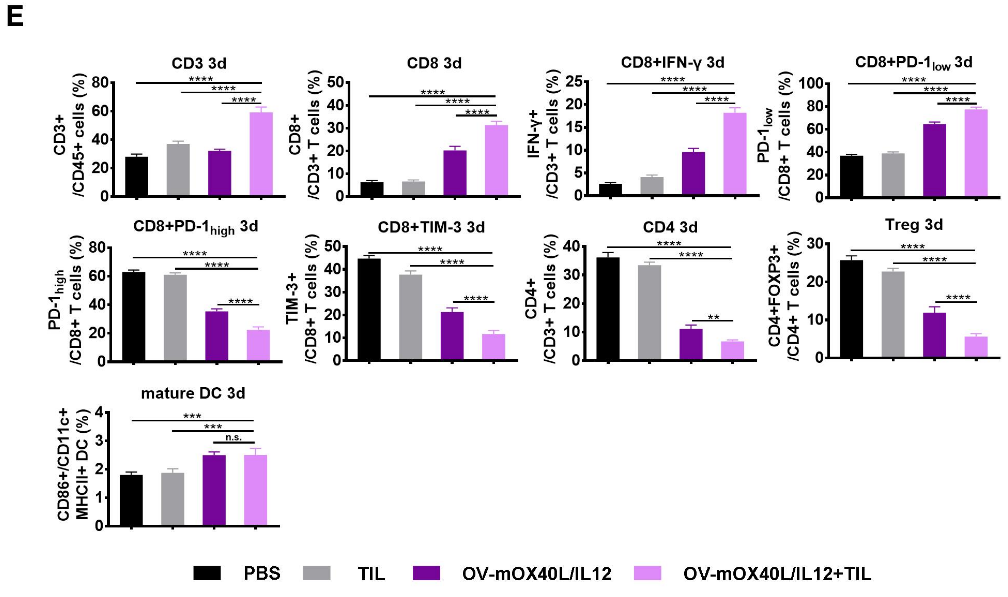
E
CD3 3d
CD3+ /CD45+ cells (%)
CD8 3d
CD8+ /CD3+ T cells (%)
CD8+IFN-γ 3d
IFN-γ+ /CD3+ T cells (%)
CD8+PD-1low 3d
PD-1low /CD8+ T cells (%)
CD8+PD-1high 3d
PD-1high /CD8+ T cells (%)
CD8+TIM-3 3d
TIM-3+ /CD8+ T cells (%)
CD4 3d
CD4+ /CD3+ T cells (%)
Treg 3d
CD4+FOXP3+ /CD4+ T cells (%)
mature DC 3d
CD86+/CD11c+ MHCII+ DC (%)
PBS
TIL
OV-mOX40L/IL12
OV-mOX40L/IL12+TIL

Supplementary Figure 10. Analysis of tumors, tdLNs and splenocytes after MC38-tumor-bearing mice were treated with the OV and TILs.

Mice were subcutaneously implanted with MC38 cells and treated as indicated (n=3). A single-cell suspension was prepared from tumor tissue 7 days after treatment. Tumor cells (A) and tumor infiltrating immune cells (B) were stained and subjected to flow cytometry analysis of the expression of APC-related molecules (A) and profiling of different types of immune cells and their activation status (B). A single-cell suspension was prepared from spleen tissue 3 days (C) and 7 days (D) after treatment. Splenocytes were stained and subjected to a flow cytometry analysis. (E) Flow cytometry analysis of endogenous DCs and multiple immune cell phenotypes in the tdLNs. The statistical analysis was determined by one-way ANOVA, followed by Tukey's multiple comparison test analysis. All values are presented as the mean ± SEM. NS, not significant, *p < 0.05, **p < 0.01, ***p < 0.001 and ****p < 0.0001.

Supplementary Figure 11

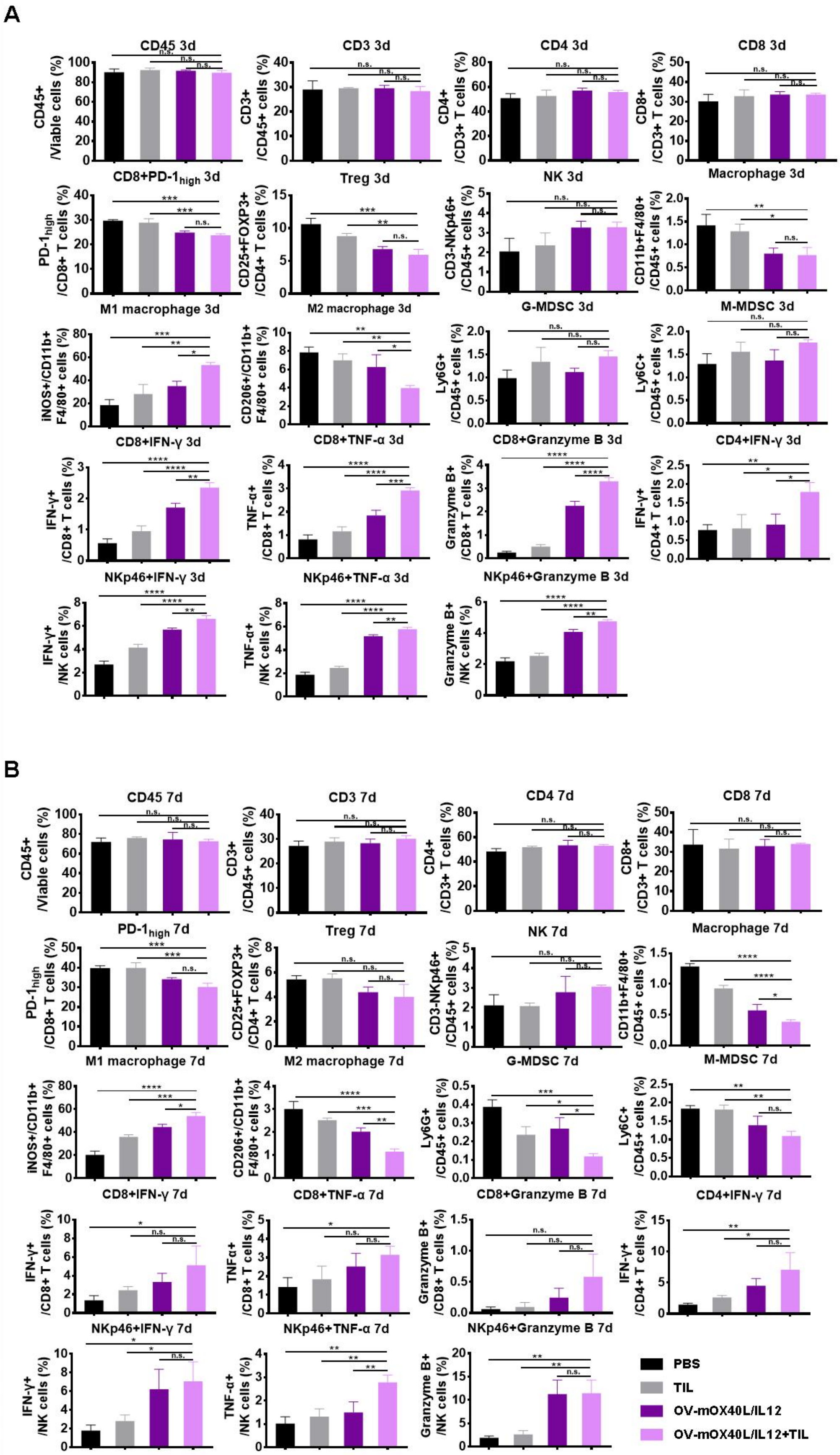

C

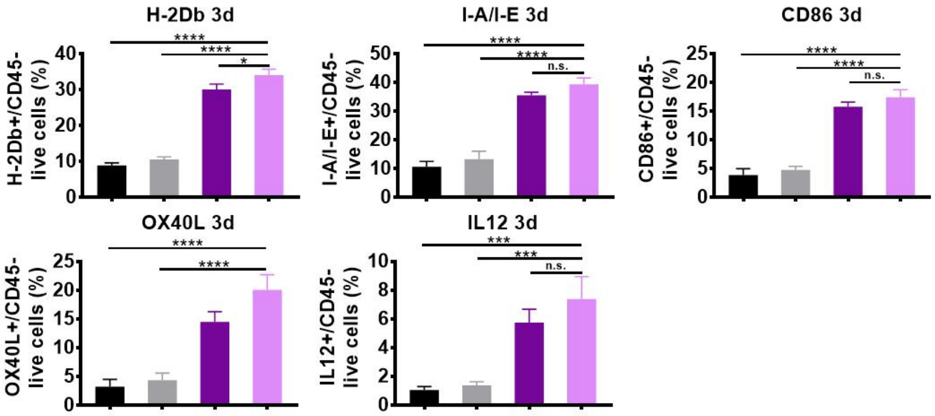
H-2Db 3d
H-2Db+/CD45- live cells (%)
I-A/I-E 3d
I-A/I-E+/CD45- live cells (%)
CD86 3d
CD86+/CD45- live cells (%)
OX40L 3d
OX40L+/CD45- live cells (%)
IL12 3d
IL12+/CD45- live cells (%)
n.s.

D

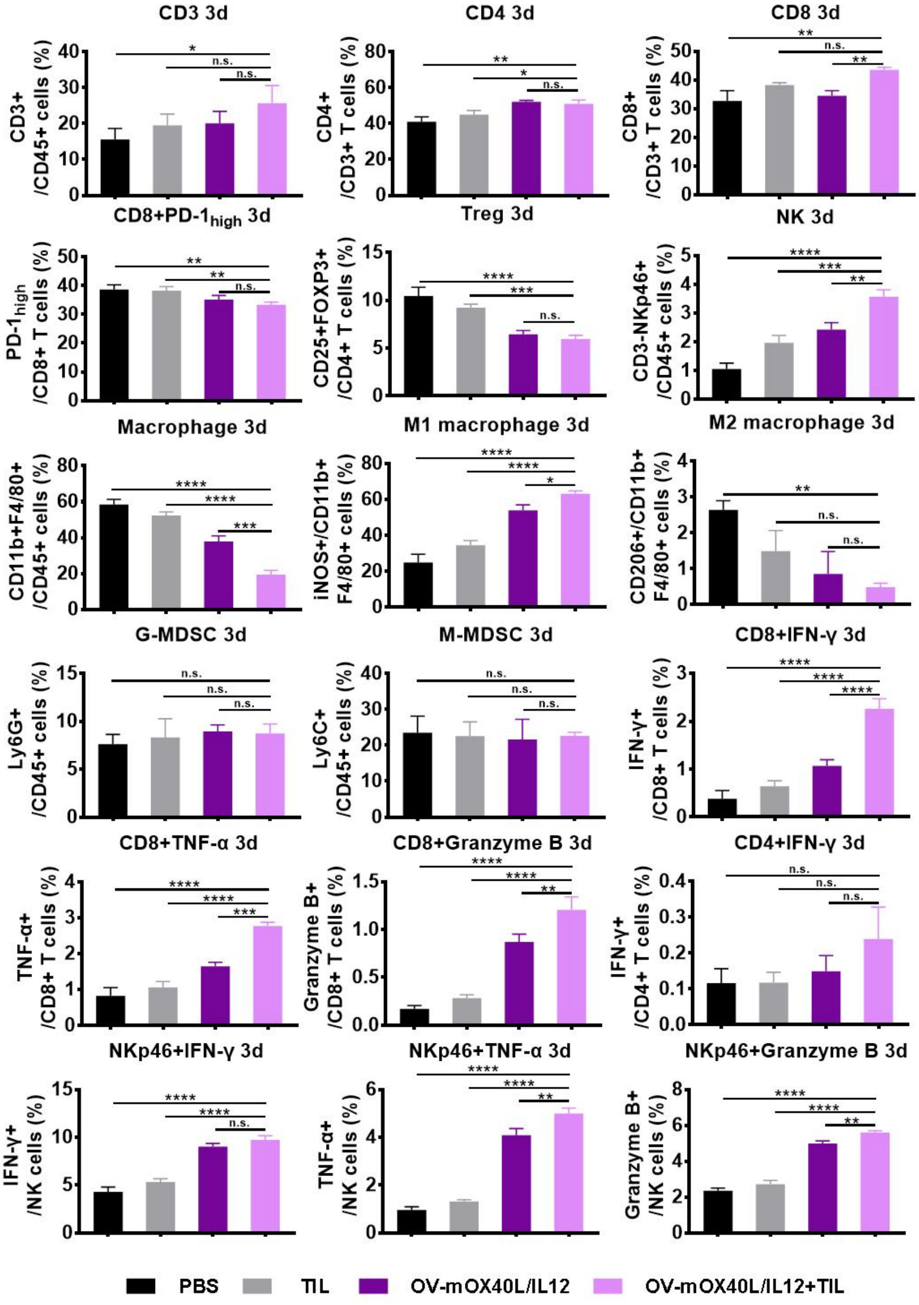
CD3 3d
CD3+ /CD45+ cells (%)
CD4 3d
CD4+ /CD3+ T cells (%)
CD8 3d
CD8+ /CD3+ T cells (%)
CD8+PD-1high 3d
PD-1high /CD8+ T cells (%)
Treg 3d
CD25+FOXP3+ /CD4+ T cells (%)
NK 3d
CD3-NKp46+ /CD45+ cells (%)
Macrophage 3d
CD11b+F4/80+ /CD45+ cells (%)
M1 macrophage 3d
iNOS+/CD11b+ F4/80+ cells (%)
M2 macrophage 3d
CD206+/CD11b+ F4/80+ cells (%)
G-MDSC 3d
Ly6G+ /CD45+ cells (%)
M-MDSC 3d
Ly6C+ /CD45+ cells (%)
CD8+IFN-γ 3d
IFN-γ+ /CD8+ T cells (%)
CD8+TNF-α 3d
TNF-α+ /CD8+ T cells (%)
CD8+Granzyme B 3d
Granzyme B+ /CD8+ T cells (%)
CD4+IFN-γ 3d
IFN-γ+ /CD4+ T cells (%)
NKp46+IFN-γ 3d
IFN-γ+ /NK cells (%)
NKp46+TNF-α 3d
TNF-α+ /NK cells (%)
NKp46+Granzyme B 3d
Granzyme B+ /NK cells (%)
n.s.
PBS
TIL
OV-mOX40L/IL12
OV-mOX40L/IL12+TIL

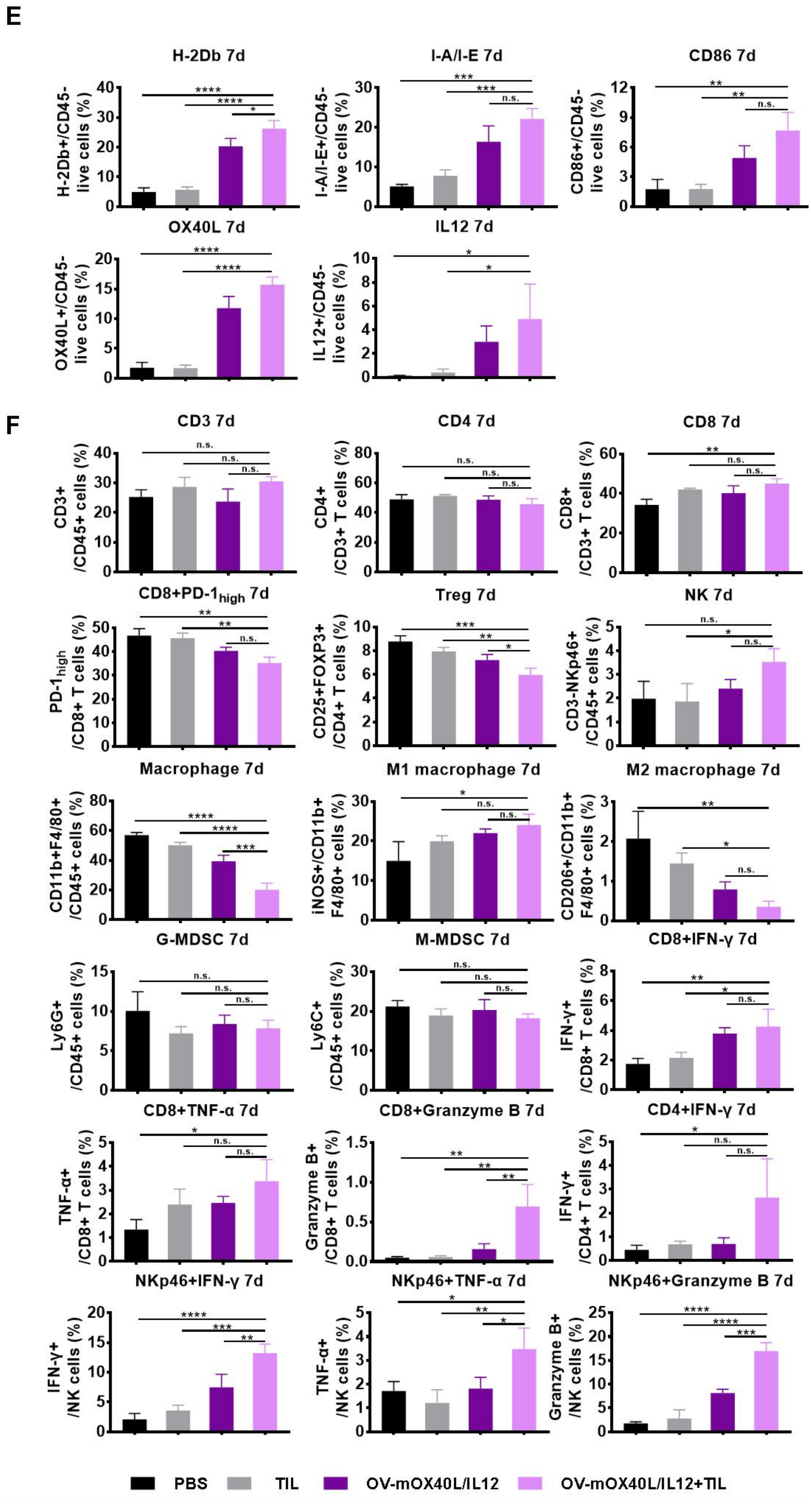
E
H-2Db 7d
H-2Db+/CD45- live cells (%)
I-A/I-E 7d
I-A/I-E+/CD45- live cells (%)
CD86 7d
CD86+/CD45- live cells (%)
OX40L 7d
OX40L+/CD45- live cells (%)
IL12 7d
IL12+/CD45- live cells (%)
F
CD3 7d
CD3+ /CD45+ cells (%)
CD4 7d
CD4+ /CD3+ T cells (%)
CD8 7d
CD8+ /CD3+ T cells (%)
CD8+PD-1high 7d
PD-1high /CD8+ T cells (%)
Treg 7d
CD25+FOXP3+ /CD4+ T cells (%)
NK 7d
CD3-NKp46+ /CD45+ cells (%)
Macrophage 7d
CD11b+F4/80+ /CD45+ cells (%)
M1 macrophage 7d
iNOS+/CD11b+ F4/80+ cells (%)
M2 macrophage 7d
CD206+/CD11b+ F4/80+ cells (%)
G-MDSC 7d
Ly6G+ /CD45+ cells (%)
M-MDSC 7d
Ly6C+ /CD45+ cells (%)
CD8+IFN-γ 7d
IFN-γ+ /CD8+ T cells (%)
CD8+TNF-α 7d
TNF-α+ /CD8+ T cells (%)
CD8+Granzyme B 7d
Granzyme B+ /CD8+ T cells (%)
CD4+IFN-γ 7d
IFN-γ+ /CD4+ T cells (%)
NKp46+IFN-γ 7d
IFN-γ+ /NK cells (%)
NKp46+TNF-α 7d
TNF-α+ /NK cells (%)
NKp46+Granzyme B 7d
Granzyme B+ /NK cells (%)
PBS
TIL
OV-mOX40L/IL12
OV-mOX40L/IL12+TIL

Figure S11. Analysis of tumors and splenocytes after Pan02-HVEM tumor-bearing mice were treated with the OV and TILs.

Mice were subcutaneously implanted with Pan02-HVEM cells and treated as indicated (n=3). A single-cell suspension was prepared from the spleen tissue 3 days (A) and 7 days (B) after treatment. Splenocytes were stained and subjected to a flow cytometry analysis. A single-cell suspension was prepared from tumor tissues 3 days (C, D) and 7 days (E, F) after treatment. Tumor cells (C, E) and tumor-infiltrating mononuclear cells (D, F) were stained and subjected to a flow cytometry analysis to determine the expression of APC-related molecules (C, E), and the profiles of different types of immune cells and their activation status are presented (D, F). The statistical analysis was determined by one-way ANOVA, followed by Tukey's multiple comparison test analysis. All values are presented as the mean ± SEM. NS, not significant, *$p < 0.05$, **$p < 0.01$, ***$p < 0.001$ and ****$p < 0.0001$.

Supplementary Figure 12

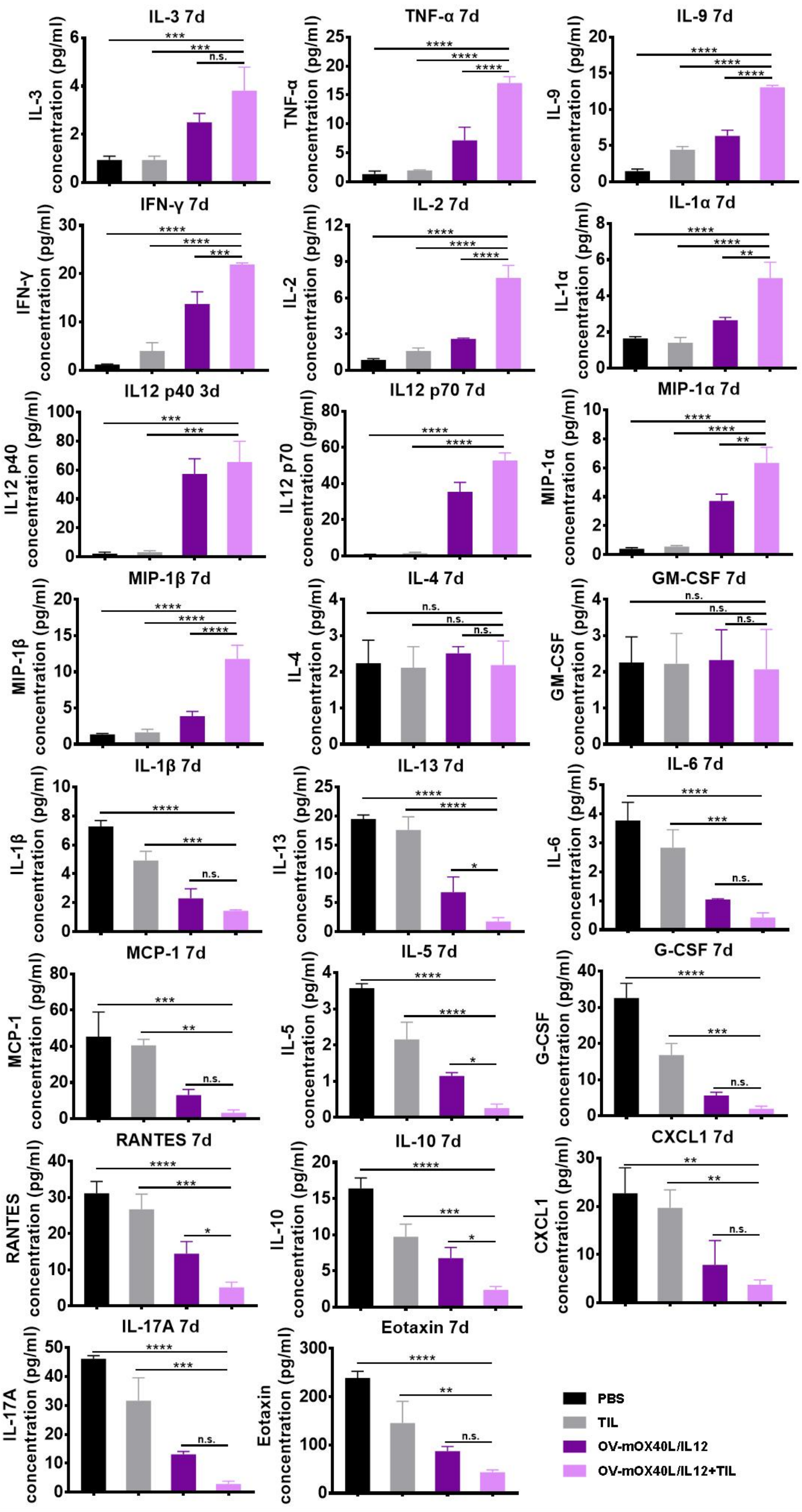

Figure S12. Profiles of cytokines and chemokines in serum samples from treated mice.

Mice were subcutaneously implanted with MC38 cells and treated as indicated (n=3). Cytokine concentrations in serum collected 7 days after treatment were determined using a Bio-Plex Mouse Cytokine 23-Plex Array. The statistical analysis was determined by one-way ANOVA, followed by Tukey's multiple comparison test analysis. All values are presented as the mean ± SEM. NS, not significant, $*p < 0.05$, $**p < 0.01$, $***p < 0.001$ and $****p < 0.0001$.

Supplementary Figure 13

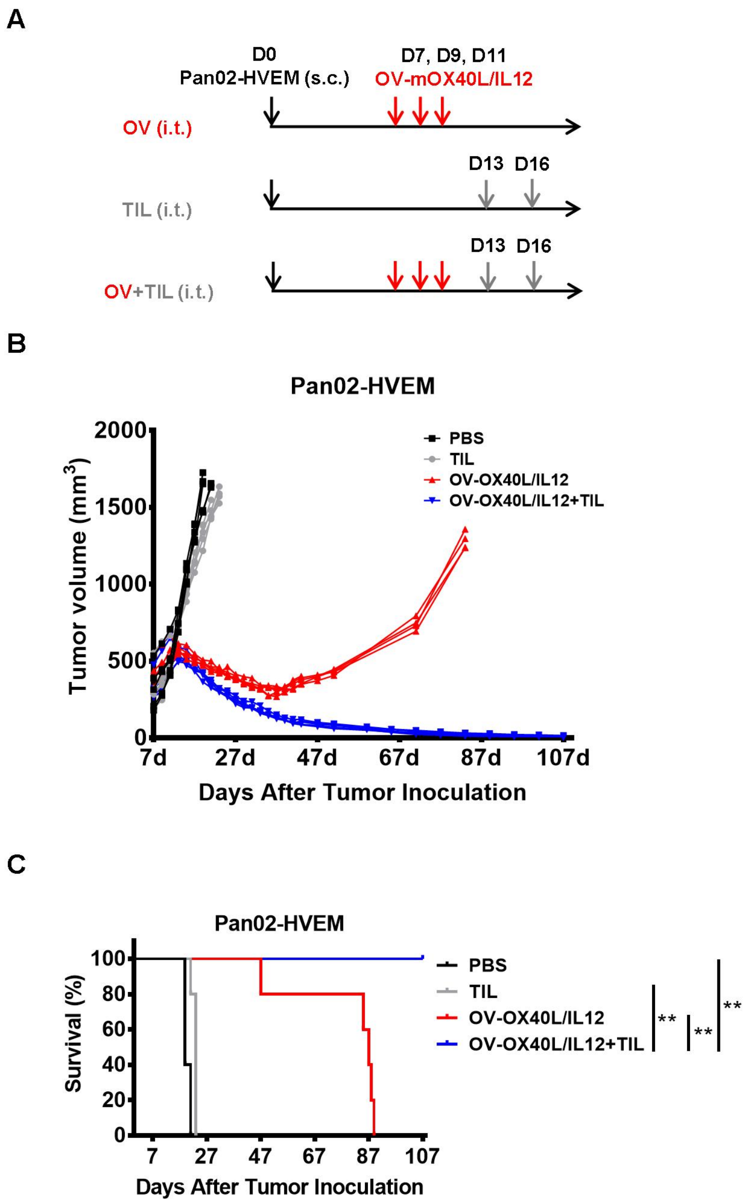

Figure S13. Tumor growth and survival of Pan02-HVEM tumor-bearing mice after treatment.

(A) Schematic of C57BL/6J mice with subcutaneous Pan02-HVEM tumors treated with OV-mOX40L/IL12 and TILs. (B) Mice were subcutaneously injected with Pan02-HVEM cells (1×10^6 cells) on day 0. On days 7, 9 and 11, mice were intratumorally treated with 0 or three doses of 2×10^6 PFUs of OV-mOX40L/IL12 per mouse. On days 13 and 16, the mice were administered intratumoral injections of two doses of TILs (1×10^6 cells). The tumor volume was measured with calipers. Data for each mouse (n = 5 mice per group) are shown. (C) Kaplan–Meier survival curves for the experiment described in (A). The p value indicates the difference between OV-mOX40L/IL12 and OV-mOX40L/IL12+TIL, as determined by using the log rank (Mantel–Cox) test. **$p < 0.01$.

Supplementary Figure 14

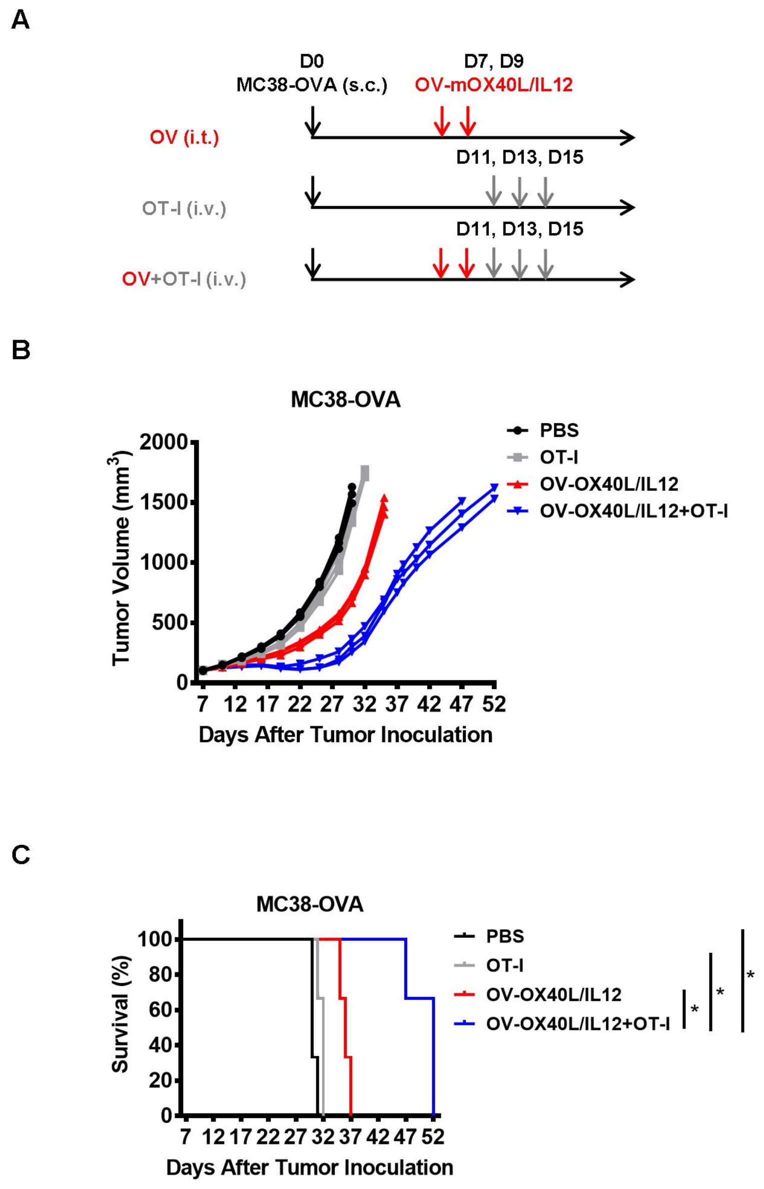

Figure S14. The antitumor effect of intratumoral administration of OV-OX40L/IL12 plus systemic administration of TILs.

(A) Schematic of C57BL/6J mice with subcutaneous MC38-OVA tumors treated with OV-mOX40L/IL12 and OT-I T cells. Mice were injected subcutaneously with MC38-OVA cells on day 0. On days 7 and 9, mice were subcutaneously sham injected or were injected with 2×10^{6} PFUs of OV-mOX40L/IL12 per mouse. On days 11, 13 and 15, the mice were injected with PBS or OT-I T cells (1×10^{7} cells) via the tail vein. n = 3 mice per group. (B) Growth of transplanted tumors in the MC38-OVA model (n = 3). (C) Kaplan–Meier survival curves for the experiment described in (A). The mean ± SEM are shown. The p value indicates the difference between OV-mOX40L/IL12 and OV-mOX40L/IL12+OT-I, as determined by using the log rank (Mantel–Cox) test. *$p < 0.05$.

Supplementary Figure 15

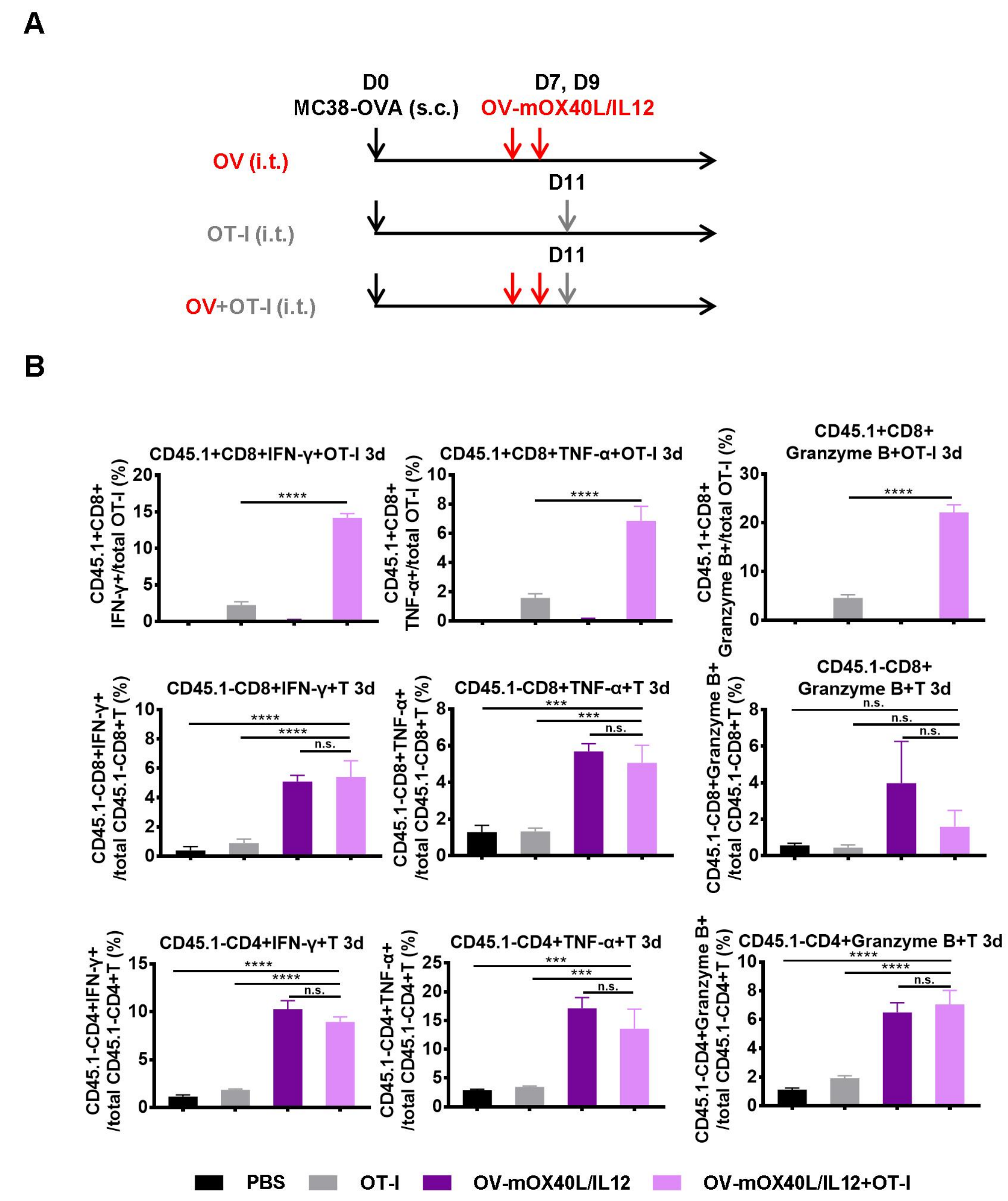

Figure S15. Effect of OV-mOX40L/IL12 on the activation of adoptively transferred and endogenous T cells.

(A) Schematic of C57BL/6J mice subcutaneous MC38-OVA tumors treated with OV-mOX40L/IL12 and OT-I T cells. (B) A single-cell suspension was prepared from tumor tissues 3 days after treatment. TILs were stained and subjected to a flow cytometry analysis to profile different types of immune cells and their activation status. The statistical analysis was determined by one-way ANOVA, followed by Tukey's multiple comparison test analysis. All values are presented as the mean ± SEM. NS, not significant, $*p < 0.05$, $**p < 0.01$, $***p < 0.001$ and $****p < 0.0001$.

Supplementary Tables

Table S1. List of anti-human antibodies

Item	Vendor	Catalog number
PE anti-human CD252 (OX40L) Antibody	Biolegend	326307
PE/Dazzle™ 594 anti-human HLA-A, B, C Antibody	Biolegend	311439
FITC anti-human HLA-DR, DP, DQ Antibody	Biolegend	361705
PE anti-human CD80 Antibody	Biolegend	305207
FITC anti-human CD86 Antibody	Biolegend	374203
PE/Dazzle™ 594 anti-human CD274 (PD-L1) Antibody	Biolegend	329731
PE anti-human CD197 (CCR7) Antibody	Biolegend	353203
FITC anti-human CD45RA Antibody	Biolegend	304106
Anti-HLA-DR Antibody, Rabbit Polyclonal	Sino Biological	100586-T08
Anti-HLA A, B, C Antibody, Rabbit Polyclonal	Bioss	bs-10634R
Anti-CD86 Antibody, Rabbit Polyclonal	Bioss	bs-1035R
Anti-IFN gamma Antibody, Rabbit Polyclonal	Bioss	bs-0481R
Anti-CD137 Antibody, Rabbit Polyclonal	Bioss	bs-2449R
Anti-CD134 Antibody, Rabbit Polyclonal	Bioss	bs-2685R

Table S2. List of anti-mouse antibodies

Item	Vendor	Catalog number
FITC anti-mouse CD45 Antibody	Biolegend	103108
PerCP/Cyanine5.5 anti-mouse CD3ε Antibody	Biolegend	100328
Pacific Blue™ anti-mouse CD4 Antibody	Biolegend	100428
Brilliant Violet 510™ anti-mouse CD8a Antibody	Biolegend	100752
Brilliant Violet 605™ anti-mouse CD25 Antibody	Biolegend	102036
PE/Cyanine7 anti-mouse CD335 (NKp46) Antibody	Biolegend	137618
PE/Dazzle™ 594 anti-mouse CD279 (PD-1) Antibody	Biolegend	135228
Alexa Fluor® 647 anti-mouse/rat/human FOXP3 Antibody	Biolegend	320014
PE anti-mouse/human Ki-67 Antibody	Biolegend	151210
PerCP/Cyanine5.5 anti-mouse CD19 Antibody	Biolegend	152406
Pacific Blue™ anti-mouse/human CD11b Antibody	Biolegend	101224
Brilliant Violet 510™ anti-mouse Ly-6C Antibody	Biolegend	128033
Brilliant Violet 605™ anti-mouse Ly-6G Antibody	Biolegend	127639
PE/Cyanine7 anti-mouse F4/80 Antibody	Biolegend	123114
PE/Dazzle™ 594 anti-mouse CD206 (MMR) Antibody	Biolegend	141732
APC anti-mouse I-A/I-E Antibody	Biolegend	107613
PE anti-mouse NOS2 (iNOS) Antibody	Thermo Scientific	12-5920-82
Brilliant Violet 605™ anti-mouse TNF-α Antibody	Biolegend	506329
PE/Dazzle™ 594 anti-mouse IFN-γ Antibody	Biolegend	505846
PE anti-human/mouse granzyme B recombinant Antibody	Biolegend	372208
Pacific Blue™ anti-mouse CD45 Antibody	Biolegend	157211
PE/Cyanine7 anti-mouse CD252 (OX40L) Antibody	Biolegend	108813
PerCP/Cyanine5.5 anti-mouse IL12/IL23 p40 Antibody	Biolegend	505211
PE/Dazzle™ 594 anti-mouse CD86 Antibody	Biolegend	105041
FITC anti-mouse H-2D^{b} Antibody	Biolegend	111505
Brilliant Violet 605™ anti-mouse CD279 (PD-1) Antibody	Biolegend	135220
PE anti-mouse CD366 (Tim-3) Antibody	Biolegend	134003
PerCP/Cyanine5.5 anti-mouse/human CD45R/B220 Antibody	Biolegend	103235
PerCP/Cyanine5.5 anti-mouse NK-1.1 Antibody	Biolegend	108727
Brilliant Violet 510™ anti-mouse CD11c Antibody	Biolegend	117337
PE/Cyanine7 anti-mouse CD45.1 Antibody	Biolegend	110729
APC anti-mouse/human CD44 Antibody	Biolegend	103012
FITC anti-mouse CD62L Antibody	Biolegend	104406

1. Zhang, H., Sturchler, E., Zhu, J., Nieto, A., Cistrone, P.A., Xie, J., He, L., Yea, K., Jones, T., Turn, R., et al. (2015). Autocrine selection of a GLP-1R G-protein biased agonist with potent antidiabetic effects. Nat. Commun. *6*, 8918.
